# Higher-order trade-offs in hypergraph community detection

**DOI:** 10.1126/sciadv.aef2184

**Published:** 2026-08-07

**Authors:** Jiaze Li, Michael T. Schaub, Leto Peel

**Affiliations:** ^1^ Department of Data Analytics and Digitalisation, Maastricht University, Maastricht, Netherlands.; ^2^ Department of Computer Science, RWTH Aachen University, Aachen, Germany.; ^3^ Department of Biology, RWTH Aachen University, Aachen, Germany.; ^4^ Center for Computational Life Sciences, RWTH Aachen University, Aachen, Germany.

## Abstract

Extending community detection from pairwise networks to hypergraphs introduces fundamental theoretical challenges. Hypergraphs exhibit structural heterogeneity with no direct graph analog: Hyperedges of varying orders can connect nodes across communities in diverse configurations. Crucially, this means that any hypergraph community detection algorithm must make a choice regarding which order or balance of hyperedges it prefers to maintain or split during partitioning. We address these challenges by developing a unified framework for community detection in nonuniform hypergraphs under the hypergraph stochastic block model. We introduce a general signal-to-noise ratio that enables a quantitative analysis of trade-offs unique to higher-order networks, such as which hypergedges we choose to split across communities and how we choose to split them. Building on this framework, we derive a Bethe Hessian operator for nonuniform hypergraphs that provides efficient spectral clustering with principled model selection. We characterize the resulting spectral detectability threshold and compare it to belief propagation limits, showing the methods coincide for uniform hypergraphs but diverge in nonuniform settings. Synthetic experiments confirm our analytical predictions and reveal systematic biases toward preserving higher-order and balanced shape hyperedges. Application to empirical data demonstrates the practical relevance of these higher-order detectability trade-offs in real-world systems.

## Teaser

A new analysis outlines the necessary trade-offs for detecting communities in hypergraphs.

## INTRODUCTION

Community detection is central to interpreting mesoscale network structure, revealing groups of nodes with similar connectivity patterns that provide a coarse-grained summary often aligned with functional organization (*1*, *2*). In the classical setting of dyadic, or pairwise, networks, this intuition translates most commonly into identifying densely connected subgraphs, and a broad range of algorithmic and theoretical tools have been developed, including modularity maximization (*3*), spectral clustering (*4*), and probabilistic generative models such as the stochastic block model (SBM) (*5*).

The SBM has enabled a comparatively mature theoretical understanding of community detection, particularly in the case of sparse graphs. These theoretical contributions include sharp detectability thresholds, near-optimal inference algorithms, and closely related efficient spectral methods based on the nonbacktracking and the Bethe Hessian (BH) operators (*6*–*8*).

Yet, many complex systems cannot be faithfully represented by pairwise interactions alone. In domains ranging from scientific collaboration and human contact patterns to microbial consortia and chemical reaction networks, interactions regularly involve more than two entities at a time, reflecting intrinsic multiway dependencies (*9*–*11*). Recognizing the importance of such higher-order interactions has led to growing interest in higher-order network representations and corresponding models and algorithms, with hypergraphs emerging as a natural and widely used framework; see, e.g., recent reviews (*9*, *12*, *13*). Here, we consider aspects of applying the established utility of community detection to the broadly applicable setting of hypergraphs.

**Fig. 1.**
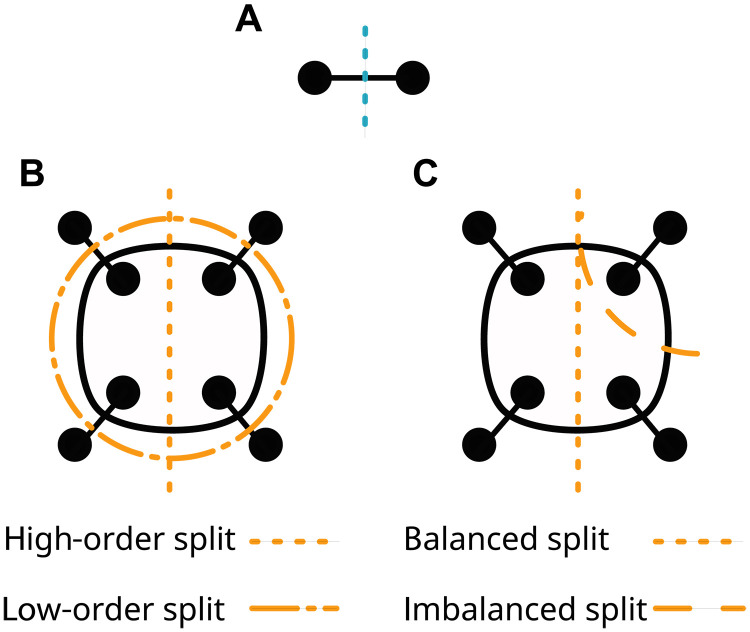
Different configurations of community structure in graphs and hypergraphs. (**A**) In dyadic networks, there is a single type of edge connecting pairs of nodes and edges can only be split across communities in one way. (**B**) In hypergraphs, hyperedges of different orders connect varying numbers of nodes, and we must choose which order of hyperedge to split across communities, e.g., do we prefer to preserve the higher-order 4-hyperedges or the lower-order 2-hyperedges? (**C**) Hyperedges can connect more than two nodes and can be split across communities in multiple ways. Here, we consider two different shapes of a 4-hyperedge, a balanced shape, and an imbalanced shape.

Transferring the notion of community structure from graphs to hypergraphs is far from straightforward. In dyadic networks, community structure can be largely described by edge density: The strength of community structure corresponds to the relative difference in the density of edges within communities compared to across communities. Hyperedges connect varying numbers of nodes, and these multiway interactions can couple nodes from different communities in a variety of configurations. Figure 1 illustrates some examples of these different configurations in community partitions in graphs and hypergraphs. The resulting structural heterogeneity has no direct analog in graphs. This implies that any community detection algorithm, regardless of its specific mechanism, must make a choice regarding which hyperedge orders and “shapes” (i.e., how hyperedges split across communities) to prioritize or preserve in the resulting partition. This leads to fundamental questions: How do these choices jointly influence the recoverability of planted structure, and how can we systematically characterize the resulting trade-offs? Unlike the graph setting, where detectability and algorithmic optimality are now relatively well understood, the theoretical understanding of community detection in hypergraphs remains much less developed.

In particular, higher-order interactions introduce previously unidentified trade-offs in describing strength of community structure that have not been characterized systematically so far. Existing work has focused on extending graph-based community detection methods to hypergraphs without explicitly addressing these higher-order trade-offs. While different algorithms may make different choices, we argue that understanding the nature and impact of these choices is essential for any application of community detection to hypergraphs. The study of hypergraph analogs of modularity maximization (*14*), spectral methods based on hypergraph Laplacians (*15*), nonbacktracking operators (*16*), and approximate-inference schemes derived from belief propagation (BP) (*17*, *18*) provide useful building blocks, but a more unified picture of detectability in general nonuniform hypergraphs is still lacking.

In this work, we take an important step toward such a picture by studying the detectability of community structure in hypergraphs under the hyper stochastic block model (HSBM) (*16*, *17*). In the HSBM, nodes are assigned to latent communities, and hyperedges of various orders are generated according to community-dependent probabilities. This framework generalizes the SBM to higher-order interactions and provides a clear link between observed hypergraph structure and latent communities, enabling principled evaluation of community detection methods via their ability to recover planted structure.

The HSBM also allows us to formulate and analyze detectability of communities in hypergraphs. Previous work has derived BP-based detectability thresholds for uniform HSBMs, where all hyperedges have the same order (*17*), and for specific cases of nonuniform settings (*16*, *18*). Here, we generalize these results by introducing a unified signal-to-noise ratio (SNR) for hypergraphs that applies to arbitrary mixtures of hyperedge orders and any number of communities. This SNR quantifies the relative strength of different hyperedge orders and shapes and thereby provides an important tool to analyze the previously unidentified trade-offs that arise in hypergraph communities.

Our algorithmic approach builds on spectral clustering via the BH (*8*). We derive a BH operator for nonuniform hypergraphs that serves as a symmetric, *n* × *n* linearization of BP for the HSBM and retains a spectral correspondence to the hypergraph nonbacktracking operator (*16*, *19*). This correspondence inherits principled model-order selection via negative BH eigenvalues and provides node-level embeddings via BH eigenvectors while offering substantial computational advantages over nonbacktracking operators. Using our BH formulation, we characterize a spectral detectability threshold in terms of our SNR and compare it to a more general theoretical detectability limit derived for the same HSBM family using BP. We show that the two thresholds coincide in the uniform case, implying that BH-based spectral clustering reaches the theoretical limit, whereas for nonuniform HSBMs, the BH spectral threshold is strictly weaker.

Synthetic experiments confirm these analytical predictions and further highlight systematic biases in BH-based clustering according to the order and shape of hyperedges, which we can explain in terms of our analytical SNR framework. In particular, we identify situations where partitions favored by higher-order or more balanced shape hyperedges dominate the detected community structure, even when alternative partitions are equally or more strongly supported by lower-order interactions. These phenomena exemplify detectability trade-offs that are unique to higher-order networks. Last, we apply our method to empirical hypergraphs, demonstrating its scalability, interpretability, and the practical relevance of these higher-order trade-offs in real-world data.

## COMMUNITY DETECTION AND DETECTABILITY LIMITS FOR HYPERGRAPHS

Consider a hypergraph $G_{H}$ with a set of nodes V={1,…,n} and a set of hyperedges $E\subseteq \left\{e_{j}|e_{j}\subseteq V,j=1,\ldots ,m\right\}$ containing ∣E∣=m hyperedges. We can represent such a hypergraph using an incidence matrix $H\in {\left\{0,1\right\}}^{n\times m}$, whose rows are indexed by the nodes and columns are indexed by the hyperedges (*15*). Specifically, for a node i∈V and a hyperedge $e_{j}\in E$, the value $H_{\mathrm{ij}}$ is

$$H_{\mathrm{ij}}=\left\{\begin{array}{cc} 1 & i\in e_{j}\\ 0 & \text{otherwise} \end{array}\right.$$ (1)

The degree $d_{i}=\sum_{j}H_{\mathrm{ij}}$ of a node *i* is the total number of hyperedges that include node *i* and the order of a hyperedge $|e_{j}|=\sum_{i}H_{\mathrm{ij}}$ is the number of nodes involved in a hyperedge $e_{j}$. We refer to a hyperedge with order ∣e∣=κ as a κ-hyperedge or a hyperedge of order κ. If every hyperedge in a hypergraph is a κ-hyperedge, the hypergraph is called a κ-uniform hypergraph. Any dyadic network (graph) can be viewed as two-uniform hypergraph. Hypergraphs with mixed order hyperedges are referred to as nonuniform hypergraphs. In nonuniform hypergraphs, we can distinguish the hyperedges by their order. We denote the set of all κ-hyperedges as $E^{\left(\kappa \right)}$ and the number of κ-hyperedges as $m^{\left(\kappa \right)}=|E^{\left(\kappa \right)}|$.

### The general BH for heterogeneous hypergraphs

In this work, we consider a spectral approach for the detection of communities in hypergraphs based on the so-called Bethe-Hessian matrix for hypergraphs. Specifically, the BH matrix for nonuniform hypergraphs, which can be derived from BP and its linearization in terms of nonbacktracking matrices, is defined as follows

$$B_{\eta }^{\left(K\right)}=I-\sum_{\kappa \in K}\frac{\left(\kappa -1\right)}{\left(1-\eta \right)\left(\eta +\kappa -1\right)}D^{\left(\kappa \right)}+\sum_{\kappa \in K}\frac{\eta }{\left(1-\eta \right)\left(\eta +\kappa -1\right)}A^{\left(\kappa \right)}$$ (2)

where K denotes the set of all hyperedge orders present in the hypergraph (formally defined in the next section), η>0 is a regularization parameter, $D^{\left(\kappa \right)}$ is the diagonal degree matrix for κ-hyperedges, and $A^{\left(\kappa \right)}$ is the one-mode projection matrix for κ-hyperedges, i.e.

$$A_{\mathrm{ij}}^{\left(\kappa \right)}=\left\{\begin{array}{cc} |\{e|i,j\in e\;\text{and}|e|=\kappa \}| & i\neq j\\ 0 & i=j \end{array}\right.$$ (3)

Note that the matrix $A^{\left(\kappa \right)}$ can also be expressed in terms of the κ-hyperedge incidence matrix $H^{\left(\kappa \right)}$ as follows

$$A^{\left(\kappa \right)}=H^{\left(\kappa \right)}{\left(H^{\left(\kappa \right)}\right)}^{T}-\text{diag}\left[H^{\left(\kappa \right)}{\left(H^{\left(\kappa \right)}\right)}^{T}\right]$$ (4)

We remark that the BH has many desirable properties and deep connections to a variety of quantities and methods previously considered in the literature [see, e.g., (*8*, *16*–*18*)]. We here consider the BH in its most general form in terms of heterogeneous hypergraphs, which generalizes prior works. The derivation of the BH and its relations to the prior literature are discussed in section S2.

### The HSBM

The HSBM is a generative model for uniform (*17*, *20*) and nonuniform hypergraphs (*15*) with community structure and is a generalization of the SBM (*5*) for dyadic networks (see section S1.1 for further details and definitions related to the SBM). We can use the HSBM to generate a hypergraph $G_{H}$ with *q* communities as follows. We begin by assigning each node i∈V a community label $\psi_{i}\in \left\{1,2,\ldots ,q\right\}$.

We define the set K that collects all the possible hyperedge orders in our hypergraph. If K only has one element, then the HSBM generates a uniform hypergraph, otherwise it generates a nonuniform hypergraph. For each κ∈K, the probability of generating a κ-hyperedge is described by a κ-order tensor $\Omega^{\left(\kappa \right)}\in {\left[0,1\right]}^{q^{\kappa }}$, i.e., for any set of κ nodes {i,j,…,κ}, the probability of generating a κ-hyperedge is given by $\Omega_{\psi_{i},\psi_{j},\ldots ,\psi_{k}}^{\left(\kappa \right)}$.

For a fixed number of nodes *n* and κ∈K, the number of possible κ-hyperedges is $\left(\begin{array}{c} n\\ \kappa \end{array}\right)$. If we assume that hypergraphs are sparse, in the same way that many empirical dyadic networks are, then the number of κ-hyperedges should be $m^{\left(\kappa \right)}∼O\left(n\right)$ as n→∞.

We can then reparameterize the hyperedge connectivity within and between communities, for hyperedges of order κ

$$\Omega^{\left(\kappa \right)}=\frac{C^{\left(\kappa \right)}}{n^{\kappa -1}}$$ (5)

where $C^{\left(\kappa \right)}$ is a κ-order tensor that is constant with respect to *n*.

For convenience, we define a symmetric HSBM for both uniform and nonuniform cases in which all nodes have equal probability 1/q of being assigned to any of the *q* communities, and for any κ∈K, the elements of tensor $C^{\left(\kappa \right)}$ satisfy

$$C_{z}^{\left(\kappa \right)}=\left\{\begin{array}{cc} c_{\text{in}} & \text{if}\;z_{i}=z_{j}=\ldots =z_{\kappa }\\ c_{\text{out}} & \text{otherwise} \end{array}\right.$$ (6)

where z represent the κ-dimensional vector of community indices. Note that in this symmetric HSBM, the parameters $c_{\text{in}}$ and $c_{\text{out}}$ are the same for all hyperedge orders κ∈K, which simplifies analysis; the case of order-dependent parameters $c_{\text{in}}^{\left(\kappa \right)},c_{\text{out}}^{\left(\kappa \right)}$ is a natural extension discussed in section S1.1.

### Spectral clustering with the BH for hypergraphs

Given a (possibly nonuniform) hypergraph $G_{H}$ and its BH $B_{\eta }^{\left(K\right)}$ from Eq. 2, we detect communities by spectral clustering on $B_{\eta }^{\left(K\right)}$. As in the graph case (*8*), informative eigenvalues of the nonbacktracking operator correspond to negative eigenvalues of $B_{\eta }^{\left(K\right)}$ when η is chosen appropriately. Thus, the number of negative eigenvalues estimates the number of communities, and the associated eigenvectors provide an informative embedding of the nodes.

We now partition the hypergraph nodes according to the following procedure (*21*):

1) Choose a regularization parameter η>0. In practice, a simple degree-based rule works well: for uniform hypergraphs, set $\eta \approx \sqrt{d^{\left(\kappa \right)}\left(\kappa -1\right)}$; for nonuniform hypergraphs, set $\eta \approx \sum_{\kappa \in K}\sqrt{d^{\left(\kappa \right)}\left(\kappa -1\right)}$ (the bulk radius of the nonbacktracking spectrum; see section S2.3).

2) Let $\lambda_{1}\left(\eta \right)\leq \ldots \leq \lambda_{n}\left(\eta \right)$ be the eigenvalues of $B_{\eta }^{\left(K\right)}$. Set $\hat{q}=|\left\{i:\lambda_{i}\left(\eta \right)<0\right\}|$ as the estimated number of communities. Note that $\hat{q}$ is a data-driven suggestion; one may also specify *q* directly (e.g., if a target number of communities is known), in which case use the *q* eigenvectors corresponding to the most negative eigenvalues.

3) Form the matrix $U\in {\mathbb{R}}^{n\times \hat{q}}$ whose columns are the eigenvectors corresponding to the $\hat{q}$ most negative eigenvalues and apply *k*-means with $\hat{q}$ clusters to the rows of U to obtain community labels ${\hat{\psi }}_{i}$.

This procedure generalizes the BH spectral clustering for graphs (*8*) and uniform hypergraphs (*17*) to nonuniform hypergraphs.

#### Computational complexity

Computing *k* eigenvectors of $B_{\eta }^{\left(K\right)}$ via the Lanczos method costs $O\left[k\left(n+\text{nnz}\right)\right]$ time and O(n+nnz) space, where $\text{nnz}=\sum_{\kappa }m^{\left(\kappa \right)}$ is the total number of hyperedges across all orders. This is the same asymptotic cost as the hypergraph Laplacian but substantially lower than the nonbacktracking operator, which requires $O\left[k\sum_{\kappa }m^{\left(\kappa \right)}{\left(\kappa -1\right)}^{2}\right]$ time and space. BP, while achieving the theoretical detectability limit, additionally requires knowledge of the model parameters and costs $O\left(T\sum_{\kappa }m^{\left(\kappa \right)}\kappa^{2}\right)$ per iteration. A detailed complexity comparison is provided in section S3.

## GENERALIZED DETECTABILITY LIMITS FOR HYPERGRAPH COMMUNITY STRUCTURE

The detectability limit for community structure describes the boundary below which no algorithm can succeed in recovering the true community structure with accuracy better than random guessing. Detectability limits of community structure in dyadic networks have been extensively studied in the literature (*6*, *22*–*24*). In contrast, the theoretical understanding detectability in hypergraphs has received comparatively less attention. Early work by Angelini *et al.* (*17*) first analyzed the detectability limit of the uniform symmetric HSBM, using a theoretical approach based on BP, similar to the analysis in dyadic networks (*6*). Subsequently, Chodrow *et al.* (*16*) analyzed the spectral features of both the nonbacktracking matrix (NB) and the Jacobian matrix of belief propagation (BP Jacobian). They established two limits related to the spectral clustering based on these two matrices for the nonuniform symmetric HSBM. However, their analysis was restricted to the case of only *q* = 2 communities. Chodrow *et al.* (*16*) concluded that the limit derived from the NB is weaker than the limit from the BP Jacobian. More recently, the work by Ruggeri *et al.* (*18*) derived the theoretical detectability limit using BP for a specialized variant called HySBM, a model structurally constrained by deriving all κ-order affinity tensors from a single underlying probability matrix, thereby limiting the independent modeling of different hyperedge order probabilities.

Here, we substantially extend these contributions. First, we reparameterize and generalize the spectral detectability limit, build on the work of Chodrow *et al.* (*16*) and Angelini *et al.* (*17*) for the nonuniform symmetric HSBM with an arbitrary number of communities. Second, we use a methodology similar to that of Ruggeri *et al.* (*18*) to derive the theoretical detectability limit with BP for the nonuniform symmetric HSBM. Last, by comparing these two derived theoretical boundaries, we conclude that the spectral clustering limit is precisely equal to the theoretical limit in the uniform HSBM case but is demonstrably weaker in the more complex nonuniform HSBM case.

### Detectability limit for spectral methods on general hypergraphs

We can describe the detectability limit for spectral clustering with the BH in terms of the average κ-in-degree $d_{\text{in}}^{\left(\kappa \right)}$ and κ-out-degree $d_{\text{out}}^{\left(\kappa \right)}$. To this end, we define the average κ-in-degree $d_{\text{in}}^{\left(\kappa \right)}$ as the expected number of κ-hyperedges incident on a node in which all κ nodes belong exclusively to the same community. Analogously, the average κ-out-degree $d_{\text{out}}^{\left(\kappa \right)}$ is the expected number of hyperedges that connect to at least one node belonging to a different community. Now, consider a network generated from a symmetric HSBM with *q* equal-sized communities and symmetric tensor C satisfying (Eq. 6). Then, $d_{\text{in}}^{\left(\kappa \right)}$ and $d_{\text{out}}^{\left(\kappa \right)}$ can be computed as (see eqs. S62 and S63 in section S4 for details)

$$d_{\text{in}}^{\left(\kappa \right)}=\frac{c_{\text{in}}}{q^{\kappa -1}\left(\kappa -1\right)!}d_{\text{out}}^{\left(\kappa \right)}=\frac{c_{\text{out}}}{q^{\kappa -1}\left(\kappa -1\right)!}$$ (7)

and the average κ-degree for the node is

$$d^{\left(\kappa \right)}=d_{\text{in}}^{\left(\kappa \right)}+\left(q^{\kappa -1}-1\right)d_{\text{out}}^{\left(\kappa \right)}$$ (8)

The detectability limits for spectral methods previously derived in the literature (*16*, *17*) can now be generalized to the nonuniform symmetric HSBM with any *q* as follows (for details, see section S4)

$${\text{SNR}}_{\text{BH}}≔\frac{{\left[\sum_{\kappa \in K}\left(\kappa -1\right)\left(d_{\text{in}}^{\left(\kappa \right)}-d_{\text{out}}^{\left(\kappa \right)}\right)\right]}^{2}}{\sum_{\kappa \in K}\left(\kappa -1\right)d^{\left(\kappa \right)}}=1$$ (9)

### Detectability limit in general hypergraphs

We also derive the theoretical detectability limit with BP for nonuniform symmetric HSBM, using a similar methodology as in the work by Ruggeri *et al.* (*18*). To establish the general detectability limit, we first determine two essential characteristics of the network structure: the average degree *d* of a node and the average order $\hat{\kappa }$ of a hyperedge. Both the average degree *d* and the average order $\hat{\kappa }$ can be expressed on the basis of the order κ average degree $d^{\left(\kappa \right)}$ given in Eq. 8. The average degree *d* is directly calculated by summing the average degrees across all hyperedge orders

$$d=\sum_{\kappa \in K}d^{\left(\kappa \right)}$$ (10)

where the average order $\hat{\kappa }$ can be expressed as (see section S5.4.2 for details)

$$\hat{\kappa }=\frac{d}{\sum_{\kappa \in K}\frac{d^{\left(\kappa \right)}}{\kappa }}$$ (11)

Now, the detectability for BP on the nonuniform symmetric HSBM can be explicitly expressed as

$${\text{SNR}}_{\text{BP}}≔d\left(\hat{\kappa }-1\right)\prod_{\kappa \in K}{\left(\frac{d_{\text{in}}^{\left(\kappa \right)}-d_{\text{out}}^{\left(\kappa \right)}}{d^{\left(\kappa \right)}}\right)}^{2\frac{\hat{\kappa }d^{\left(\kappa \right)}}{\mathrm{d\kappa}}}=1$$ (12)

Analogously, to prior works (*6*), we posit that this represents the theoretical detectability limit for nonuniform hypergraphs with *q* communities. For simplicity, the detailed derivation of Eq. 12 is deferred to section S5.

In the uniform HSBM, The ${\text{SNR}}_{\text{BP}}$ (Eq. 12) will degenerate to ${\text{SNR}}_{\text{BH}}$ (Eq. 9). That means that in the uniform case, spectral clustering (e.g., using the BH or the NB) can detect community structure down to the same threshold as BP. In the nonuniform case, however, the spectral detectability threshold is weaker than the BP threshold. Here, “weaker detectability” means that the spectral method requires a higher SNR to succeed: At the BP threshold (${\text{SNR}}_{\text{BP}}=1$), the BH has ${\text{SNR}}_{\text{BH}}<1$ and therefore cannot detect communities better than random guessing, while BP can still succeed. Equivalently, the BH threshold $\epsilon_{\text{BH}}^{*}$ is strictly smaller than $\epsilon_{\text{BP}}^{*}$, meaning that there is a regime in which BP detects structure but the BH spectral method does not. It is important to note that being above this threshold does not guarantee the detection of all community structures; instead, it ensures that the recovered partition is correlated with the true structure, which can include, for example, a coarser hierarchical partition or the detection of dominant (majority) communities (*25*, *26*).

We validate the thresholds ${\text{SNR}}_{\text{BH}}=1$ and ${\text{SNR}}_{\text{BP}}=1$ empirically in the following experiment on synthetic hypergraphs. We fix the average degree *d* of a hypergraph and vary the ratio $\epsilon =\frac{c_{\text{out}}}{c_{\text{in}}}$ to control the community strength. When ϵ is small, the community structure is strong, and when ϵ is large, the community structure is weak. We denote the critical ϵ for which ${\text{SNR}}_{\text{BH}}=1$ as $\epsilon_{\text{BH}}^{*}$ and the critical ϵ that make ${\text{SNR}}_{\text{BP}}=1$ as $\epsilon_{\text{BP}}^{*}$. We use the adjusted mutual information (AMI) (*27*) to evaluate the community detection result. The AMI score lies in the range [0,1], where 1 indicates perfect agreement between the detected communities and the true communities, while 0 indicates that the detected communities are no better than random guessing.

**Fig. 2.**
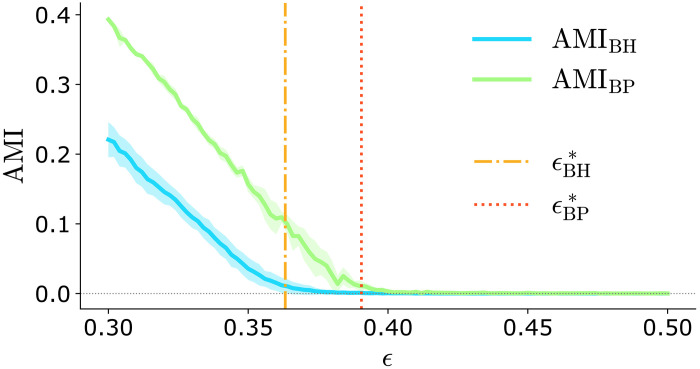
Spectral methods exhibit a weaker detectability limit in nonuniform hypergraphs. An experiment with nonuniform hypergraphs (n=30,000 nodes, *q* = 3 communities, K={2,3}, and mean degree *d* = 10). For each ϵ, we generate independent HSBM samples and run BH spectral clustering 100 times (with random initialization) and BP 5 times; solid lines show means, and shaded bands show 95% confidence intervals. BH runs use random Lanczos initialization; BP is deterministic given the graph, so trials correspond to independent graph samples. The yellow and red vertical dashed lines show $\epsilon_{\text{BH}}^{*}$ and $\epsilon_{\text{BP}}^{*}$ respectively, where ${\text{SNR}}_{\text{BH}}=1$ and ${\text{SNR}}_{\text{BP}}=1$.

In Fig. 2, we see the results of experiments conducted on nonuniform hypergraphs with *q* = 3 communities. We see that $\epsilon_{\text{BH}}^{*}$ indicates well the point that AMI_BH_ transitions from zero to nonzero. Similarly, $\epsilon_{\text{BP}}^{*}$ identifies the transition of AMI_BP_ from zero to nonzero.

### Using the SNR in practice

On synthetic HSBM data, ${\text{SNR}}_{\text{BH}}$ and ${\text{SNR}}_{\text{BP}}$ are computed directly from the model parameters. On real data, one may obtain an empirical SNR estimate post hoc: first, obtain community assignments via spectral clustering and then compute $d_{\text{in}}^{\left(\kappa \right)}$ and $d_{\text{out}}^{\left(\kappa \right)}$ from the partition (counting within- and across-community hyperedges per order). The resulting empirical SNR serves as a diagnostic heuristic: SNR≫1 indicates robust structure; SNR≈1 signals proximity to detectability boundary; SNR≪1 suggests cautious interpretation. This heuristic does not require knowledge of ground-truth communities and complements the qualitative analysis of real-data partitions.

## ADDITIONAL TRADE-OFFS FOR DETECTING HYPERGRAPH COMMUNITY STRUCTURE

In dyadic networks, community structure is most commonly characterized by assortativity: a higher density of edges within communities compared to the edge density between communities. (Disassortative structure, where edges are more frequent between communities, is also possible but less common in practice.) Detecting communities in this assortative setting involves identifying a partition that maximizes within-community edge density while minimizing cross-community edges.

However, for higher-order networks, even this relatively simple density-based notion of community structure is less straightforward to conceptualize, due to the fact that hyperedges can connect more than two nodes and that hyperedges within the same hypergraph can vary in size. For instance, consider the case of detecting a partition of a network into two communities. An edge connecting two nodes that crosses a community boundary will always have one node in each community. For a hyperedge connecting three nodes that crosses a community boundary, there will be one node in one community and two nodes in another community. However, for a hyperedge connecting four nodes that crosses a community boundary, there are two possible configurations: It can connect one node in one community with three nodes in another community, or it can connect two nodes in each community. This observation raises the question of the relationship between these different configurations and the strength of community structure in hypergraphs. To probe this relationship further, we conduct experiments on hypergraphs in which we plant two “competing” community structures and see which one is easier to detect.

### Shape preference in hypergraph community structure

**Fig. 3.**
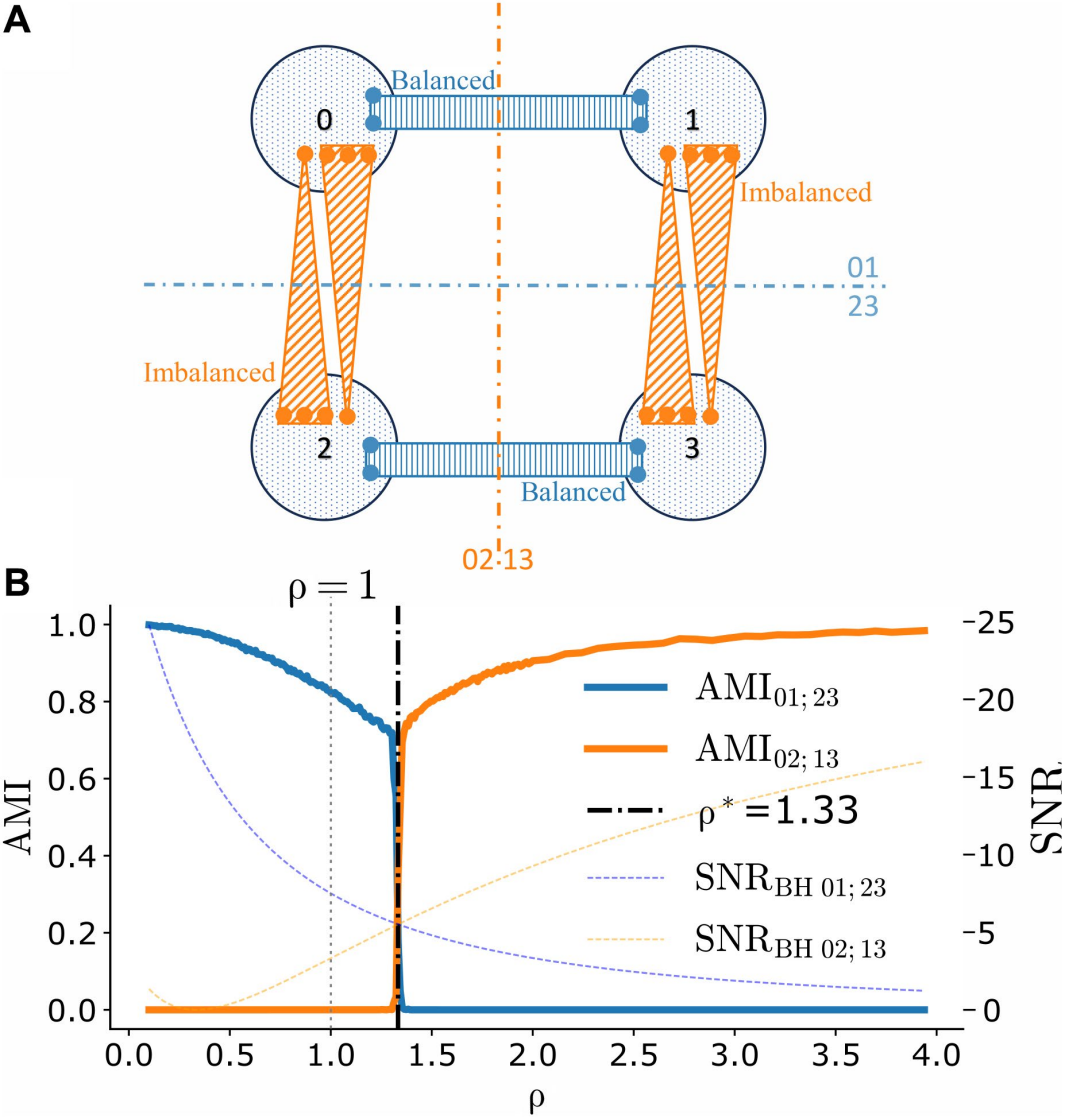
Hyperedge shape preferences in community detection. (**A**) An illustration of the planted community structure in hypergraphs used in experiments to explore the effect of hyperedge shape. All the hyperedges are four-order and connect two of the four communities. The balanced hyperedges are represented by rectangle shapes between communities {0,1} or {2,3}. The imbalanced hyperedges are represented by triangle shapes between communities {0,2} or {1,3}. (**B**) Experimental results with *n* = 8000 nodes, *d* = 10 mean node degree, and uniform four-order hyperedges. We plot ${\text{AMI}}_{01;23}$ and ${\text{AMI}}_{02;13}$ as we vary ρ, the ratio of imbalanced to balanced hyperedges. The vertical dashed line at ρ = 1 marks equal numbers of each type. The plot shows that at ρ = 1 the partition 01;23 (imbalanced split) is preferred, i.e., the algorithm tends to split imbalanced hyperedges and preserve balanced ones (2 nodes in one community, 2 in the other).

We first explore how different configurations of hyperedges crossing community boundaries affect the strength of community structure in hypergraphs. We refer to these different configurations as the shape of a hyperedge crossing community boundaries. We consider uniform four-order hypergraphs with two planted community structures that differ only in the shape of the hyperedges crossing community boundaries: balanced hyperedges that connect two nodes in each community and imbalanced hyperedges that connect one node in one community with three nodes in another community. Figure 3A illustrates the experimental setup. Each hypergraph has four planted communities, where hyperedges connecting communities {0,1} and {2,3} are balanced, while hyperedges connecting communities {0,2} and {1,3} are imbalanced. We then apply spectral clustering with *q* = 2 (deliberately below the true *q* = 4) to force the algorithm to choose between the two competing partitions: {0,1}∣{2,3} versus {0,2}∣{1,3}. Note that the BH has 4 negative eigenvalues in this setting, but we manually override *q* to 2 to probe relative detectability. We vary the ratio $\rho =\frac{m^{\text{imbalanced}}}{m^{\text{balanced}}}$ of imbalanced to balanced hyperedges.

Following the rationale of (*25*), we predict the relative detectability of the two configurations by calculating which configuration yields a higher value for ${\text{SNR}}_{\text{BH}}$ (Eq. 9). Figure 3B shows the results of the experiment, where we plot the AMI of the detected two-community partition with respect to the planted balanced and imbalanced configurations as a function of ρ. We find that the balanced configuration is easier to detect than the imbalanced one, i.e., there is a preference to split the hyperedges such that we have three nodes in one community and one node in another community, relative to splitting the hyperedges such that we have two nodes in each community. This result indicates that preserving more nodes of a hyperedge within the same community creates a stronger community structure. The transition point where the two configurations are equally detectable aligns well with the prediction based on ${\text{SNR}}_{\text{BH}}$ and, in this case, occurs at $\rho^{*}=1.33$, indicating that we require 33% more imbalanced hyperedges to achieve the same SNR as balanced hyperedges.

### Order preference in hypergraph community structure

Now, we investigate how hyperedges of different orders crossing community boundaries affect the strength of community structure in hypergraphs. Similar to the previous experiment, we consider nonuniform hypergraphs with planted community structures that differ only in the order of hyperedges crossing community boundaries: low-order hyperedges of order κ and high-order hyperedges of order $\kappa^{*}$.

Each hypergraph has four planted communities, where hyperedges connecting communities {0,1} and {2,3} are of order $\kappa^{*}$, while hyperedges connecting communities {0,2} and {1,3} are of order $\kappa <\kappa^{*}$. As before, we apply spectral clustering with *q* = 2 to probe which of the two competing partitions is preferred. We vary the ratio $\rho =\frac{m^{\left(\kappa \right)}}{m^{\left(\kappa^{*}\right)}}$ of low-order to high-order hyperedges. Following the same rationale as before, we predict the relative SNR of the two configurations by calculating which configuration yields a higher value for ${\text{SNR}}_{\text{BH}}$ (Eq. 9).

This time, however, the difference in hyperedge orders means that we need to adjust ${\text{SNR}}_{\text{BH}}$ accordingly. In the case of nonuniform hypergraphs, we find that the SNR for each configuration is equal when

$$\frac{{\text{SNR}}_{\text{BH}02;13}}{\kappa +1}=\frac{{\text{SNR}}_{\text{BH}01;23}}{\kappa^{*}+1}$$ (13)

**Fig. 4.**
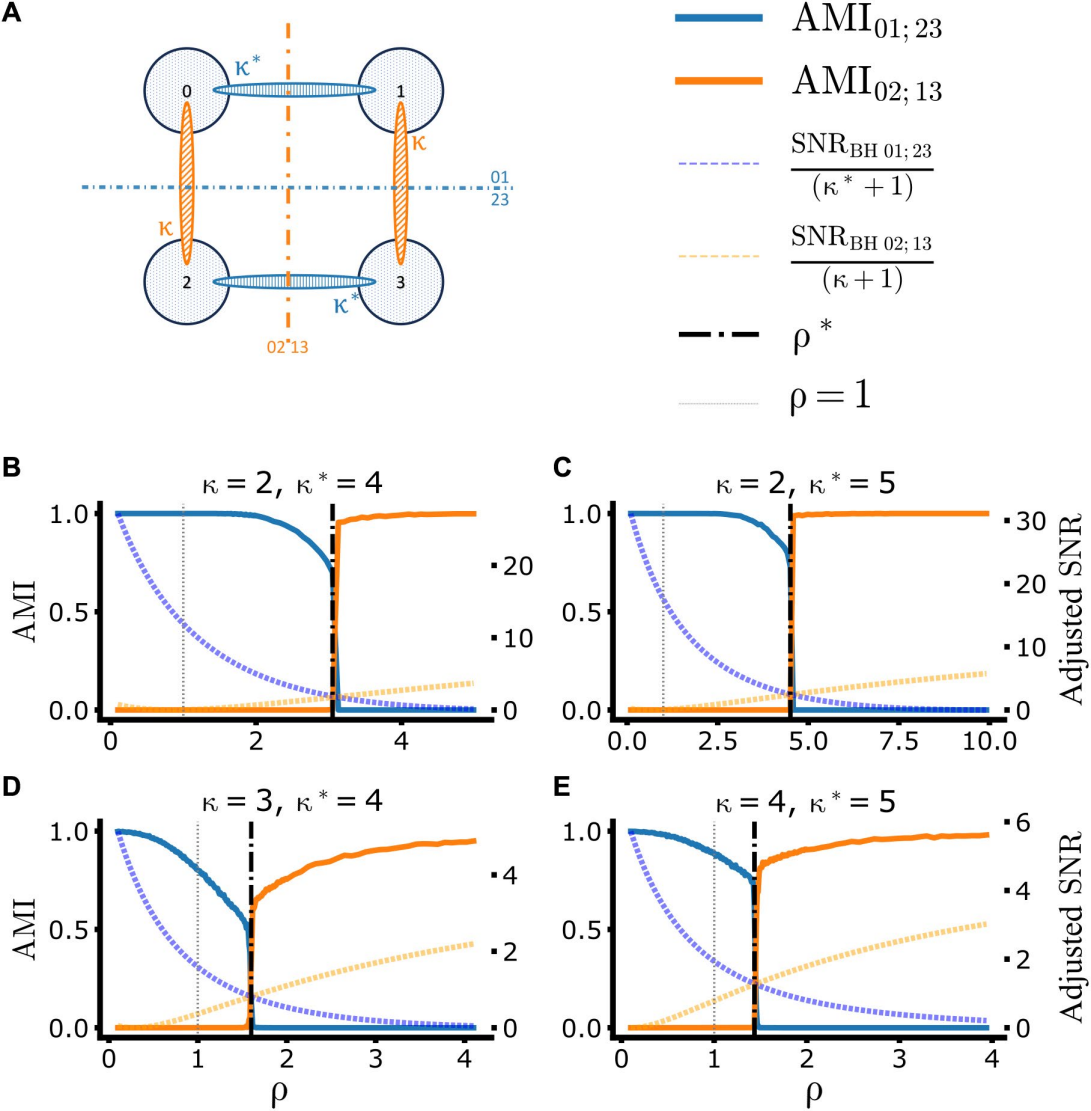
Hyperedge order preferences in community detection. (**A**) Synthetic hypergraphs with four planted communities and all the hyperedges between communities {0,1} or {2,3} are of order $\kappa^{*}$, and the hyperedges between communities {0,2} or {1,3} are of order κ. All hypergraphs have *n* = 8000 nodes. The average degree is either *d* = 50 (**B** and **C**) or *d* = 10 (**D** and **E**). We see the ${\text{AMI}}_{01;23}$ and ${\text{AMI}}_{02;13}$ as we vary ρ the ratio of high-order to low-order hyperedges. We observe a preference for preserving higher-order $\kappa^{*}$-hyperedges and splitting lower-order κ-hyperedges.

Figure 4 shows the results of the experiment, where we plot the AMI of the detected two-community partition with respect to the planted low-order and high-order configurations as a function of ρ. We find that the high-order configuration is easier to detect than the low-order one, i.e., there is a preference to split the lower-order hyperedges across community boundaries over splitting higher-order ones. This result indicates that hyperedges of lower order create a stronger community structure.

## EXPERIMENTS ON EMPIRICAL DATA

We test the BH on some hypergraphs constructed from empirical data: two human contact interaction datasets and one user review dataset. The two human contact interaction datasets are from face-to-face interactions using wearable radio frequency identification (RFID) tags in a primary school (*28*) and a high school (*29*). Students and teachers are represented as nodes, and a hyperedge is constructed for each group of individuals who all have a recorded interaction with each other at a given time. The nodes have labels that indicate the school class in which they belong. The user review dataset is from the Yelp website for business reviews in the United States and Canada (*30*). The Yelp dataset contains reviews for numerous businesses across 13 states in the United States and one province in Canada. We use nodes to represent the businesses and hyperedges to represent groups of businesses that are reviewed by the same user. We summarize the hypergraphs constructed from these empirical datasets in Table 1.

**Table 1.** Empirical data.

Hypergraph	Nodes	Hyperedges	Max order
Primary school	242	12,704	5
High school	327	7,818	5
Yelp	15,034	851,921	3,048

### Human contact interaction

**Fig. 5.**
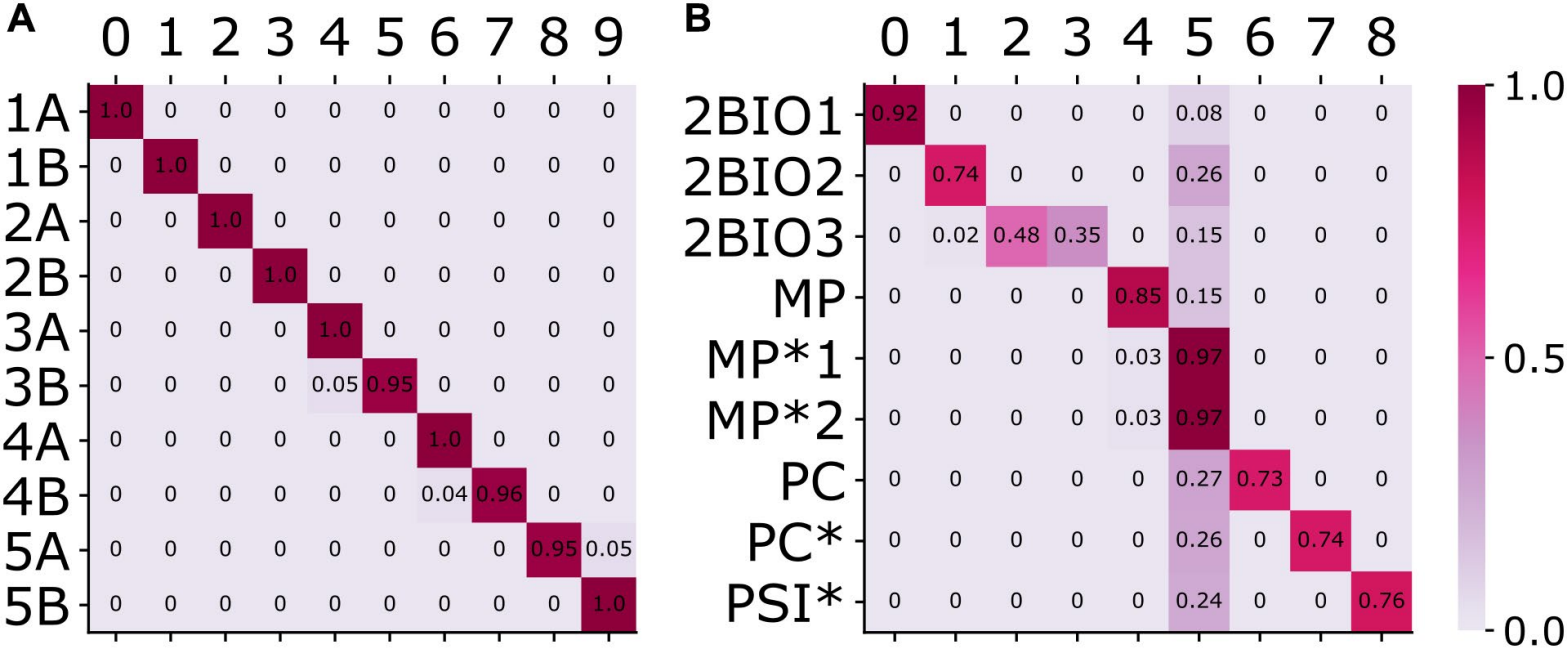
Comparison between detected communities and class structure in human contact hypergraphs. Confusion matrices of communities detected and school classes in hypergraphs of human contact interactions in (**A**) the primary school and (**B**) the high school. The rows are normalized such that each entry represents the proportion of individuals in the class assigned to each community.

First, we detect communities of the primary school, which is available in (*31*), using spectral clustering with the BH and compare with the school classes. To facilitate comparison, we manually set the number of communities to match the number of classes in the school. Figure 5A shows the confusion matrix between the detected communities and the classes. We observe a high correspondence, with the vast majority of students (≥95%) from each class assigned to a single corresponding community. This result is similar to community detection results on dyadic networks constructed from the same primary school data (*28*, *32*).

Next, we perform a similar comparison on the high school data, which are available in (*33*). Figure 5B shows a more fragmented correspondence compared to the primary school data. This result deviates from the results of community detection on dyadic networks constructed from the same high school data [e.g., using the BH (*34*)], where the dense within-class connections dominate the mesoscale structure. Instead, we observe that class 2BIO3 is split into two main communities (communities 2 and 3, with 48 and 35% of its members respectively), while classes MP*1 and MP*2 are largely concentrated in community 5 (97%).

**Fig. 6.**
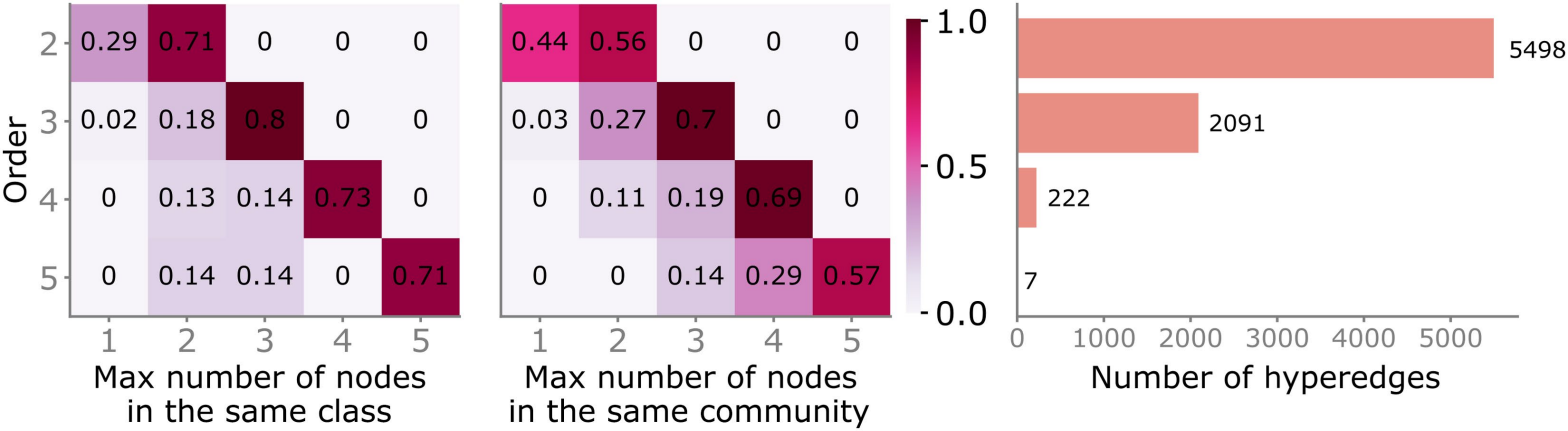
Hyperedge distributions in the high school data. The distribution of hyperedges by the maximum number of nodes in the same school class (**left**) and the maximum number of nodes in the same detected community (**middle**). The frequency of hyperedges of each order (**right**).

One might be tempted to attribute this deviation from class structure and previous results on dyadic networks as a failure of our method. However, it is important to consider that there may be multiple valid ways to partition the nodes in empirical data (*35*) and that we should expect a different community structure to emerge when we consider higher-order interactions. To better understand the composition of these hypergraph communities, we analyze the distribution of hyperedges across communities in terms of their order and shape. Figure 6 reveals that the detected communities are not simply composed of homogeneous hyperedges where all nodes belong to the same community. Instead, we observe a preference for hyperedge shapes that preserve a higher number of nodes within a single community. For example, for order-three hyperedges, the fraction of imbalanced hyperedges that connect two nodes within a community and one outside increases from 18% in the school classes to 27% in the detected communities. This quantifies the tendency of the algorithm to favor these near-homogeneous splitting shapes. The fact that higher-order hyperedges are not always favored within communities is explained by the much larger proportion of lower-order hyperedges in the data (see Fig. 6, right). These results align with our earlier findings on the preference for imbalanced and higher-order hyperedges in planted community structures.

### User reviews of businesses

**Fig. 7.**
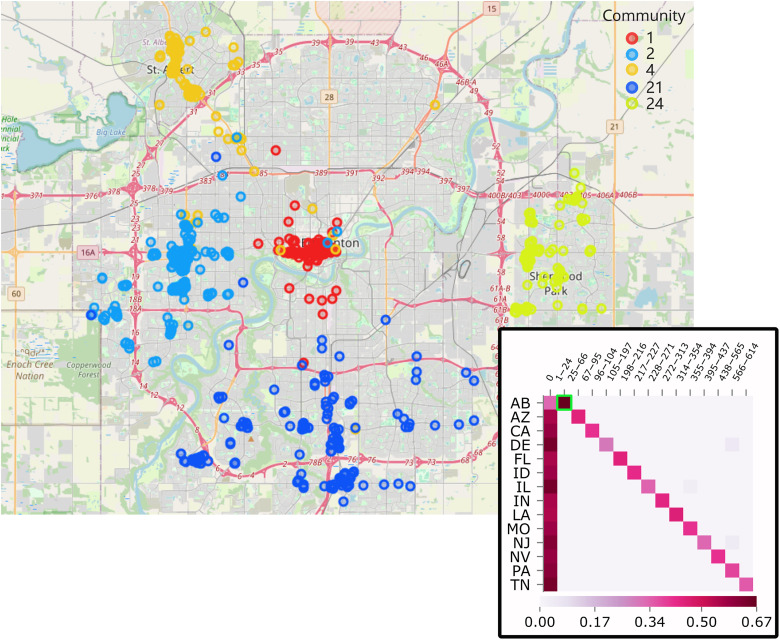
Geographical distribution of detected communities in the Yelp data. The bottom right corner is the confusion matrix of communities detected by BH-based spectral clustering with state metadata for Yelp data. We highlight some communities composed of business in the Alberta (AB) province and show their geographical distribution.

Last, we apply our method for spectral hypergraph clustering with the BH to the Yelp dataset, which is available in (*30*). Here, we determine the number of communities according to the number of negative eigenvalues of ${\text{BH}}_{\eta }$, resulting in the identification of 615 communities. Figure 7 (inset) presents a confusion matrix between the detected communities and the states in which the businesses are located; for clarity, we merge communities primarily composed of businesses from the same state. We see that most of these communities predominantly consist of businesses located within a single state, except community 0 that includes businesses across all states because these businesses are reviewed by users who review businesses across multiple states. We also find geographical organization within states, indicating a hierarchical community structure (*34*) that corresponds to different resolutions of geographical regions. Figure 7 shows the geographical distribution of businesses in some of the detected communities in the Alberta province in Canada. We see that community 1 is mainly located in the central region of Edmonton, community 2 is in the western region, community 21 is in the southern region, community 24 is in Sherwood Park, and community 4 is in St. Albert.

## DISCUSSION

Community detection in hypergraphs presents fundamental challenges that have no direct analog in dyadic networks. While pairwise networks exhibit a relatively simple trade-off between within- and across-community edge densities, hypergraphs introduce structural heterogeneity through hyperedges of varying orders that can split across communities in multiple configurations. Existing approaches to hypergraph community detection have largely overlooked a critical question: When hyperedges of different orders coexist and can be partitioned across communities in different ways, which structures are preferentially preserved by inference algorithms, and what are the implications for interpreting detected communities? This work provides a systematic characterization of these higher-order trade-offs, introducing a unified framework for understanding how hyperedge order and splitting shape fundamentally determine what community structure can be detected.

Our main theoretical contributions are a generalized SNR that quantifies the detectability of communities in hypergraphs for arbitrary mixtures of hyperedge orders and shapes and a corresponding BH operator for nonuniform hypergraphs that enables efficient spectral clustering. Critically, this SNR framework reveals that the relative detectability of different community partitions depends not only on which hyperedge orders we choose to split across communities but also on how we split them—a trade-off unique to higher-order networks that has not been considered before. We show that spectral methods exhibit systematic biases toward preserving higher-order hyperedges and those with more balanced shapes within the same community, even when alternative partitions are equally or more strongly supported by lower-order interactions. This finding has direct implications for interpreting communities in real-world hypergraphs: Detected partitions reflect not only edge density but also the order and shape preferences inherent in the detection method. Through comparison with general theoretical limits, we establish that spectral methods based on the BH achieve optimal detectability for uniform hypergraphs but exhibit a weaker threshold in nonuniform settings. Our synthetic experiments confirm these analytical predictions, while empirical applications demonstrate the practical relevance of these order and shape preferences: We recover known structure in human contact networks while revealing alternative organizational patterns that align with preferences for specific hyperedge configurations and successfully identify hierarchical geographical communities in large-scale data with more than 150,000 nodes and hyperedge orders exceeding 3000.

While we frame these results in terms of hypergraphs, they apply equally to community detection in bipartite graphs, where hypergraphs can be represented via their incidence structure and hyperedge orders correspond to the degrees of one mode. However, the hypergraph perspective provides a more natural lens for understanding these trade-offs, as the notions of order and splitting shape emerge directly from the multiway interaction structure rather than indirectly through degree heterogeneity in one mode of a bipartite network. The connection between hyperedge orders and node degrees in bipartite graphs suggests that our framework may open new directions for defining detectability limits in degree-heterogeneous networks, a problem that has only been considered in graphs for the restricted cases of two communities (*36*) or when community structure correlates with degree (*37*).

Several directions warrant further investigation. First, our current framework assumes homogeneous degree and order distributions within communities. Extending the BH to incorporate degree and order heterogeneity, analogous to degree-corrected models for dyadic networks (*38*), would broaden applicability to real-world systems. However, beyond developing these extensions, it remains an open empirical question whether degree and order correction are as relevant for hypergraph community detection as degree correction has proven to be for dyadic networks. Second, while our analysis focuses on assortative community structure, disassortative structures in hypergraphs are less straightforward to characterize than in graphs. How should we define disassortativity when hyperedges can split across communities in different shapes? The notion of hyperedge shape introduced here may provide a natural lens for addressing this question, as different splitting patterns could encode various forms of disassortative organization. Last, rigorous proofs of our conjectured detectability thresholds and SNR characterizations remain an important theoretical challenge, as does developing spectral methods that close the gap between the spectral and overall theoretical limits in nonuniform settings.

## Supplementary Materials

### This PDF file includes:

Supplementary Text Figs. S1 to S16 Table S1 References
