# Supplementary material for: Higher-order trade-offs in hypergraph community detection
Source: Sci Adv. 2026 Aug 7;12(32):eaef2184. doi: 10.1126/sciadv.aef2184 (PMC13450313; doi:10.1126/sciadv.aef2184)
Supplement: Supplementary file 1 [file sciadv.aef2184_sm.pdf]

Science Advances
AAAS

Supplementary Materials for

Higher-order trade-offs in hypergraph community detection

Jiaze Li *et al.*

Corresponding author: Leto Peel, l.peel@maastrichtuniversity.nl

Sci. Adv. **12**, eaef2184 (2026)
DOI: 10.1126/sciadv.aef2184

This PDF file includes:

Supplementary Text
Figs. S1 to S16
Table S1
References

S1 Detectability limit and the Bethe Hessian for dyadic networks

In this section, we first introduce the Stochastic Block Model, a generative model for networks with community structure. Then we review the non-backtracking matrix and Bethe Hessian matrix used for spectral clustering. Lastly, we briefly review the detectability limit of SBM.

S1.1 The stochastic block model

We consider an unweighted, undirected dyadic network G_A with node set $\mathcal{V}$ and edge set $\mathcal{E}$, where the number of nodes $|\mathcal{V}| = n$, number of edges $|\mathcal{E}| = m$, such that G_A can be represented as an adjacency matrix $\boldsymbol{A} \in \{0,1\}^{n\times n}$. For any two nodes $i, j \in \mathcal{V}$, $\boldsymbol{A}$ is defined as

$$\boldsymbol{A} = \begin{cases} 1 & \text{if } (i,j) \in \mathcal{E} \\ 0 & \text{otherwise} \end{cases} \quad . \tag{14}$$

The degree of any node is defined by the number of edges connected to that node. For a node i, its degree d_i is

$$d_i = \sum_{j\in\mathcal{V}} \boldsymbol{A}_{ij} \quad , \tag{15}$$

and the average degree of whole network as d, then

$$d = \frac{\sum_{i\in\mathcal{V}} d_i}{n} = \frac{2m}{n} \quad . \tag{16}$$

We can use the SBM to generate network G_A with q communities. We assign each node $i \in \mathcal{V}$ a community label $\psi_i \in \{1, 2, ..., q\}$. Let matrix $\boldsymbol{\Omega}$ be the edge probability matrix, $\boldsymbol{\Omega} \in [0,1]^{q\times q}$. For each pair of nodes $i, j \in \mathcal{V}$, we generate an edge (i, j) with probability $\boldsymbol{\Omega}_{\psi_i\psi_j}$. We assume the network G_A is sparse based on the observation that many empirical networks tend to have constant average degree relative to the network size. This sparsity implies that $m \sim \mathrm{O}(n)$ and we can assume the matrix $\boldsymbol{\Omega}$ has the form

$$\boldsymbol{\Omega} = \frac{\boldsymbol{C}}{n} \quad , \tag{17}$$

where $\boldsymbol{C}$ is a matrix that is constant as we vary n.

A common variant of the SBM is the symmetric stochastic block model. The symmetric SBM assumes that all q communities have the same number of nodes and the matrix $\boldsymbol{\Omega}$ is composed of two distinct probabilities: intra-community edge probability p_{in} and inter-community edge probability p_{out}. If nodes i and j belong to same community, $\psi_i = \psi_j$, then $\boldsymbol{\Omega}_{\psi_i\psi_j} = p_{\text{in}}$, otherwise $\boldsymbol{\Omega}_{\psi_i\psi_j} = p_{\text{out}}$. Similarly, the rescaled edge probability matrix $\boldsymbol{C} = n\boldsymbol{\Omega}$ also has two distinct values: $c_{\text{in}} = np_{\text{in}}$, $c_{\text{out}} = np_{\text{out}}$.

S1.2 Detectability limit of community structure in networks

The goal of community detection is to infer the community labels $\boldsymbol{\psi}$ based solely on observing the network G_A. A community structure is said to be detectable if there exists an algorithm that can infer the community labels better than random guessing. The detectability of community structure is related to the parameters of the SBM used to generate the network, specifically the differences in edge densities within and between communities. For instance, if $c_{\text{in}} = c_{\text{out}}$, the network is essentially an Erdős–Rényi random graph (39) without any community structure, making it impossible to detect communities. Conversely, when c_{in} is significantly larger than c_{out}, the communities are more densely connected internally than externally, making them easier to detect. If a community detection algorithm can detect $\boldsymbol{\psi}$ better than random guessing within polynomial time complexity, then the community structure is said to be detectable (24). This detectability threshold coincides with the Kesten-Stigum threshold (6, 40). Below the detectability threshold, no algorithm can perform better than random guessing, and the community structure is said to be undetectable.

For the symmetric SBM, the theoretical detectability threshold occurs when

$$|c_{\text{in}} - c_{\text{out}}| > q\sqrt{d} \ , \tag{18}$$

where the average degree $d = \frac{c_{\text{in}}+(q-1)c_{\text{out}}}{q}$. With a little transformation, we can obtain the threshold:

$$\text{SNR} := \frac{(c_{\text{in}} - c_{\text{out}})^2}{q^2 d} > 1 \ , \tag{19}$$

where SNR is the signal-to-noise ratio. So for any network $\mathrm{G_A}$ generated by the symmetric SBM, if SNR > 1, the community structure in $\mathrm{G_A}$ is considered detectable.

To see the SNR for general SBM (24), we first define the matrix $\boldsymbol{N}$ which is a $(q \times q)$-dimension diagonal matrix, with diagonal entries equal to the number of nodes in each community. Ordering the non-decreasing magnitude eigenvalues of matrix $\boldsymbol{N\Omega}$ as $\lambda_1, \lambda_2, ...$,the general SNR is defined as

$$\text{SNR} = \frac{\lambda_2^2}{\lambda_1} \ , \tag{20}$$

which is equivalent to the SNR (19) above for the symmetric SBM.

S1.3 The non-backtracking matrix and Bethe Hessian for dyadic networks

Spectral clustering using the non-backtracking matrix is an effective community detection method that is consistent with inference on the SBM. The non-backtracking matrix is a linearization of the belief propagation algorithm (BP), an approximate inference algorithm for the SBM. The BP can detect community structure with asymptotically optimal

accuracy (6). The Bethe Hessian matrix is a compact formulation of the non-backtracking matrix that shares the same properties of detectability of the non-backtracking matrix (8).

The non-backtracking matrix $\boldsymbol{NB}$ of a network is a matrix of dimension $2m \times 2m$ such that each row and column corresponds to a directed edge in the network. Note that the non-backtracking matrix is defined on directed edges, so in the case of an undirected network, each edge (i, j) is represented as two directed edges $i \to j$ and $j \to i$. Thus, for a network with m undirected edges, there are $2m$ directed edges in total. For the directed edges $i \to j$ and $k \to l$ in the network, the entry of the non-backtracking matrix $\boldsymbol{NB}_{i\to j,k\to l}$ is defined as

$$\boldsymbol{NB}_{i\to j,k\to l} = \begin{cases} 1 & j \neq k \text{ and } i = l \\ 0 & \text{otherwise} \end{cases} . \tag{21}$$

Following the standard approach to spectral clustering, we compute the first q eigenvectors $\boldsymbol{V}_q = [\boldsymbol{\nu}_{\cdot,1}, \boldsymbol{\nu}_{\cdot,2}, \ldots, \boldsymbol{\nu}_{\cdot,q}]$ of the non-backtracking matrix $\boldsymbol{NB}$ corresponding to the leading q eigenvalues. These eigenvectors are then used to cluster the nodes in the network into q communities. Since we are interested in clustering the n nodes of the network, rather than the $2m$ directed edges, the clustering is performed by first pooling the $2m$-length eigenvectors of $\boldsymbol{NB}$ into vectors of length n. This operation is performed using a pooling matrix which aggregate relevant components of the eigenvectors for each node. The pooling matrix $\boldsymbol{P}$ is defined as follows:

$$\boldsymbol{P}_{i,k\to l} = \begin{cases} 1 & i = l \text{ and } k \in \partial i \\ 0 & \text{otherwise} \end{cases} , \tag{22}$$

where ∂i is the set of directed edges that point to node i. The pooling matrix $\boldsymbol{P}$ effectively selects the components of the eigenvectors that correspond to the directed edges pointing to each node. The spectral clustering of the network is then performed by applying a clustering algorithm, such as k-means, to the rows of the pooled matrix $\boldsymbol{U}_q = \boldsymbol{P}\boldsymbol{V}_q$. The clustering algorithm groups the nodes based on the pooled eigenvectors, resulting in a partition of the nodes into q communities.

The construction and eigen-decomposition of the non-backtracking matrix can be expensive in terms of time and memory, especially for large networks. A better alternative is to construct the Bethe Hessian that has eigenvectors that correspond to the pooled eigenvectors $\boldsymbol{U} = [\boldsymbol{\mu}_{\cdot,1}, \boldsymbol{\mu}_{\cdot,2}, \ldots, \boldsymbol{\mu}_{\cdot,n}]$. The Bethe Hessian is defined as

$$\boldsymbol{B}_\eta = (\eta^2 - 1)\boldsymbol{I} - \eta\boldsymbol{A} + \boldsymbol{D} , \tag{23}$$

where $\boldsymbol{I}$ is identity matrix, $\boldsymbol{D}$ is diagonal matrix of node degrees, η is a regularization parameter, which typically works well with the setting $\eta = \sqrt{d}$.

The Bethe Hessian can be derived from non-backtracking matrix (7, 41). To see how, we start with the eigen decomposition of the non-backtracking matrix $\boldsymbol{NB} \cdot \boldsymbol{V} = \boldsymbol{\Lambda} \cdot \boldsymbol{V}$

(where $\mathbf{\Lambda}$ is the diagonal matrix of eigenvalues) and consider in terms of a specific directed edge $i \to j$:

$$\boldsymbol{\lambda \nu}_{i\to j} = \sum_{k\in\partial i/j} \boldsymbol{\nu}_{k\to i} \quad , \tag{24}$$

and notice that the pooling operation $\boldsymbol{\mu P} = \boldsymbol{\nu}$ can be expressed in terms of equation (24) and an additional term $\boldsymbol{\nu}_{j\to i}$,

$$\begin{aligned}
\boldsymbol{\mu}_i &= \sum_{k\in\partial i} \boldsymbol{\nu}_{k\to i} && (25)\\
&= \sum_{k\in\partial i/j} \boldsymbol{\nu}_{k\to i} + \boldsymbol{\nu}_{j\to i} && (26)\\
&= \lambda\boldsymbol{\nu}_{i\to j} + \boldsymbol{\nu}_{j\to i} \quad . && (27)
\end{aligned}$$

Similarly for node j, we have:

$$\boldsymbol{\mu}_j = \lambda\boldsymbol{\nu}_{j\to i} + \boldsymbol{\nu}_{i\to j} \quad , \tag{28}$$

then we can subtract $\boldsymbol{\mu}_i - \lambda\boldsymbol{\mu}_j$ to eliminate $\boldsymbol{\nu}_{i\to j}$:

$$\boldsymbol{\mu}_i - \lambda\boldsymbol{\mu}_j = \boldsymbol{\nu}_{j\to i} - \lambda^2\boldsymbol{\nu}_{j\to i} \quad , \tag{29}$$

and rearrange to get

$$\boldsymbol{\nu}_{j\to i} = \frac{\boldsymbol{\mu}_i - \lambda\boldsymbol{\mu}_j}{1-\lambda^2} \quad . \tag{30}$$

Substitute $\boldsymbol{\nu}_{j\to i}$ from equation (30) above into equation (25) and we get:

$$\begin{aligned}
\boldsymbol{\mu}_i &= \sum_{k\in\partial i} \frac{\boldsymbol{\mu}_i - \lambda\boldsymbol{\mu}_k}{1-\lambda^2} && (31)\\
\boldsymbol{\mu}_i &= d_i\frac{\boldsymbol{\mu}_i}{1-\lambda^2} - \frac{\lambda}{1-\lambda^2}\sum_{k\in\partial i}\boldsymbol{\mu}_k && (32)\\
(1-\frac{d_i}{1-\lambda^2})\boldsymbol{\mu}_i + \frac{\lambda}{1-\lambda^2}\sum_{k\in\partial i}\boldsymbol{\mu}_k &= 0 && (33)\\
\frac{1}{\lambda^2-1}\left[(\lambda^2-1+d_i)\boldsymbol{\mu}_i - \lambda\sum_{k\in\partial i}\boldsymbol{\mu}_k\right] &= 0 \quad . && (34)
\end{aligned}$$

Omitting the term $1/(\lambda^2-1)$, we have the matrix form:

$$\begin{aligned}
\left[(\lambda^2-1)\boldsymbol{I} + \boldsymbol{D} - \lambda\boldsymbol{A}\right]\boldsymbol{\mu} &= 0 && (35)\\
\boldsymbol{B}_\lambda\boldsymbol{\mu} &= 0 \quad , && (36)
\end{aligned}$$

where $\boldsymbol{B}_\lambda$ is the Bethe Hessian matrix with regularization parameter $\eta = \lambda$.

From equation (35) we can conclude that $\boldsymbol{\mu}$ is related with null space of $\boldsymbol{B}_\lambda$. In other words, if we set the regularization parameter $\eta = \lambda$ to an eigenvalue of $\boldsymbol{NB}$, then $\boldsymbol{\mu}$ will be an eigenvector of $\boldsymbol{B}_\lambda$ corresponding with eigenvalue 0. Note that although the above relationship does not hold when the parameter η is not an eigenvalue of $\boldsymbol{NB}$, the eigenvector of $\boldsymbol{B}_{\sqrt{d}}$ is still found to be a good approximation of $\boldsymbol{\mu}$ in practice (41).

Figure S1 gives an example spectrum of $\boldsymbol{NB}$ and $\boldsymbol{B}_\eta$ for a network generated by the symmetric SBM. The blue points in the plot above represent the eigenvalues of the non-backtracking matrix $\boldsymbol{NB}$ and the light blue circle with radius $\sqrt{d}$ indicates approximately the uninformative bulk within which most of the eigenvalues lie. The red to green points in the plot below represent the eigenvalues of the Bethe Hessian matrix $\boldsymbol{B}_\eta$ for corresponding values of η. When η is set to one of the real informative eigenvalues of $\boldsymbol{NB}$, the corresponding eigenvector $\boldsymbol{\mu}$ can be used to optimally cluster the nodes in the network (38).

When the communities are detectable, there are a few informative real eigenvalues outside the bulk, which correspond to the number of communities in the network. We see that when η is sufficiently large, all eigenvalues of $\boldsymbol{B}_\eta$ are positive. As η decreases, new negative eigenvalues appear each time η crosses a real informative eigenvalue of $\boldsymbol{NB}$. Consequently, when η is equal to the radius of the uninformative bulk, the number of negative eigenvalues of $\boldsymbol{B}_\eta$ can also be used to determine the number of communities in the network. The eigenvectors corresponding with these negative eigenvalues can be used as a good approximation of $\boldsymbol{\mu}$ corresponding with the informative eigenvalues of $\boldsymbol{NB}$. The exact et of eigenvectors $\boldsymbol{U}_q$ can be obtained by identifying the leading eigenvector of the Bethe Hessian matrix $\boldsymbol{B}_\eta$ with η set to the each of the q informative eigenvalues of $\boldsymbol{NB}$.

S2 Spectral clustering for hypergraphs

In this section, we first introduce the non-backtracking matrix for hypergraphs. Then we use this non-backtracking matrix to derive the Bethe Hessian for uniform hypergraphs and non-uniform hypergraphs.

S2.1 The non-backtracking matrix for hypergraphs

The uniform hypergraph non-backtracking matrix (17) is a generalization of the non-backtracking matrix for dyadic networks (7). Similar to the non-backtracking matrix for dyadic networks, the non-backtracking matrix for hypergraphs is defined on directed hyperedges. These directed hyperedges are simply defined from undirected hyperedges such that each node i in hyperedge e induces a directed hyperedge $i \to e$. Consequently, each undirected hyperedge of size κ induces κ directed hyperedges. The non-backtracking

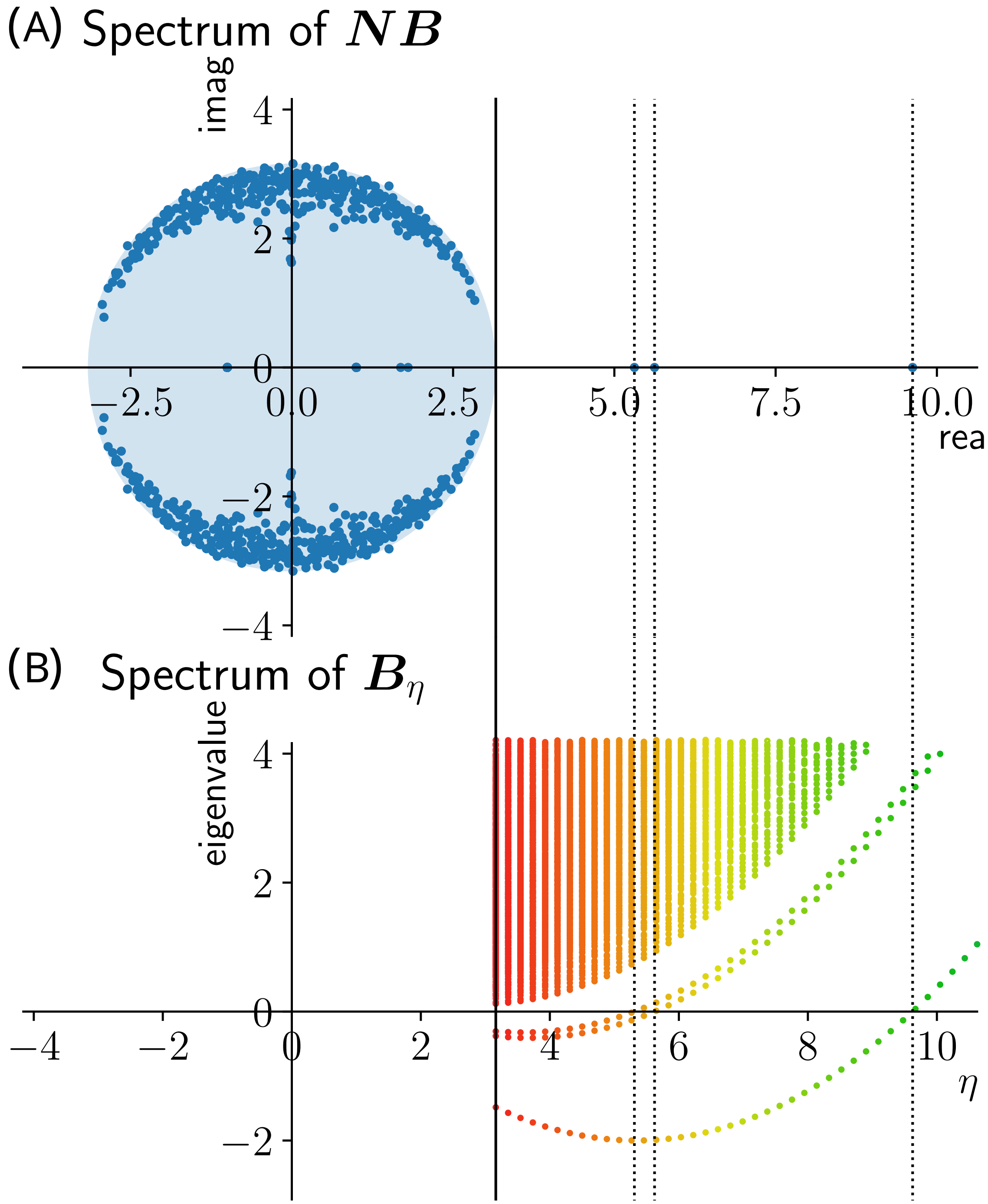

Figure S1: ***Spectra of non-backtracking and Bethe Hessian matrices in symmetric SBM networks.*** *The spectrum of (**A**) non-backtracking matrix and (**B**) Bethe Hessian matrix for a network generate by symmetric SBM. The number of nodes $n = 300$, number of communities $q = 3$, average degree $d = 10$, edge probability ratio $p_{\text{out}}/p_{\text{in}} = 0.2$. The light blue circle is the bulk with radius $\sqrt{d}$. Each column of red to green points in (**B**) is the spectrum of $\boldsymbol{B}_\eta$ where η is x-axis value. Here the spectrum of $\boldsymbol{B}_\eta$ is scaled for better presenting.*

matrix, $\boldsymbol{NB}^{(\kappa)}$, of a κ-uniform hypergraph is defined as follows. For two κ-hyperedges e_1 and e_2, if $i \in e_1$ and $j \in e_2$, the non-backtracking matrix is given by

$$\boldsymbol{NB}^{(\kappa)}_{i\to e_1, j\to e_2} = \begin{cases} 1 & j \in e_1/i \text{ and } e_2 \neq e_1 \\ 0 & \text{otherwise} \end{cases} , \tag{37}$$

where $i \to e_1$ is a directed hyperedge (16) if node i is in hyperedge e_1. Since each node in a hyperedge induces a directed hyperedge in the non-backtracking matrix, the dimension of the non-backtracking matrix is equal to the total sum of node-degrees, $\sum_i d_i$, which is equal to κm in the case of a κ-uniform hypergraph.

The non-backtracking matrix for a non-uniform hypergraph (16) is constructed in the same way, however, we can order the rows and columns by the order of the hyperedges,

$$\boldsymbol{NB}^{(\mathcal{K})} = \begin{bmatrix} \boldsymbol{NB}^{(2\to 2)} & \boldsymbol{NB}^{(2\to 3)} & ... & \boldsymbol{NB}^{(2\to \kappa)} \\ \boldsymbol{NB}^{(3\to 2)} & \boldsymbol{NB}^{(3\to 3)} & ... & \boldsymbol{NB}^{(3\to \kappa)} \\ ... & ... & ... & ... \\ \boldsymbol{NB}^{(\kappa\to 2)} & \boldsymbol{NB}^{(\kappa\to 3)} & ... & \boldsymbol{NB}^{(\kappa\to \kappa)} \end{bmatrix} , \tag{38}$$

where $\boldsymbol{NB}^{(\kappa'\to\kappa)}$ is the non-backtracking matrix from the κ'-hyperedges, $\mathcal{E}^{(\kappa')}$ to the κ-hyperedges, $\mathcal{E}^{(\kappa)}$. The dimension of each $\boldsymbol{NB}^{(\kappa'\to\kappa)}$ is $\kappa' m^{(\kappa')} \times \kappa m^{(\kappa)}$.

The leading q eigenvectors of the non-backtracking matrix can be used to perform spectral clustering. Since we are interested in clustering the nodes in the network, we can "pool" the eigenvectors to an n-dimensional vector $\boldsymbol{\mu}$,

$$\boldsymbol{\mu}_i = \sum_{\kappa\in\mathcal{K}} \sum_{e\in\partial_\kappa i} \boldsymbol{\nu}_{i\to e} , \tag{39}$$

and use the pooled vectors μ as the input to a clustering algorithm, e.g., k-means.

S2.2 The Bethe Hessian for hypergraphs

The construction and eigen decomposition of the non-backtracking matrix is computationally intensive in terms of time and space resources, especially for large hypergraphs. For dyadic networks, the Bethe Hessian (8) is a lower-dimensional symmetric matrix that captures the same desirable spectral properties of the non-backtracking matrix that makes it effective for spectral clustering. The Bethe Hessian has previously been extended for hypergraphs (19), but is only applicable if the hypergraph is uniform. Here we present a new version of the Bethe Hessian that is suitable for non-uniform hypergraphs, following a similar derivation as in the dyadic case (see Section S1).

We consider an informative eigenvector $\boldsymbol{\nu}$ and corresponding eigenvalue λ of the non-backtracking matrix $\boldsymbol{NB}^{(\mathcal{K})}$, such that $\boldsymbol{NB}^{(\mathcal{K})}\boldsymbol{\nu} = \lambda\boldsymbol{\nu}$. For a given directed hyperedge $i \to e_1$, we expand the eigenvalue equation,

$$\lambda\boldsymbol{\nu}_{i\to e_1} = \sum_{\kappa\in\mathcal{K}} \sum_{j\in e_1/i} \sum_{e_2\in\partial_\kappa j/e_1} \boldsymbol{\nu}_{j\to e_2} , \tag{40}$$

where $\partial_\kappa j$ is all κ-hyperedges including j. We can reorder the sum in equation (40) and substitute in the pooled vector $\boldsymbol{\mu}$ from (39):

$$\begin{aligned}
\lambda \boldsymbol{\nu}_{i\to e_1} &= \sum_{j\in e_1/i} \sum_{\kappa\in\mathcal{K}} \sum_{e_2\in\partial_\kappa j/e_1} \boldsymbol{\nu}_{j\to e_2} && (41)\\
&= \sum_{j\in e_1/i} \left(\sum_{\kappa\in\mathcal{K}} \sum_{e_2\in\partial_\kappa j} \boldsymbol{\nu}_{j\to e_2} - \boldsymbol{\nu}_{j\to e_1} \right) && (42)\\
&= \sum_{j\in e_1/i} \left(\boldsymbol{\mu}_j - \boldsymbol{\nu}_{j\to e_1} \right) && (43)\\
\lambda \boldsymbol{\nu}_{i\to e_1} + \sum_{j\in e_1/i} \boldsymbol{\nu}_{j\to e_1} &= \sum_{j\in e_1/i} \boldsymbol{\mu}_j \ . && (44)
\end{aligned}$$

Note that equation (44) above repeats κ times, once for each node $i \in e_1$ if e_1 is a κ-hyperedge. Summing over all of nodes in e_1, we get:

$$(\lambda + \kappa - 1) \sum_{i\in e_1} \boldsymbol{\nu}_{i\to e_1} = (\kappa - 1) \sum_{j\in e_1} \boldsymbol{\mu}_j \ , \tag{45}$$

then dividing by $(\lambda + \kappa - 1)$ and subtracting (44),

$$\begin{aligned}
(1-\lambda)\boldsymbol{\nu}_{i\to e_1} &= \frac{\kappa - 1}{\lambda + \kappa - 1} \boldsymbol{\mu}_i - \frac{\lambda}{\lambda + \kappa - 1} \sum_{j\in e_1/i} \boldsymbol{\mu}_j && (46)\\
\boldsymbol{\nu}_{i\to e_1} &= \frac{\kappa - 1}{(1-\lambda)(\lambda + \kappa - 1)} \boldsymbol{\mu}_i - \frac{\lambda}{(1-\lambda)(\lambda + \kappa - 1)} \sum_{j\in e_1/i} \boldsymbol{\mu}_j \ . && (47)
\end{aligned}$$

Now we substitute the above definition of $\boldsymbol{\nu}_{i\to e_1}$ into pooling equation (39),

$$\begin{aligned}
\boldsymbol{\mu}_i &= \sum_{\kappa\in\mathcal{K}} \sum_{e\in\partial_\kappa i} \left[\frac{\kappa - 1}{(1-\lambda)(\lambda + \kappa - 1)} \boldsymbol{\mu}_i - \frac{\lambda}{(1-\lambda)(\lambda + \kappa - 1)} \sum_{j\in e/i} \boldsymbol{\mu}_j \right] && (48)\\
&= \sum_{\kappa\in\mathcal{K}} \frac{(\kappa - 1) d_i^{(\kappa)}}{(1-\lambda)(\lambda + \kappa - 1)} \boldsymbol{\mu}_i - \lambda \sum_{\kappa\in\mathcal{K}} \sum_{e\in\partial_\kappa i} \sum_{j\in e/i} \frac{1}{(1-\lambda)(\lambda + \kappa - 1)} \boldsymbol{\mu}_j \ , && (49)
\end{aligned}$$

where $d_i^{(\kappa)} = |\partial_\kappa i|$ is the κ-degree of i, i.e., the number of κ-hyperedges that i is included in. We can rearrange the above result to set the right-hand side to zero,

$$\left(1 - \sum_{\kappa\in\mathcal{K}} \frac{(\kappa - 1) d_i^{(\kappa)}}{(1-\lambda)(\lambda + \kappa - 1)}\right) \boldsymbol{\mu}_i + \lambda \sum_{\kappa\in\mathcal{K}} \sum_{e\in\partial_\kappa i} \sum_{j\in e/i} \frac{1}{(1-\lambda)(\lambda + \kappa - 1)} \boldsymbol{\mu}_j = 0 \ . \tag{50}$$

Finally, we can rewrite the above equation in matrix form, which is the Bethe Hessian matrix for non-uniform hypergraphs:

$$\boldsymbol{B}_{\eta}^{(\mathcal{K})} = \boldsymbol{I} - \sum_{\kappa \in \mathcal{K}} \frac{(\kappa-1)}{(1-\eta)(\eta+\kappa-1)} \boldsymbol{D}^{(\kappa)} + \sum_{\kappa \in \mathcal{K}} \frac{\eta}{(1-\eta)(\eta+\kappa-1)} \boldsymbol{A}^{(\kappa)} \ , \tag{51}$$

where η is a regularization parameter, $\boldsymbol{D}^{(\kappa)}$ is the diagonal degree matrix for κ-hyperedges, and $\boldsymbol{A}^{(\kappa)}$ is the one-mode projection matrix for κ-hyperedges, i.e.,

$$\boldsymbol{A}_{ij}^{(\kappa)} = \begin{cases} |\,\{e|i,j \in e, |e| = \kappa\}\,| & i \neq j \\ 0 & i = j \end{cases} \ . \tag{52}$$

Note that the matrix $\boldsymbol{A}^{(\kappa)}$ can also be expressed in terms of the κ-hyperedge incidence matrix $\boldsymbol{H}^{(\kappa)}$ as follows:

$$\boldsymbol{A}^{(\kappa)} = \boldsymbol{H}^{(\kappa)}(\boldsymbol{H}^{(\kappa)})^T - \mathrm{diag}(\boldsymbol{H}^{(\kappa)}(\boldsymbol{H}^{(\kappa)})^T) \ . \tag{53}$$

S2.3 The spectrum of Bethe Hessian matrix

The spectrum of Bethe Hessian relates to the spectrum of non-backtracking matrix such that the negative eigenvalues of Bethe Hessian correspond to the informative eigenvalues of non-backtracking matrix. Of particular importance to the task of detecting community structure is that we preserve the relationship between the eigenvectors. Specifically, when we take an informative eigenvalue λ and corresponding eigenvector $\boldsymbol{\nu}$ and set the regularization parameter $\eta = \lambda$, then the eigenvector associated with the eigenvalue 0 of the Bethe Hessian is the pooled vector $\boldsymbol{\mu}$.

Figure S2 provides an empirical demonstration of the relationship between the spectra of the non-backtracking matrix and the Bethe Hessian for two hypergraphs with $q = 2$ communities, generated using the HSBM described earlier. Figure S2(**A**, **C**) shows the κ-uniform case with $\kappa = 3$. The radius of the bulk of uninformative eigenvalues is $\sqrt{d(\kappa-1)}$, where d is the average degree of the hypergraph. For the non-uniform case [Fig. S2(**B**, **D**)], we set $\mathcal{K} = \{2, 3, 4\}$. This time the radius of the bulk is $\sum_{\kappa \in \mathcal{K}} \sqrt{d^{(\kappa)}(\kappa-1)}$, where $d^{(\kappa)}$ is the average degree of the κ-order hyperedges, $d^{(\kappa)} = \mathrm{Tr}(\boldsymbol{D}^{(\kappa)})/n$.

Figure S2 shows the same relationship between the spectra of the non-backtracking matrix and Bethe Hessian that we see for dyadic networks in Figure S1. When parameter η is equal to the radius of uninformative bulk, then negative eigenvalues of $\boldsymbol{B}_{\eta}$ correspond one-to-one with the real informative eigenvalues of $\boldsymbol{NB}$. We can also use the eigenvectors corresponding with these negative eigenvalues of $\boldsymbol{B}_{\eta}$ to perform spectral clustering, as described in Algorithm S1.

Algorithm S1 Spectral Clustering with Bethe Hessian

Input: incidence matrix $\boldsymbol{H}$, all possible hyperedge order $\mathcal{K}$

Output: communities labels $\overrightarrow{\psi}$

Compute $\eta = \sum_{\kappa \in \mathcal{K}} \sqrt{d^{(\kappa)}(\kappa - 1)}$

Compute $\boldsymbol{B}_\eta$ by (2)

Compute spectral decomposition $\boldsymbol{B}_\eta = \boldsymbol{V}\boldsymbol{\Lambda}\boldsymbol{V}^T$.

Compute $q = |\{\boldsymbol{\Lambda}_{i,i} : \boldsymbol{\Lambda}_{i,i} < 0\}|$.

Select first q eigenvectors of $\boldsymbol{V}$ based on the increasing order of corresponding eigenvalues as $\boldsymbol{W}_q$.

Run k-means clustering on the rows of $\boldsymbol{W}_q$: $\overrightarrow{\psi} = k\text{-means}(\boldsymbol{W}_q^T, q)$

return $\overrightarrow{\psi}$

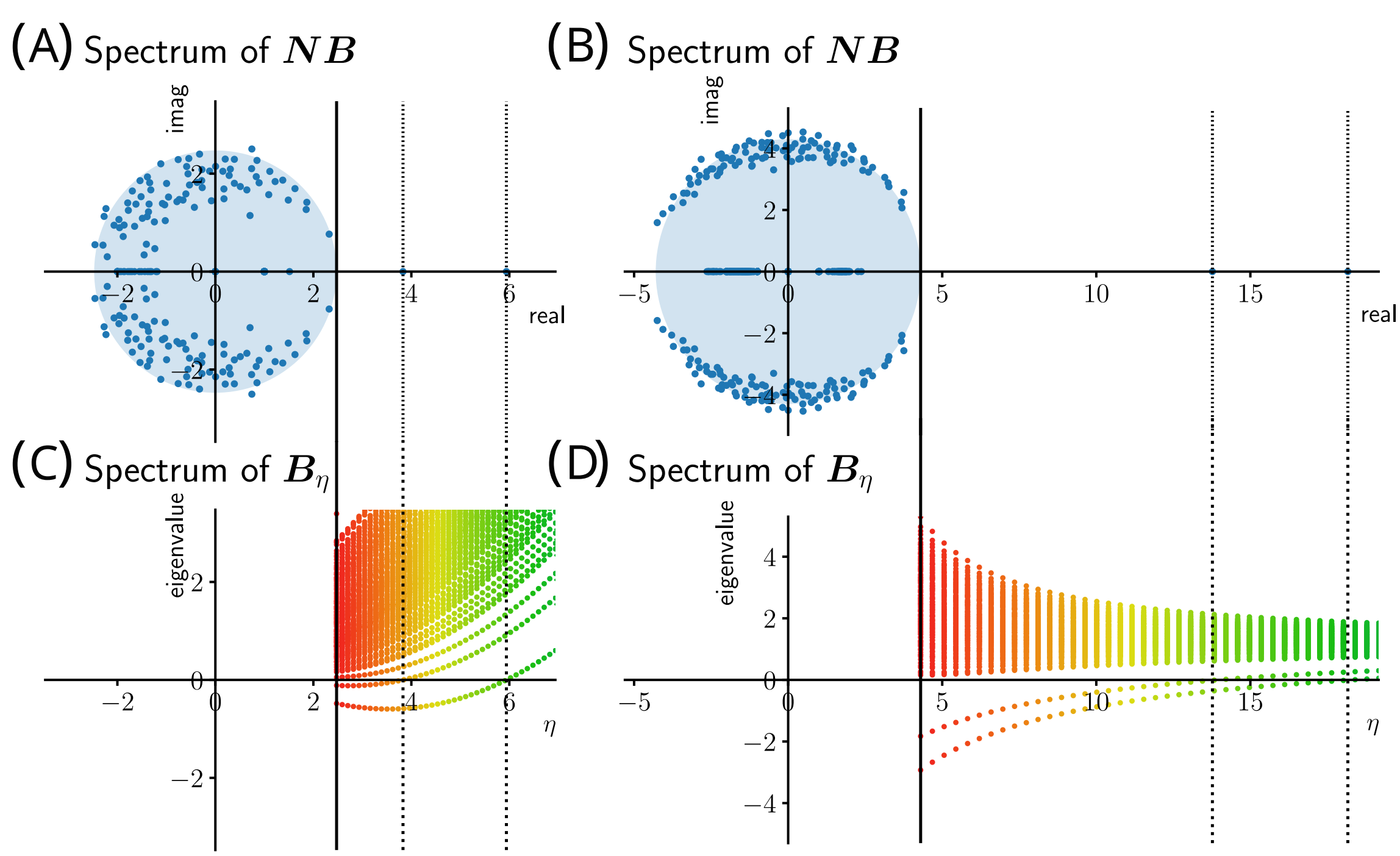

Figure S2: ***Spectra of non-backtracking and Bethe Hessian matrices in uniform and nonuniform HSBMs.*** *The spectrum of (**A**, **B**) non-backtracking matrix and (**C**, **D**) Bethe Hessian matrix for hypergraphs generated by $\kappa = 3$ uniform HSBM (**A**, **C**) and $\mathcal{K} = \{2, 3, 4\}$ nonuniform HSBM (**B**, **D**). In both settings, $n = 100$, $q = 2$ and degree parameters $d_{\text{in}} = 10$ and $d_{\text{out}} = 1$. The light blue circle is the uninformative bulk of $\boldsymbol{NB}$ with radius $\sum_{\kappa \in \mathcal{K}} \sqrt{d^{(\kappa)}(\kappa - 1)}$.*

S3 Time complexity comparison of spectral clustering operators

The computational cost of spectral clustering is primarily dominated by the eigen decomposition of the associated operator. For instance, when employing the Lanczos algorithm for eigen-decomposition (42), the time complexity is approximately $O(k(n+nnz))$, where $k \ll n$ is the number of Lanczos iterations, n is the size of the matrix and nnz is the number of non-zero entries. Crucially, the Lanczos algorithm operates more efficiently on symmetric matrices, giving the Bethe Hessian matrix a distinct advantage over the $\boldsymbol{NB}$.

Let us first consider the dyadic network case. For a network with n nodes and m edges, the dimension of its non-backtracking matrix $\boldsymbol{NB}$ is $n^*_{\boldsymbol{NB}} = 2m$ and the number of non-zero entries is $nnz_{\boldsymbol{NB}} = \sum_{i\in\mathcal{V}} d_i(d_i-1)$. For the Bethe Hessian matrix $\boldsymbol{B}$, the dimension is $n^*_{\boldsymbol{B}} = n$, and the number of non-zero entries is $nnz_{\boldsymbol{B}} = m+n$. Therefore, to compare the time complexity of $\boldsymbol{NB}$ based and $\boldsymbol{B}$ based spectral clustering, we compute

$$
\begin{aligned}
n^*_{\boldsymbol{NB}} + nnz_{\boldsymbol{NB}} - n^*_{\boldsymbol{B}} - nnz_{\boldsymbol{B}} &= 2m + \sum_i d_i(d_i-1) - n - n - m \\
&= \sum_i d_i(d_i-1) + m - 2n \\
&= \sum_i d_i^2 - 2m + m - 2n \\
&= \sum_i d_i^2 - m - 2n \\
&\geq nd^2 - nd/2 - 2n \\
&= n(d^2 - d/2 - 2) \ , \qquad (54)
\end{aligned}
$$

where d is the average degree. In a large connected network, $d > 2$, which makes (54) strictly greater than zero. Thus, the Bethe Hessian based spectral clustering has lower time complexity than the one based on non-backtracking matrix.

Now, consider a hypergraph containing hyperedges of all possible orders $\kappa \in \mathcal{K}$. Let $d_i^{(\kappa)}$ denote the order κ degree of node i. From the definition of the non-backtracking matrix (38), its dimension is equal to the total number of directed hyperedges:

$$
n^*_{\boldsymbol{NB}} = \sum_i \sum_\kappa d_i^{(\kappa)} \ , \qquad (55)
$$

and the number of non-zero entries is

$$
nnz_{\boldsymbol{NB}} = \sum_i \sum_\kappa d_i^{(\kappa)}(\kappa-1)(d_i-1) \ , \qquad (56)
$$

where the total degree of node i is $d_i = \sum_{\kappa\in\mathcal{K}} d_i^{(\kappa)}$. Based on the Hypergraph Bethe Hessian matrix (2), the dimension is $n^*_{\boldsymbol{B}} = n$. The number of non-zero entries in $\boldsymbol{B}$ has

the form $nnz_{\boldsymbol{B}} = n + x$, where x is the number of non-zero entries in its off-diagonal part. Each order κ hyperedge contributes $\binom{\kappa}{2}$ non-zero entries in $\boldsymbol{B}$, but different hyperedges may overlap in their contributions. Therefore we can upper bound x as,

$$x < \sum_{\kappa} m^{(\kappa)} \binom{\kappa}{2} = \sum_{\kappa} \sum_{i} \frac{d_i^{(\kappa)}}{\kappa} \frac{\kappa(\kappa-1)}{2} = \sum_{i} \sum_{\kappa} d_i^{(\kappa)} \frac{(\kappa-1)}{2} \quad . \tag{57}$$

We then have:

$$\begin{aligned} & n^*_{\boldsymbol{NB}} + nnz_{\boldsymbol{NB}} - n^*_{\boldsymbol{B}} - nnz_{\boldsymbol{B}} \\ > & \sum_{i} \sum_{\kappa} d_i^{(\kappa)} + \sum_{i} \sum_{\kappa} d_i^{(\kappa)} (\kappa-1)(d_i - 1) - n - \sum_{i} \sum_{\kappa} d_i^{(\kappa)} \frac{(\kappa-1)}{2} \\ = & \sum_{i} \sum_{\kappa} \left[d_i^{(\kappa)} (1 + (\kappa-1)(d_i - \frac{3}{2})) - \frac{1}{|\mathcal{K}|} \right] \quad . \end{aligned} \tag{58}$$

For a large sparse hypergraph, $d_i^{(\kappa)} \sim O(n)$. Thus, as $n \to \infty$, the above expression remains positive, leading to the same conclusion: for both dyadic networks and hypergraphs, the eigen decomposition of the Bethe Hessian has lower time complexity than the non-backtracking operator. This is also illustrated in Figure S3.

S4 Generalizing the spectral detectability limit of symmetric HSBM

To introduce the detectability limit of symmetric HSBM in literature (16, 17), we need to introduce the definition appeared in both of them:

$$\widehat{c}_a^{(\kappa)} = \frac{1}{(\kappa-1)!} \sum_{\left\{\vec{z} \mid \vec{z}_0 = a, \vec{z}_{1:} \in [q]^{\kappa-1}\right\}} \boldsymbol{C}_{\vec{z}}^{(\kappa)} \prod_{l=1}^{\kappa-1} p_{\vec{z}_l} \ , \tag{59}$$

and

$$\widehat{c}_{ab}^{(\kappa)} = \frac{1}{(\kappa-2)!} \sum_{\left\{\vec{z} \mid \vec{z}_0 = a, \vec{z}_1 = b, \vec{z}_{2:} \in [q]^{\kappa-2}\right\}} \boldsymbol{C}_{\vec{z}}^{(\kappa)} \prod_{l=2}^{\kappa-1} p_{\vec{z}_l} \ , \tag{60}$$

where $[q]^\kappa$ represent the cartesian product for κ sets $[q] = \{1, 2, ..., q\}$, p_r is the normalized community size of community r. In the case that all communities have the same size, $p_r = \frac{1}{q}$ and we can replace the product with the factor $\frac{1}{q^{\kappa-1}}$ in (59) and with $\frac{1}{q^{\kappa-2}}$ in (60). The relationship between $\widehat{c}_a^{(\kappa)}$ and $\widehat{c}_{ab}^{(\kappa)}$ is

$$\widehat{c}_a^{(\kappa)} = \frac{1}{\kappa-1} \sum_{b \in [q]} \widehat{c}_{ab}^{(\kappa)} n_b \tag{61}$$

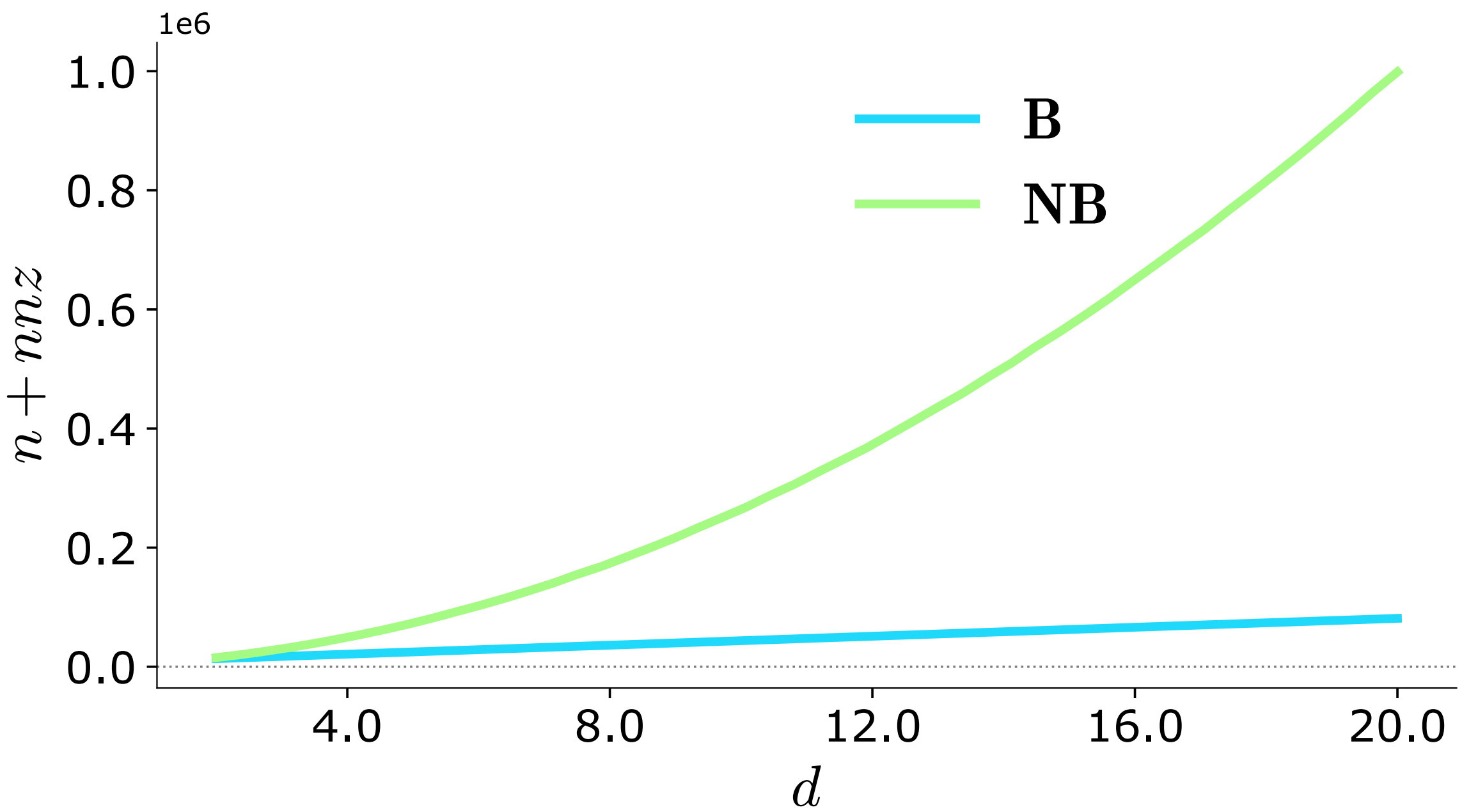

Figure S3: ***Time complexity of spectral clustering operators in hypergraphs.*** *The* $n+nnz$ *for operators* $\boldsymbol{B}$ *and* $\boldsymbol{NB}$ *which is a signal for the time complexity of spectral clustering. With different average degree, we sample the hypergraphs with* $n = 3000$, $q = 3$, $\kappa = (2, 3)$ *and* $\epsilon = 0.3$ *in detectable phase.*

Under the symmetric HSBM case, $p_r = \frac{1}{q}$ and $\boldsymbol{C}$ satisfy (6) with parameters c_{in} and c_{out}, a node can be incident on a maximum of $\binom{n/q-1}{\kappa-1}$ possible κ-hyperedges within the same community. Then we define the average κ-in-degree $d_{\text{in}}^{(\kappa)}$ can be calculated as:

$$\begin{aligned}
d_{\text{in}}^{(\kappa)} &= \lim_{n\to\infty} \binom{n/q-1}{\kappa-1} \frac{c_{\text{in}}}{n^{\kappa-1}} \\
&= \lim_{n\to\infty} \frac{c_{\text{in}}}{n^{\kappa-1}} \frac{(n/q-1)!}{(\kappa-1)!(n/q-\kappa)!} \\
&= \lim_{n\to\infty} \frac{c_{\text{in}}}{n^{\kappa-1}} \frac{(n/q-1)...(n/q-\kappa+1)}{(\kappa-1)!} \\
&= \frac{c_{\text{in}}}{n^{\kappa-1}} \frac{(n/q)^{\kappa-1}}{(\kappa-1)!} \\
&= \frac{c_{\text{in}}}{q^{\kappa-1}(\kappa-1)!} \quad .
\end{aligned} \tag{62}$$

A node can be connected to a maximum of $\binom{n-1}{\kappa-1} - \binom{n/q-1}{\kappa-1}$ possible κ-hyperedges that connect to at least one node in a different community. For each of the $q^{\kappa-1}-1$ ordered community combination of other $\kappa-1$ nodes, the out-community κ-degree of the node is

$$\begin{aligned}
d_{\text{out}}^{(\kappa)} &= \lim_{n\to\infty} \frac{\binom{n-1}{\kappa-1} - \binom{n/q-1}{\kappa-1}}{q^{\kappa-1}-1} \frac{c_{\text{out}}}{n^{\kappa-1}} \\
&= \frac{c_{\text{out}}}{n^{\kappa-1}} \frac{n^{\kappa-1}-(n/q)^{\kappa-1}}{(\kappa-1)!(q^{\kappa-1}-1)} \\
&= \frac{c_{\text{out}}}{q^{\kappa-1}(\kappa-1)!}
\end{aligned} \quad . \tag{63}$$

The average κ-degree for the node is

$$d^{(\kappa)} = d_{\text{in}}^{(\kappa)} + (q^{\kappa-1}-1)d_{\text{out}}^{(\kappa)} \quad . \tag{64}$$

With our definition of $d_{\text{in}}^{(\kappa)}$ and $d_{\text{out}}^{(\kappa)}$ above, we can represent the $\widehat{c}_{ab}^{(\kappa)}$ and $\widehat{c}_{a}^{(\kappa)}$ with notation $d_{\text{in}}^{(\kappa)}$ and $d_{\text{out}}^{(\kappa)}$:

$$\widehat{c}_{ab}^{(\kappa)} = \begin{cases} \widehat{c}_{\text{in}}^{(\kappa)} = \dfrac{c_{\text{in}} + (q^{\kappa-2}-1)c_{\text{out}}}{q^{\kappa-2}(\kappa-2)!} = q(\kappa-1)(d_{\text{in}}^{(\kappa)} + (q^{\kappa-2}-1)d_{\text{out}}^{(\kappa)}) & , a = b \\ \widehat{c}_{\text{out}}^{(\kappa)} = \dfrac{q^{\kappa-2}c_{\text{out}}}{q^{\kappa-2}(\kappa-2)!} = q(\kappa-1)q^{\kappa-2}d_{\text{out}}^{(\kappa)} & , a \neq b \end{cases} , \tag{65}$$

and with equation (61), we have

$$\begin{aligned}\widehat{c}_a^{(\kappa)} &= \frac{\widehat{c}_{\text{in}}^{(\kappa)} + (q-1)\widehat{c}_{\text{out}}^{(\kappa)}}{q(\kappa-1)} \\ &= \frac{\frac{c_{\text{in}}+(q^{\kappa-2}-1)c_{\text{out}}}{q^{\kappa-2}(\kappa-2)!} + (q-1)\frac{q^{\kappa-2}c_{\text{out}}}{q^{\kappa-2}(\kappa-2)!}}{q(\kappa-1)} \\ &= \frac{c_{\text{in}} + (q^{\kappa-1}-1)c_{\text{out}}}{q^{\kappa-1}(\kappa-1)!} \\ &= d_{\text{in}}^{(\kappa)} + (q^{\kappa-1}-1)d_{\text{out}}^{(\kappa)} \\ &= d^{(\kappa)}\end{aligned} \quad . \tag{66}$$

For the κ-uniform symmetric HSBM introduced in (17), the detectability threshold is

$$\begin{aligned}\frac{(\widehat{c}_{\text{in}}^{(\kappa)} - \widehat{c}_{\text{out}}^{(\kappa)})^2}{q^2 d^{(\kappa)}(\kappa-1)} &= 1 \\ \frac{\Big((\kappa-1)(d_{\text{in}}^{(\kappa)} - d_{\text{out}}^{(\kappa)})\Big)^2}{(\kappa-1)d^{(\kappa)}} &= 1\end{aligned} \quad . \tag{67}$$

which can degenerated from our format of detectability limit in (9) by restricting $\mathcal{K} = \{\kappa\}$.

For non-uniform symmetric HSBM with $q = 2$ considered in (16), they define:

$$\begin{aligned}\alpha_\kappa &= d^{(\kappa)}(\kappa-1) \\ \beta_\kappa &= \frac{\widehat{c}_{\text{in}}^{(\kappa)} - \widehat{c}_{\text{out}}^{(\kappa)}}{2}\end{aligned} \quad . \tag{68}$$

Then their conjectured detectability threshold of spectral clustering with non-backtracking matrix (16) (also for Bethe Hessian matrix since their relationship as shown in Section S2) is $\text{SNR}_{\text{NB}} = 1$ where

$$\begin{aligned}\text{SNR}_{\text{NB}} &:= \frac{(\sum_{\kappa\in\mathcal{K}} \beta_\kappa)^2}{\sum_{\kappa\in\mathcal{K}} \alpha_\kappa} \\ &= \frac{\Big(\sum_{\kappa\in\mathcal{K}} \widehat{c}_{\text{in}}^{(\kappa)} - \widehat{c}_{\text{out}}^{(\kappa)}\Big)^2}{2^2 \sum_{\kappa\in\mathcal{K}} d^{(\kappa)}(\kappa-1)} \\ &= \frac{\Big(\sum_{\kappa\in\mathcal{K}}(\kappa-1)(d_{\text{in}}^{(\kappa)} - d_{\text{out}}^{(\kappa)})\Big)^2}{\sum_{\kappa\in\mathcal{K}}(\kappa-1)d^{(\kappa)}}\end{aligned} \quad . \tag{69}$$

By simple generalizing the β_κ in (68) into

$$\beta_{\kappa,q} = \frac{\widehat{c}_{\text{in}}^{(\kappa)} - \widehat{c}_{\text{out}}^{(\kappa)}}{q} \quad , \tag{70}$$

we can get the spectral detectability limit of non-uniform symmetric HSBM for any q:

$$\begin{aligned}\mathrm{SNR}_{\mathrm{BH}} &:= \frac{(\sum_{\kappa\in\mathcal{K}}\beta_\kappa)^2}{\sum_{\kappa\in\mathcal{K}}\alpha_\kappa} \\ &= \frac{\left(\sum_{\kappa\in\mathcal{K}}\widehat{c}_{\mathrm{in}}^{(\kappa)}-\widehat{c}_{\mathrm{out}}^{(\kappa)}\right)^2}{q^2\sum_{\kappa\in\mathcal{K}}(\kappa-1)d^{(\kappa)}} \\ &= \frac{\left(\sum_{\kappa\in\mathcal{K}}(\kappa-1)(d_{\mathrm{in}}^{(\kappa)}-d_{\mathrm{out}}^{(\kappa)})\right)^2}{\sum_{\kappa\in\mathcal{K}}(\kappa-1)d^{(\kappa)}}\end{aligned} \quad . \tag{71}$$

S5 Belief propagation and detectability limit of symmetric HSBM

Similar with belief propagation in graph (6), the main process of belief propagation in hypergraph is to update the message $b_{\psi_i}^{i\to e}$ in all directed hyperedges, and then update the marginal probability $b_{\psi_i}^{i}$ that node i belong to community ψ_i for all nodes. Here $b_{\psi_i}^{i\to e}$ represent the marginal probability that node i belong to community ψ_i without hyperedge e. However, unlike graph, we need another intermediate hyperedge to node message $\hat{b}_{\psi_i}^{e\to i}$ when we update the node to hyperedge message $b_{\psi_i}^{i\to e}$. To make a distinction with all exist hyperedges $\mathcal{E}$, we use $\boldsymbol{E}$ represent all possible hyperedges. Then the belief propagation can be formalized as

$$\begin{aligned}\text{node to hyperedge message}: b_{\psi_i}^{i\to e} &\propto n_{\psi_i}\prod_{f\in\partial i/e\cap\boldsymbol{E}}\hat{b}_{\psi_i}^{f\to i} \\ \text{hyperedge to node message}: \hat{b}_{\psi_i}^{e\to i} &\propto \sum_{\overrightarrow{\psi_{e/i}}\in[q]^{|e|-1}}\left(\boldsymbol{\Omega}_{\overrightarrow{\psi_e}}\right)^{A_e}\left(1-\boldsymbol{\Omega}_{\overrightarrow{\psi_e}}\right)^{1-A_e}\prod_{j\in e/i}b_{\psi_j}^{j\to e} \\ \text{node marginal}: b_{\psi_i}^{i} &\propto n_{\psi_i}\prod_{f\in\partial i\cap\boldsymbol{E}}\hat{b}_{\psi_i}^{f\to i}\end{aligned} \quad , \tag{72}$$

where $A_e=1$ only if $e\in\mathcal{E}$, otherwise $A_e=0$. $\overrightarrow{\psi_{e/i}}$ means the community assignment for the nodes in hyperedge e except node i, n_{ψ_i} means number of nodes in community ψ_i by percentage. We illustrate $b^{i\to e}$, $\hat{b}^{e\to i}$ and b^i with factor graph (43) in Figure S4.

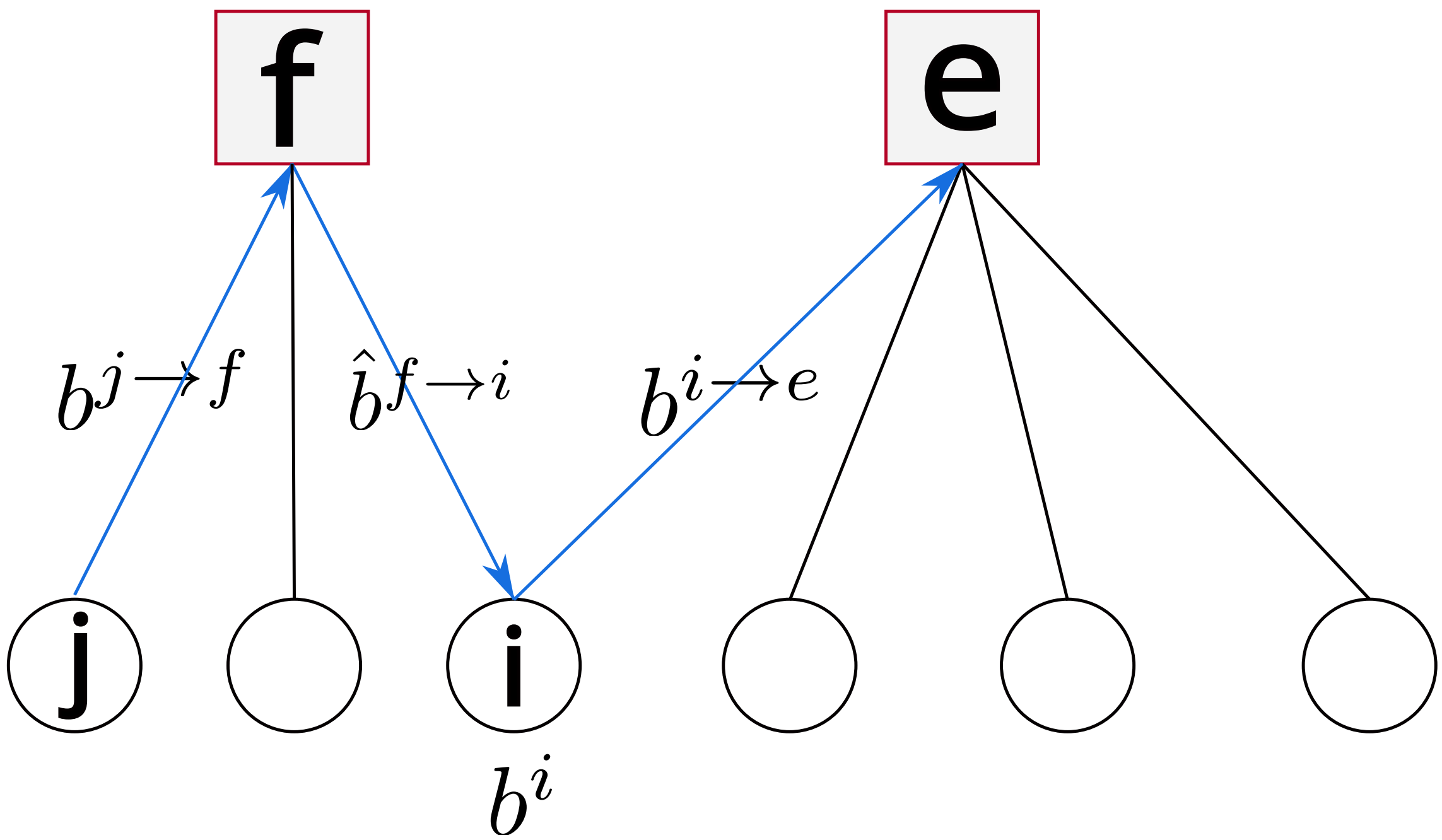

Figure S4: ***Mapping hypergraphs to factor graphs for message passing.*** *The red square represent the hyperedge, the circle represent the node. The message and marginal probability on directed hyperedge and node are also shown as $b^{j\to f}$, $b^{i\to e}$, $\hat{b}^{f\to i}$ and b^i. The blue directed hyperedge form a non-backtracking path $b^{j\to f} \to \hat{b}^{f\to i} \to b^{i\to e}$.*

Next we discuss the $\hat{b}^{e\to i}_{\psi_i}$ based on existence of hyperedge e.

S5.1 Discussion on hyperedge to node message

When hyperedge e exist, $A_e = 1$, we have

$$\begin{aligned} \hat{b}^{e\to i}_{\psi_i} &\propto \sum_{\overrightarrow{\psi_{e/i}} \in [q]^{|e|-1}} \boldsymbol{\Omega}_{\overrightarrow{\psi_e}} \prod_{j\in e/i} b^{j\to e}_{\psi_j} \\ &\propto \sum_{\overrightarrow{\psi_{e/i}} \in [q]^{|e|-1}} \boldsymbol{C}_{\overrightarrow{\psi_e}} \prod_{j\in e/i} b^{j\to e}_{\psi_j} \end{aligned} \quad . \tag{73}$$

However if $A_e = 0$, then

$$\begin{aligned}
\hat{b}^{e\to i}_{\psi_i} &\propto \sum_{\overrightarrow{\psi_{e/i}} \in [q]^{|e|-1}} \left(1 - \mathbf{\Omega}_{\overrightarrow{\psi_e}}\right) \prod_{j \in e/i} b^{j\to e}_{\psi_j} \\
&= \sum_{\overrightarrow{\psi_{e/i}} \in [q]^{|e|-1}} \left(1 - \frac{\boldsymbol{C}_{\overrightarrow{\psi_e}}}{n^{|e|-1}}\right) \prod_{j \in e/i} b^{j\to e}_{\psi_j} \\
&= \sum_{\overrightarrow{\psi_{e/i}} \in [q]^{|e|-1}} \prod_{j \in e/i} b^{j\to e}_{\psi_j} - \sum_{\overrightarrow{\psi_{e/i}} \in [q]^{|e|-1}} \frac{\boldsymbol{C}_{\overrightarrow{\psi_e}}}{n^{|e|-1}} \prod_{j \in e/i} b^{j\to e}_{\psi_j} \quad , \\
&= \prod_{j \in e/i} \sum_{\psi_j \in [q]} b^{j\to e}_{\psi_j} - \sum_{\overrightarrow{\psi_{e/i}} \in [q]^{|e|-1}} \frac{\boldsymbol{C}_{\overrightarrow{\psi_e}}}{n^{|e|-1}} \prod_{j \in e/i} b^{j\to e}_{\psi_j} \\
&= 1 - \sum_{\overrightarrow{\psi_{e/i}} \in [q]^{|e|-1}} \frac{\boldsymbol{C}_{\overrightarrow{\psi_e}}}{n^{|e|-1}} \prod_{j \in e/i} b^{j\to e}_{\psi_j}
\end{aligned} \tag{74}$$

where the last line use $\sum_{\psi_j \in [q]} b^{j\to e}_{\psi_j} = 1$. According to sparse assumption, $\boldsymbol{C}_{\overrightarrow{\psi_e}}$ is a constant compared to n, so

$$\lim_{n\to\infty} \hat{b}^{e\to i}_{\psi_i} = \frac{1}{q} \quad . \tag{75}$$

According to (72), we have

$$\begin{aligned}
b^{j\to e}_{\psi_j} &\propto n_{\psi_j} \prod_{f \in \partial j/e} \hat{b}^{f\to j}_{\psi_j} \\
&= \frac{n_{\psi_j}}{\hat{b}^{e\to j}_{\psi_j}} \prod_{f \in \partial j} \hat{b}^{f\to j}_{\psi_j} \quad . \\
&= \frac{b^{j}_{\psi_j}}{\hat{b}^{e\to j}_{\psi_j}}
\end{aligned} \tag{76}$$

Based on (75) and (76), we have

$$\lim_{n\to\infty} b^{j\to e}_{\psi_j} = b^{j}_{\psi_j} \quad . \tag{77}$$

We substitute (77) into (74) and consider $1 + x \approx \exp(x)$ when $x \to 0$, then when $n \to \infty$

$$\hat{b}^{e\to i}_{\psi_i} = \exp\left(-\frac{1}{n^{|e|-1}} \sum_{\overrightarrow{\psi_{e/i}} \in [q]^{|e|-1}} \boldsymbol{C}_{\overrightarrow{\psi_e}} \prod_{j \in e/i} b^{j}_{\psi_j}\right) \quad . \tag{78}$$

S5.2 Update of node to hyperedge message and external field

Based on $\hat{b}_{\psi_i}^{e\to i}$ in different case (73) and (78), we can update node to hyperedge message as

$$\begin{aligned} b_{\psi_i}^{i\to e} &\propto n_{\psi_i} \prod_{f\in\partial i/e\cap\boldsymbol{E}} \hat{b}_{\psi_i}^{f\to i} \\ &= n_{\psi_i} \prod_{f\in\partial i/e\cap\mathcal{E}} \hat{b}_{\psi_i}^{f\to i} \prod_{f\in\partial i/e\cap\boldsymbol{E}/\mathcal{E}} \hat{b}_{\psi_i}^{f\to i} \\ &= n_{\psi_i} \prod_{f\in\partial i/e\cap\mathcal{E}} \hat{b}_{\psi_i}^{f\to i} \prod_{f\in\partial i/e\cap\boldsymbol{E}/\mathcal{E}} \exp\left(-\frac{1}{n^{|f|-1}} \sum_{\overrightarrow{\psi_{f/i}}\in[q]^{|f|-1}} \boldsymbol{C}_{\overrightarrow{\psi_f}} \prod_{j\in f/i} b_{\psi_j}^{j}\right) . \\ &= n_{\psi_i} \prod_{f\in\partial i/e\cap\mathcal{E}} \hat{b}_{\psi_i}^{f\to i} \exp\left(-\sum_{f\in\partial i/e\cap\boldsymbol{E}/\mathcal{E}} \frac{1}{n^{|f|-1}} \sum_{\overrightarrow{\psi_{f/i}}\in[q]^{|f|-1}} \boldsymbol{C}_{\overrightarrow{\psi_f}} \prod_{j\in f/i} b_{\psi_j}^{j}\right) \\ &= n_{\psi_i} \prod_{f\in\partial i/e\cap\mathcal{E}} \hat{b}_{\psi_i}^{f\to i} \exp\left(-h_{\psi_i}^{i\to e}\right) \end{aligned} \tag{79}$$

Because of $e \in \mathcal{E}$ and the existed hyperedge set $\mathcal{E}$ is far smaller than all possible hyperedge set $\boldsymbol{E}$ in sparse regime, so

$$h_{\psi_i}^{i\to e} = h_{\psi_i}^{i} = \sum_{f\in\partial i\cap\boldsymbol{E}} \frac{1}{n^{|f|-1}} \sum_{\overrightarrow{\psi_{f/i}}\in[q]^{|f|-1}} \boldsymbol{C}_{\overrightarrow{\psi_f}} \prod_{j\in f/i} b_{\psi_j}^{j} . \tag{80}$$

We call the $h_{\psi_i}^{i}$ as external field. The external field can not be reduced anymore for general tensor $\boldsymbol{C}$. However, considering the specific form of $\boldsymbol{C}$ (6) in symmetric HSBM,

we can reduce $h^i_{\psi_i}$ to h_{ψ_i}

$$
\begin{aligned}
h^i_{\psi_i} &= \sum_{f\in\partial i\cap \boldsymbol{E}} \frac{1}{n^{|f|-1}} \left(c_{\text{in}} \prod_{j\in f/i} b^j_{\psi_i} + c_{\text{out}} \sum_{\overrightarrow{\psi_{f/i}}\in[q]^{|f|-1}/\underbrace{[\psi_i]}_{|f|-1}} \prod_{j\in f/i} b^j_{\psi_j} \right) \\
&= \sum_{\kappa\in\mathcal{K}} \frac{1}{n^{\kappa-1}} \sum_{f\in\boldsymbol{E}^{(\kappa-1)}} \left(c_{\text{in}} \prod_{j\in f} b^j_{\psi_i} + c_{\text{out}} \sum_{\overrightarrow{\psi_f}\in[q]^{\kappa-1}/\underbrace{[\psi_i]}_{\kappa-1}} \prod_{j\in f} b^j_{\psi_j} \right) \quad , \\
&= \sum_{\kappa\in\mathcal{K}} \frac{1}{n^{\kappa-1}} \sum_{f\in\boldsymbol{E}^{(\kappa-1)}} \left(c_{\text{out}} + (c_{\text{in}} - c_{\text{out}}) \prod_{j\in f} b^j_{\psi_i} \right) \\
&\approx \sum_{\kappa\in\mathcal{K}} \left[c_{\text{out}} + \frac{c_{\text{in}} - c_{\text{out}}}{n^{\kappa-1}(\kappa-1)!} (\sum_{j\in[n]} b^j_{\psi_i})^{\kappa-1} \right] = h_{\psi_i}
\end{aligned}
\tag{81}
$$

where the second last line is derived based on $\sum_{\overrightarrow{\psi_f}\in[q]^{\kappa-1}} \prod_{j\in f} b^j_{\psi_j} = 1$. The last line is derived with similar idea in paper (17).

S5.3 Update of node marginal and summary

The node marginal $b^i_{\psi_i}$ is updated similarly to $b^{i\to e}_{\psi_i}$,

$$
\begin{aligned}
b^i_{\psi_i} &\propto n_{\psi_i} \prod_{f\in\partial i} \hat{b}^{f\to i}_{\psi_i} \\
&= n_{\psi_i} \prod_{f\in\partial i\cap\mathcal{E}} \hat{b}^{f\to i}_{\psi_i} \prod_{f\in\partial i\cap \boldsymbol{E}/\mathcal{E}} \hat{b}^{f\to i}_{\psi_i} \quad . \\
&= n_{\psi_i} \exp\left(-h^i_{\psi_i}\right) \prod_{f\in\partial i\cap\mathcal{E}} \hat{b}^{f\to i}_{\psi_i}
\end{aligned}
\tag{82}
$$

Based on above derivation, we can summary the belief propagation as

$$
\begin{aligned}
\text{node to hyperedge message}: b_{\psi_i}^{i\to e} &\propto n_{\psi_i}\exp\left(-h_{\psi_i}^i\right)\prod_{f\in\partial i/e\cap\mathcal{E}}\hat{b}_{\psi_i}^{f\to i}\\
\text{hyperedge to node message}: \hat{b}_{\psi_i}^{e\to i} &\propto \sum_{\overrightarrow{\psi_{e/i}}\in[q]^{|e|-1}}\boldsymbol{C}_{\overrightarrow{\psi_e}}\prod_{j\in e/i}b_{\psi_j}^{j\to e}\\
\text{external field}: h_{\psi_i}^i &= \sum_{f\in\partial i\cap\boldsymbol{E}}\frac{1}{n^{|f|-1}}\sum_{\overrightarrow{\psi_{f/i}}\in[q]^{|f|-1}}\boldsymbol{C}_{\overrightarrow{\psi_f}}\prod_{j\in f/i}b_{\psi_j}^{j}\\
\text{node marginal}: b_{\psi_i}^{i} &\propto n_{\psi_i}\exp\left(-h_{\psi_i}^i\right)\prod_{f\in\partial i\cap\mathcal{E}}\hat{b}_{\psi_i}^{f\to i}
\end{aligned}
\tag{83}
$$

In practice, we apply dropout on the messages with same setting in Ruggeri et al. (18).

S5.4 The detectability of belief propagation

Similarly with paper (6) and (18), we get the detectability by deriving the stability of trivial fixed point in belief propagation. We will focus on symmetric HSBM case (6). In this case, $n_{\psi_i} = \frac{1}{q}$ and we can verify that there is a trivial fixed point of belief propagation based on (83) and external field (81)

$$
\begin{aligned}
b_{\psi_i}^{i\to e} &= \frac{1}{q}\\
\hat{b}_{\psi_i}^{e\to i} &= \frac{1}{q}\\
b_{\psi_i}^{i} &= \frac{1}{q}
\end{aligned}
\quad . \tag{84}
$$

This trivial fixed point means nothing but any node belong to any community with same probability, so we need this trivial fixed point should be unstable. One way to discuss the stability of the fixed point is to get the largest modular eigenvalue of Jacobian Matrix of belief propagation (40). Here we use another approach which is inherited from (6) and (18).

We assume the message passing as a tree with depth s, as shown in Figure S5. We will check if the node to hyperedge message perturbation around trivial fixed point will be amplified or reduced during its propagation from all the leaves to root. First, we need to know how the perturbation propagate between adjacent node to hyperedge message, such as $b^{i_s\to f_s}$ and $b^{i_{s-1}\to f_{s-1}}$ in Figure S5. This propagation progress can be formalized as transition matrix $\hat{\mathrm{T}}$ with dimension $q\times q$, defined as

$$
\hat{\mathrm{T}}_{x;y}^{i\to e;j\to f} = \frac{\partial b_x^{i\to e}}{\partial b_y^{j\to f}} \quad , \tag{85}
$$

where $e \neq f$ and $i \in f/j$, as this short message passing path is $b^{j\to f} \to \hat{b}^{f\to i} \to b^{i\to e}$. Next we will derive this transition matrix at trivial fixed point.

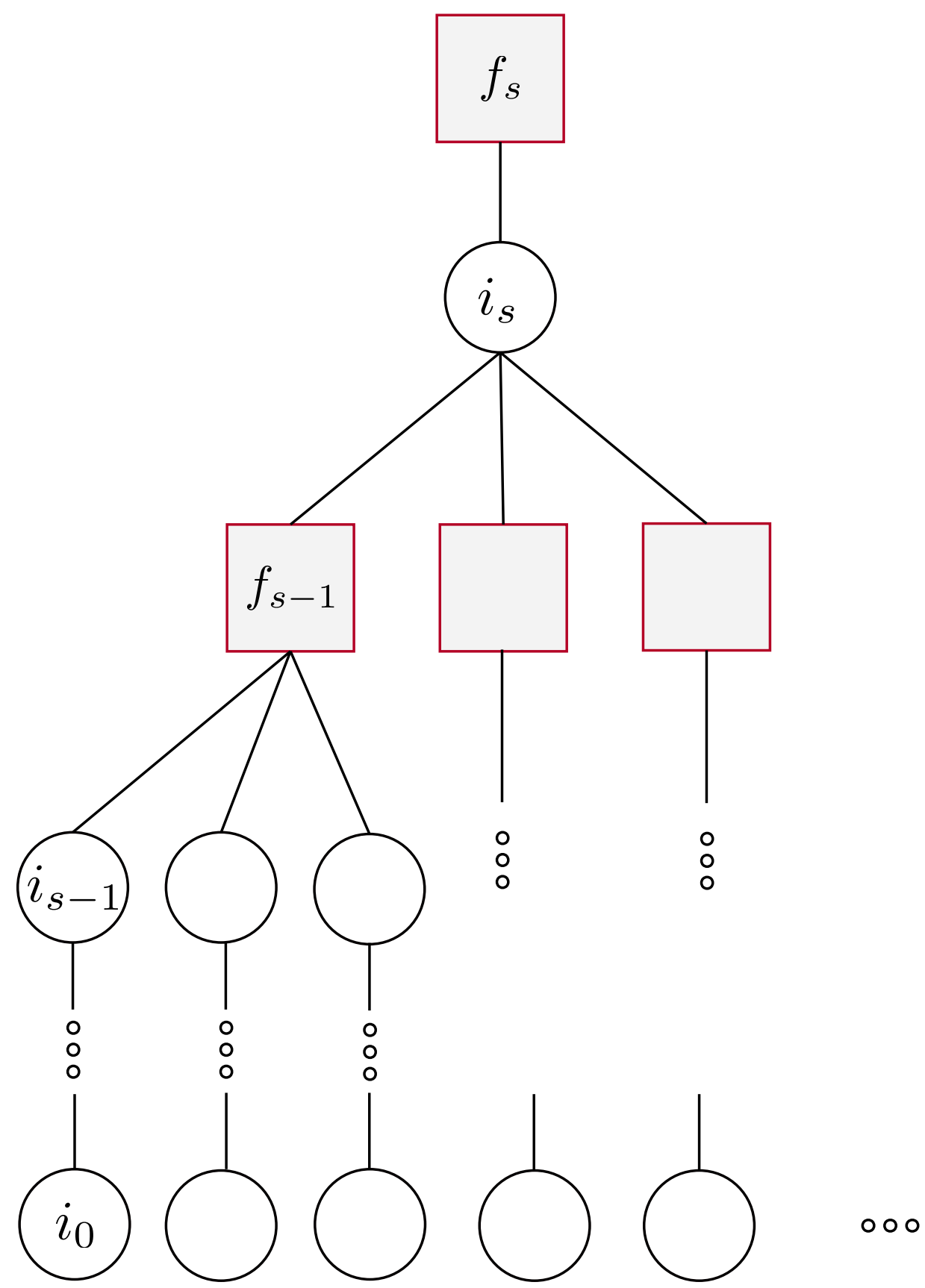

Figure S5: ***The approximate message passing tree of belief propagation on a hypergraph.***

S5.4.1 Transition matrix at trivial fixed point

Based on belief propagation (83), we can write the full update function of $b_x^{i\to e}$ without hyperedge to node message as

$$b_x^{i\to e} = \frac{1}{Z^{i\to e}} n_x \exp(-h_x^i) \prod_{g\in\partial i/e\cap\mathcal{E}} \sum_{\overrightarrow{\psi_{g/i}}\in[q]^{|g|-1}, \overrightarrow{\psi_i}=x} \boldsymbol{C}_{\overrightarrow{\psi_g}} \prod_{l\in g/i} b_{\psi_l}^{j\to g} \ , \tag{86}$$

where $Z^{i\to e}$ is normalization term over community label x. We define the numerator part of (86) as $\beta_x^{i\to e}$, then the transition matrix can be transformed as

$$
\begin{aligned}
\hat{\mathrm{T}}_{x;y}^{i\to e;j\to f} &= \frac{\partial b_x^{i\to e}}{\partial b_y^{j\to f}} \\
&= \frac{1}{Z^{i\to e}}\frac{\partial \beta_x^{i\to e}}{\partial b_y^{j\to f}} - \frac{1}{(Z^{i\to e})^2}\frac{\partial Z^{i\to e}}{\partial b_y^{j\to f}}\beta_x^{i\to e}
\end{aligned} \quad . \tag{87}
$$

In symmetric HSBM case, the external field $h_{\psi_i}^i$ is a constant at trivial fixed point based on (81)

$$
\begin{aligned}
h_{\psi_i}^i = h_{\psi_i} &= \sum_{\kappa\in\mathcal{K}}\left[c_{\text{out}} + \frac{c_{\text{in}} - c_{\text{out}}}{n^{\kappa-1}(\kappa-1)!}(\sum_{j\in[n]}\frac{1}{q})^{\kappa-1}\right] \\
&= \sum_{\kappa\in\mathcal{K}}\left[c_{\text{out}} + \frac{c_{\text{in}} - c_{\text{out}}}{q^{\kappa-1}(\kappa-1)!}\right] = h
\end{aligned} \quad . \tag{88}
$$

The average κ-order degree (66) is also a constant. Based on (59), we have

$$
\sum_{\overrightarrow{\psi_{g/i}}\in[q]^{|g|-1},\overrightarrow{\psi_i}=x} \boldsymbol{C}_{\overrightarrow{\psi_g}}\prod_{l\in g/i}\frac{1}{q} = d^{(|g|)}(|g|-1)! \quad . \tag{89}
$$

Then at trivial fixed point, $\beta_x^{i\to e}$ is

$$
\begin{aligned}
\beta_x^{i\to e} &= n_x \exp(-h_x^i)\prod_{g\in\partial i/e\cap\mathcal{E}}\ \sum_{\overrightarrow{\psi_{g/i}}\in[q]^{|g|-1},\overrightarrow{\psi_i}=x}\boldsymbol{C}_{\overrightarrow{\psi_g}}\prod_{l\in g/i}\frac{1}{q} \\
&= \frac{\exp(-h)}{q}\prod_{g\in\partial i/e\cap\mathcal{E}} d^{(|g|)}(|g|-1)!
\end{aligned} \quad , \tag{90}
$$

and $Z^{i\to e}$ is

$$
\begin{aligned}
Z^{i\to e} &= \sum_{x\in[q]}\beta_x^{i\to e} \\
&= \sum_{x\in[q]}\frac{\exp(-h)}{q}\prod_{g\in\partial i/e\cap\mathcal{E}} d^{(|g|)}(|g|-1)! \\
&= \exp(-h)\prod_{g\in\partial i/e\cap\mathcal{E}} d^{(|g|)}(|g|-1)!
\end{aligned} \quad . \tag{91}
$$

Next the partial derivative $\frac{\partial \beta_x^{i\to e}}{\partial b_y^{j\to f}}$ is

$$\begin{aligned}
\frac{\partial \beta_x^{i\to e}}{\partial b_y^{j\to f}} =& \frac{\partial \left\{ n_x \exp(-h_x^i) \prod_{g\in\partial i/e\cap\mathcal{E}} \sum_{\overrightarrow{\psi_{g/i}}\in[q]^{|g|-1},\overrightarrow{\psi_i}=x} \boldsymbol{C}_{\overrightarrow{\psi_g}} \prod_{l\in g/i} b_{\psi_l}^{l\to g} \right\}}{\partial b_y^{j\to f}} \\
=& \frac{\exp(-h)}{q} \left(\prod_{g\in\partial i/e,f\cap\mathcal{E}} \sum_{\overrightarrow{\psi_{g/i}}\in[q]^{|g|-1},\overrightarrow{\psi_i}=x} \boldsymbol{C}_{\overrightarrow{\psi_g}} \prod_{l\in g/i} b_{\psi_l}^{l\to g} \right) \\
& \times \sum_{\overrightarrow{\psi_{f/i,j}}\in[q]^{|f|-2},\overrightarrow{\psi_i}=x,\overrightarrow{\psi_j}=y} \boldsymbol{C}_{\overrightarrow{\psi_f}} \prod_{l\in f/i,j} b_{\psi_l}^{l\to f} \\
=& \frac{\exp(-h)}{q} \left(\prod_{g\in\partial i/e,f\cap\mathcal{E}} d^{(|g|)}(|g|-1)! \right) \sum_{\overrightarrow{\psi_{f/i,j}}\in[q]^{|f|-2},\overrightarrow{\psi_i}=x,\overrightarrow{\psi_j}=y} \boldsymbol{C}_{\overrightarrow{\psi_f}} \prod_{l\in f/i,j} \frac{1}{q} \\
=& \frac{\exp(-h)}{q} \left(\prod_{g\in\partial i/e,f\cap\mathcal{E}} d^{(|g|)}(|g|-1)! \right) \hat{c}_{xy}^{(|f|)}(|f|-2)!
\end{aligned}, \quad (92)$$

where the last line is based on definition (60). In symmetric HSBM case, $\hat{c}_{xy}^{(|f|)}$ is follow (65), so we have

$$\frac{\partial \beta_x^{i\to e}}{\partial b_y^{j\to f}} = \frac{\exp(-h)}{q} \left(\prod_{g\in\partial i/e,f\cap\mathcal{E}} d^{(|g|)}(|g|-1)! \right) \frac{c_{\text{in}}\delta_{x,y} + c_{\text{out}}(1-\delta_{x,y}) + (q^{|f|-2}-1)c_{\text{out}}}{q^{|f|-2}}, \quad (93)$$

where $\delta_{x,y} = 1$ if $x = y$. Then we have

$$\begin{aligned}
\frac{\partial Z^{i\to e}}{\partial b_y^{j\to f}} &= \sum_{x\in[q]} \frac{\partial \beta_x^{i\to e}}{\partial b_y^{j\to f}} \\
&= \exp(-h) \left(\prod_{g\in\partial i/e,f\cap\mathcal{E}} d^{(|g|)}(|g|-1)! \right) \left(\frac{c_{\text{in}} + (q^{|f|-2}-1)c_{\text{out}}}{q^{|f|-1}} + \frac{c_{\text{out}}}{q}(q-1) \right) \\
&= \exp(-h) \left(\prod_{g\in\partial i/e,f\cap\mathcal{E}} d^{(|g|)}(|g|-1)! \right) \left(\frac{c_{\text{in}} + (q^{|f|-1}-1)c_{\text{out}}}{q^{|f|-1}} \right)
\end{aligned} \quad (94)$$

Finally substitute (90), (91), (93), (94) into (87), we get the transition matrix at trivial fixed point

$$
\begin{aligned}
\hat{\mathrm{T}}_{x;y}^{i\to e;j\to f} =& \frac{1}{Z^{i\to e}}\frac{\partial \beta_x^{i\to e}}{\partial b_y^{j\to f}} - \frac{1}{(Z^{i\to e})^2}\frac{\partial Z^{i\to e}}{\partial b_y^{j\to f}}\beta_x^{i\to e} \\
=& \frac{\frac{\exp(-h)}{q}\left(\prod_{g\in\partial i/e, f\cap\mathcal{E}} d^{(|g|)}(|g|-1)!\right)\frac{c_{\mathrm{in}}\delta_{x,y}+c_{\mathrm{out}}(1-\delta_{x,y})+(q^{|f|-2}-1)c_{\mathrm{out}}}{q^{|f|-2}}}{\exp(-h)\prod_{g\in\partial i/e\cap\mathcal{E}} d^{(|g|)}(|g|-1)!} \\
&- \frac{\exp(-h)\left(\prod_{g\in\partial i/e, f\cap\mathcal{E}} d^{(|g|)}(|g|-1)!\right)\left(\frac{c_{\mathrm{in}}+(q^{|f|-1}-1)c_{\mathrm{out}}}{q^{|f|-1}}\right)}{q\exp(-h)\prod_{g\in\partial i/e\cap\mathcal{E}} d^{(|g|)}(|g|-1)!} \\
=& \frac{1}{qd^{(|f|)}(|f|-1)!}\frac{c_{\mathrm{in}}\delta_{x,y}+c_{\mathrm{out}}(1-\delta_{x,y})+(q^{|f|-2}-1)c_{\mathrm{out}}}{q^{|f|-2}} \\
&- \frac{1}{qd^{(|f|)}(|f|-1)!}\frac{c_{\mathrm{in}}+(q^{|f|-1}-1)c_{\mathrm{out}}}{q^{|f|-1}} \\
=& \frac{1}{d^{(|f|)}(|f|-1)!}\frac{qc_{\mathrm{in}}\delta_{x,y}+qc_{\mathrm{out}}(1-\delta_{x,y})+q(q^{|f|-2}-1)c_{\mathrm{out}}-c_{\mathrm{in}}-(q^{|f|-1}-1)c_{\mathrm{out}}}{q^{|f|}} \\
=& \frac{1}{d^{(|f|)}(|f|-1)!}\frac{c_{\mathrm{in}}(q\delta_{x,y}-1)+c_{\mathrm{out}}(1-q\delta_{x,y})}{q^{|f|}} \\
=& \frac{1}{d^{(|f|)}(|f|-1)!}\frac{(q\delta_{x,y}-1)(c_{\mathrm{in}}-c_{\mathrm{out}})}{q^{|f|}}
\end{aligned} \quad . \tag{95}
$$

We define J is all ones matrix, then the transition matrix $\hat{\mathrm{T}}$ is

$$
\begin{aligned}
\hat{\mathrm{T}}^{i\to e;j\to f} &= \frac{1}{d^{(|f|)}(|f|-1)!q^{|f|}}\left[q(c_{\mathrm{in}}-c_{\mathrm{out}})\mathrm{I}+(c_{\mathrm{out}}-c_{\mathrm{in}})\mathrm{J}\right] \\
&= \frac{1}{d^{(|f|)}(|f|-1)!q^{|f|}}\mathrm{T}
\end{aligned} \quad . \tag{96}
$$

S5.4.2 Stability of trivial fixed point

We first focus on the message passing path in Figure S5 from i_0 to f_s. We define ε^r as the perturbation of message $b^{i_r\to f_r}$ around trivial fixed point, as $b^{i_r\to f_r} = \frac{1}{q}+\varepsilon^r$. Then we have

$$
\begin{aligned}
\varepsilon^{r+1} &= \hat{\mathrm{T}}^{i_{r+1}\to f_{r+1};i_r\to f_r}\varepsilon^r \\
&= \frac{1}{d^{(|f_r|)}(|f_r|-1)!q^{|f_r|}}\mathrm{T}\varepsilon^r
\end{aligned} \quad . \tag{97}
$$

The perturbation ε^0 in the leaf will propagate s times to the ε^s, so

$$
\varepsilon^s = \left(\prod_{r=0}^{s-1}\frac{1}{d^{(|f_r|)}(|f_r|-1)!q^{|f_r|}}\right)\mathrm{T}^s\varepsilon^0 \quad . \tag{98}
$$

When $s \to \infty$, we define

$$\begin{aligned}\mu &= \lim_{s\to\infty} \prod_{r=0}^{s-1} \frac{1}{d^{(|f_r|)}(|f_r|-1)!q^{|f_r|}} \\ &= \exp\left(\lim_{s\to\infty}\sum_{r=0}^{s-1} \log \frac{1}{d^{(|f_r|)}(|f_r|-1)!q^{|f_r|}}\right) , \\ &\approx \exp\left(s\mathbb{E}_f\left(\log\frac{1}{d^{(|f|)}(|f|-1)!q^{|f|}}\right)\right)\end{aligned} \tag{99}$$

where $\mathbb{E}_f$ is the expectation over randomly drawn hyperedges $f \in \mathcal{E}$. On the other hand, the leading eigenvalue of T is $\lambda = q(c_{\text{in}} - c_{\text{out}})$. So the equation (98) is approximated as

$$\varepsilon^s = \mu\lambda^s\varepsilon^0 \ . \tag{100}$$

Before proceeding with the following derivation, we observe that the perturbation of node marginal b^i follow the same transition as perturbation of $b^{i\to e}$. That is equal to say the $\hat{\mathrm{T}}^{i;j\to f} = \hat{\mathrm{T}}^{i\to e;j\to f}$, where

$$\hat{\mathrm{T}}^{i;j\to f}_{x;y} = \frac{\partial b^i_x}{\partial b^{j\to f}_y} \ . \tag{101}$$

This can be derived with similar way in section S5.4.1, just remove e in each equation. So now, we can define ε^r also to be the perturbation of b^{i_r} except $r = 0$, and the equation (100) still holds. However in this case, each node can average has d hyperedges as its son nodes in message passing tree, each hyperedge still average has $\hat{\kappa} - 1$ nodes as its son nodes in message passing tree. Then there are totally $[d(\hat{\kappa}-1)]^s$ leave nodes whose perturbation will finally affect ε^s in root node i_s. Here, the $\hat{\kappa}$ is the average order of hyperedges. To derive $\hat{\kappa}$, we first estimate the expected number of order-κ hyperedges $m^{(\kappa)}$ under the symmetric HSBM. As $n \to \infty$, this estimation yields:

$$\begin{aligned}m^{(\kappa)} &= \lim_{n\to\infty} q\binom{\frac{n}{q}}{\kappa}\frac{c_{\text{in}}}{n^{\kappa-1}} + (\binom{n}{\kappa} - q\binom{\frac{n}{q}}{\kappa})\frac{c_{\text{out}}}{n^{\kappa-1}} \\ &= \frac{n}{\kappa!q^{\kappa-1}}c_{\text{in}} + \frac{n(q^{\kappa-1}-1)}{\kappa!q^{\kappa-1}}c_{\text{out}} , \\ &= \frac{n}{\kappa}d^{(\kappa)}\end{aligned} \tag{102}$$

where $d^{(\kappa)}$ is from (8) and the asymptotic limits of the combinatorial parts are given by:

$$\begin{aligned}\lim_{n\to\infty} q\binom{\frac{n}{q}}{\kappa}\frac{1}{n^{\kappa-1}} &= \lim_{n\to\infty}\frac{(n/q)!}{\kappa!(n/q-\kappa)!}\frac{q}{n^{\kappa-1}} = \frac{1}{(\kappa)!}\frac{(n/q)^{\kappa}q}{n^{\kappa-1}} = \frac{n}{\kappa!q^{\kappa-1}} \\ \lim_{n\to\infty}\left(\binom{n}{\kappa} - q\binom{\frac{n}{q}}{\kappa}\right)\frac{1}{n^{\kappa-1}} &= \frac{n}{(\kappa)!} - \frac{n}{(\kappa)!q^{\kappa-1}} = \frac{n(q^{\kappa-1}-1)}{\kappa!q^{\kappa-1}}\end{aligned} \ . \tag{103}$$

Then the average order $\hat{\kappa}$ is then calculated as the ratio of the total order of hyperedges to the total number of hyperedges:

$$\begin{aligned}\hat{\kappa} &= \frac{\sum_{\kappa\in\mathcal{K}} \kappa m^{(\kappa)}}{\sum_{\kappa\in\mathcal{K}} m^{(\kappa)}} \\ &= \frac{\sum_{\kappa\in\mathcal{K}} n d^{(\kappa)}}{\sum_{\kappa\in\mathcal{K}} \frac{n}{\kappa} d^{(\kappa)}} \\ &= \frac{d}{\sum_{\kappa\in\mathcal{K}} \frac{d^{(\kappa)}}{\kappa}}\end{aligned} \quad . \tag{104}$$

Next we aggregate perturbations in all leaves in the tree. Because the expectation of any perturbation ε is 0, we consider its variance

$$\begin{aligned}\mathbb{E}\left((\varepsilon^s)^2\right) &= \mathbb{E}\left(\left(\sum_{i=1}^{[d(\hat{\kappa}-1)]^s} \mu\lambda^s \varepsilon^{0;i}\right)^2\right) \\ &\approx \mu^2\lambda^{2s}\left[d(\hat{\kappa}-1)\right]^s \mathbb{E}\left((\varepsilon^0)^2\right)\end{aligned} \quad . \tag{105}$$

The stability of trivial fixed point is decided by

$$\begin{aligned}\sqrt[s]{\mu^2\lambda^{2s}[d(\hat{\kappa}-1)]^s} &< 1 \\ \exp\left(2\mathbb{E}\left(\log(\frac{1}{d^{|f|}(|f|-1)!q^{|f|}})\right)\right) q^2(c_{\text{in}}-c_{\text{out}})^2[d(\hat{\kappa}-1)] &< 1\end{aligned} \quad , \tag{106}$$

where

$$\begin{aligned}\mathbb{E}\left(\log(\frac{1}{d^{|f|}(|f|-1)!q^{|f|}})\right) &= \sum_{\kappa\in\mathcal{K}} \frac{m^{(\kappa)}}{\sum_{\kappa'\in\mathcal{K}} m^{(\kappa')}} log(\frac{1}{d^{(\kappa)}(\kappa-1)!q^{\kappa}}) \\ &= \sum_{\kappa\in\mathcal{K}} \frac{\frac{n}{\kappa}d^{(\kappa)}}{\sum_{\kappa'\in\mathcal{K}} \frac{n}{\kappa'} d^{(\kappa')}} log(\frac{1}{d^{(\kappa)}(\kappa-1)!q^{\kappa}}) \\ &= \sum_{\kappa\in\mathcal{K}} \frac{d^{(\kappa)}/\kappa}{\sum_{\kappa'\in\mathcal{K}} \frac{d^{(\kappa')}}{\kappa'}} \log(\frac{1}{d^{(\kappa)}(\kappa-1)!q^{\kappa}}) \\ &= \sum_{\kappa\in\mathcal{K}} \frac{\hat{\kappa}d^{(\kappa)}}{d\kappa} \log(\frac{1}{d^{(\kappa)}(\kappa-1)!q^{\kappa}})\end{aligned} \quad . \tag{107}$$

Here number of order-κ hyperedge $m^{(\kappa)}$ is from equation (102), average order (104) $\hat{\kappa} = \frac{d}{\sum_{\kappa\in\mathcal{K}} \frac{d^{(\kappa)}}{\kappa}}$ and average degree (10) $d = \sum_{\kappa\in\mathcal{K}} d^{(\kappa)}$. Because the detectability is equal to instability of trivial fixed point, so based on equation (106) and (107), the detectability

threshold of belief propagation is

$$\begin{aligned} q^2(c_{\text{in}}-c_{\text{out}})^2[d(\hat{\kappa}-1)]\prod_{\kappa\in\mathcal{K}}\left(\frac{1}{d^{(\kappa)}(\kappa-1)!q^{\kappa}}\right)^{2\frac{\hat{\kappa}d^{(\kappa)}}{d\kappa}} &> 1 \\ d(\hat{\kappa}-1)\prod_{\kappa\in\mathcal{K}}\left(\frac{q(c_{\text{in}}-c_{\text{out}})}{d^{(\kappa)}(\kappa-1)!q^{\kappa}}\right)^{2\frac{\hat{\kappa}d^{(\kappa)}}{d\kappa}} &> 1 \\ d(\hat{\kappa}-1)\prod_{\kappa\in\mathcal{K}}\left(\frac{(c_{\text{in}}-c_{\text{out}})}{d^{(\kappa)}(\kappa-1)!q^{\kappa-1}}\right)^{2\frac{\hat{\kappa}d^{(\kappa)}}{d\kappa}} &> 1 \\ d(\hat{\kappa}-1)\prod_{\kappa\in\mathcal{K}}\left(\frac{d_{\text{in}}^{(\kappa)}-d_{\text{out}}^{(\kappa)})}{d^{(\kappa)}}\right)^{2\frac{\hat{\kappa}d^{(\kappa)}}{d\kappa}} &> 1 \end{aligned} \quad , \tag{108}$$

where the simplification leading to the second line relies on:

$$\sum_{\kappa\in\mathcal{K}}\frac{\hat{\kappa}d^{(\kappa)}}{d\kappa}=1 \ , \tag{109}$$

while the last line is a direct result of substituting the explicit expressions for $d_{\text{in}}^{(\kappa)}$ and $d_{\text{out}}^{(\kappa)}$ as defined in (7). We denote the left formula of inequality (108) as

$$\text{SNR}_{\text{BP}} := d(\hat{\kappa}-1)\prod_{\kappa\in\mathcal{K}}\left(\frac{d_{\text{in}}^{(\kappa)}-d_{\text{out}}^{(\kappa)})}{d^{(\kappa)}}\right)^{2\frac{\hat{\kappa}d^{(\kappa)}}{d\kappa}} \quad . \tag{110}$$

S6 Supplementary experiment: detectability limits of symmetric HSBM

We use numeric experiment to verify the detectability limit of Bethe Hessian-based spectral clustering (9) and belief propagation (12). First, we run experiment on κ-uniform hypergraph to verify the detectability limit (67) in literature (17). Then we run experiment on nonuniform hypergraph with $q = 2$ to verify the SNR_{BH} (9) in literature (16) and SNR_{BP} (12) in this paper.

We use AMI (27) to evaluate the community detection result. Assume $\boldsymbol{\psi}^*$ is the real community label vector and $\boldsymbol{\psi}$ is detected community label vector. Then AMI is defined by

$$\text{AMI}(\boldsymbol{\psi},\boldsymbol{\psi}^*)=\frac{\text{MI}(\boldsymbol{\psi},\boldsymbol{\psi}^*)-\mathbb{E}(\text{MI}(\boldsymbol{\psi},\boldsymbol{\psi}^*))}{\frac{1}{2}(H(\boldsymbol{\psi})+H(\boldsymbol{\psi}^*))-\mathbb{E}(\text{MI}(\boldsymbol{\psi},\boldsymbol{\psi}^*))} \ , \tag{111}$$

where MI represent mutual information, $\mathbb{E}$ means the expectation. The AMI score lies in range $[0,1]$, with 0 means the detected communities have no difference to randomly detection, 1 means the detected communities is perfect fit the true communities.

For each of our experiments in this chapter, we fix the average degree d of hypergraph. Assume a hypergraph generated by nonuniform symmetric HSBM has average degree d, then

$$\begin{aligned} d &= \frac{\sum_{\kappa\in\mathcal{K}} q\binom{\frac{n}{q}}{\kappa}\kappa\frac{c_{\text{in}}}{n^{\kappa-1}} + \left(\binom{n}{\kappa} - q\binom{\frac{n}{q}}{\kappa}\right)\kappa\frac{c_{\text{out}}}{n^{\kappa-1}}}{n} \\ &= \sum_{\kappa\in\mathcal{K}} q\binom{\frac{n}{q}}{\kappa}\frac{\kappa}{n^{\kappa}}c_{\text{in}} + \left(\binom{n}{\kappa} - q\binom{\frac{n}{q}}{\kappa}\right)\frac{\kappa}{n^{\kappa}}c_{\text{out}} \end{aligned} . \tag{112}$$

After given d and setting $\epsilon = \frac{c_{\text{out}}}{c_{\text{in}}}$, we have

$$\begin{aligned} c_{\text{in}} &= \frac{d}{\sum_{\kappa\in\mathcal{K}} q\binom{\frac{n}{q}}{\kappa}\frac{\kappa}{n^{\kappa}} + \epsilon\left(\binom{n}{\kappa} - q\binom{\frac{n}{q}}{\kappa}\right)\frac{\kappa}{n^{\kappa}}} \\ c_{\text{out}} &= \frac{d\epsilon}{\sum_{\kappa\in\mathcal{K}} q\binom{\frac{n}{q}}{\kappa}\frac{\kappa}{n^{\kappa}} + \epsilon\left(\binom{n}{\kappa} - q\binom{\frac{n}{q}}{\kappa}\right)\frac{\kappa}{n^{\kappa}}} \end{aligned} . \tag{113}$$

We can generate a hypergraph by sample from Bernoulli distribution with probability $c_{\text{in}}/n^{\kappa-1}$ or $c_{\text{out}}/n^{\kappa-1}$ for each of $\binom{n}{\kappa}$ possible hyperedges and $\kappa \in \mathcal{K}$. When n is large enough, the high time complexity $O(n^{\max(\mathcal{K})})$ of this method makes it difficult to apply. So we use another more efficient approach to generate hypergraph with HSBM. The details of the approach is presented in Section S7.1.

Beyond the Bethe Hessian spectral clustering Algorithm S1, we also implement the belief propagation with the assumption of symmetric HSBM based on the code in paper (18). The main difference is the calculation of external field (81) shown in Section S5.2 and the update of hyperedge to node message shown in Section S7.2.

S6.1 Experiment on uniform hypergraph

For a κ-uniform hypergraph generated by κ-uniform symmetric HSBM, we can get the critical ϵ^* at detectability threshold $\text{SNR}_{\text{BH}} = 1$ (9) when $\mathcal{K} = \{\kappa\}$. Considering assortative case $d_{\text{in}}^{(\kappa)} > d_{\text{out}}^{(\kappa)}$, then at the detectability threshold, we have

$$(\kappa-1)(d_{\text{in}}^{(\kappa)} - d_{\text{out}}^{(\kappa)}) = \sqrt{(\kappa-1)d^{(\kappa)}} \ . \tag{114}$$

Combined with average κ-degree (8), we can get the $d_{\text{in}}^{(\kappa)}, d_{\text{out}}^{(\kappa)}$ at the detectability threshold:

$$\begin{aligned} d_{\text{in}}^{(\kappa)} &= \frac{1}{q^{\kappa-1}}\left(d^{(\kappa)} + (q^{\kappa-1}-1)\sqrt{\frac{d^{(\kappa)}}{\kappa-1}}\right) \\ d_{\text{out}}^{(\kappa)} &= \frac{1}{q^{\kappa-1}}\left(d^{(\kappa)} - \sqrt{\frac{d^{(\kappa)}}{\kappa-1}}\right) \end{aligned}. \tag{115}$$

With setting $\epsilon = \frac{c_{\text{out}}}{c_{\text{in}}}$ and relation between $d_{\text{in}}^{(\kappa)}, d_{\text{out}}^{(\kappa)}$ and $c_{\text{in}}, c_{\text{out}}$ (7), the critical ϵ^* is

$$\epsilon^* = \frac{d_{\text{out}}^{(\kappa)}}{d_{\text{in}}^{(\kappa)}} = \frac{\sqrt{d^{(\kappa)}(\kappa-1)} - 1}{\sqrt{d^{(\kappa)}(\kappa-1)} + q^{\kappa-1} - 1} \tag{116}$$

which is also shown in paper (17).

We fix the κ-uniform hypergraph size $n = 30000$, number of communities $q = 2$, order $\kappa = 3$ and average degree $d = 10$. For different ϵ, we calculate the c_{in} and c_{out} with equation (113) and generate a hypergraph with κ-uniform symmetric HSBM. Then we use Bethe Hessian method to detect communities of the hypergraph. As the result shown in Figure S6, the critical ϵ^* is close to the point that AMI_{BH} start to be 0. In this experiment, the $\epsilon^* = 0.465$.

S6.2 Experiment on nonuniform hypergraph

We fix the nonuniform hypergraph size $n = 30000$, number of communities $q = 2$, $\mathcal{K} = \{2, 3\}$ and average degree $d = 10$. For different ϵ, we calculate the c_{in} and c_{out} with equation (113) and generate a hypergraph with nonuniform symmetric HSBM. Then we use Bethe Hessian spectral clustering and belief propagation to detect communities of the hypergraph, the result is recorded as AMI_{BH} and AMI_{BP}.

We define the critical ϵ_{BH}^* and ϵ_{BP}^* corresponding with $\text{SNR}_{\text{BH}} = 1$ (9) and $\text{SNR}_{\text{BP}} = 1$ (12). As the result shown in Figure S7, the ϵ_{BH}^* is close to the point that AMI_{BH} start to be 0, the ϵ_{BP}^* is close to the point that AMI_{BP} start to be 0. These results verify that the detectability SNR_{BH} (9) of spectral clustering with Bethe Hessian is same as the non-backtracking matrix. And SNR_{BH} is weaker than detectability SNR_{BP} (12) of belief propagation, which we believe is the theoretical detectability limit.

S6.3 Comparison with more baseline methods

To evaluate the performance of our proposed method, we compare it against two established baselines: the Laplacian-based spectral clustering framework (44) and the h-Louvain algorithm, which is based on hypergraph modularity optimization (45). For the Laplacian, we give the true number of communities q to the spectral clustering, which

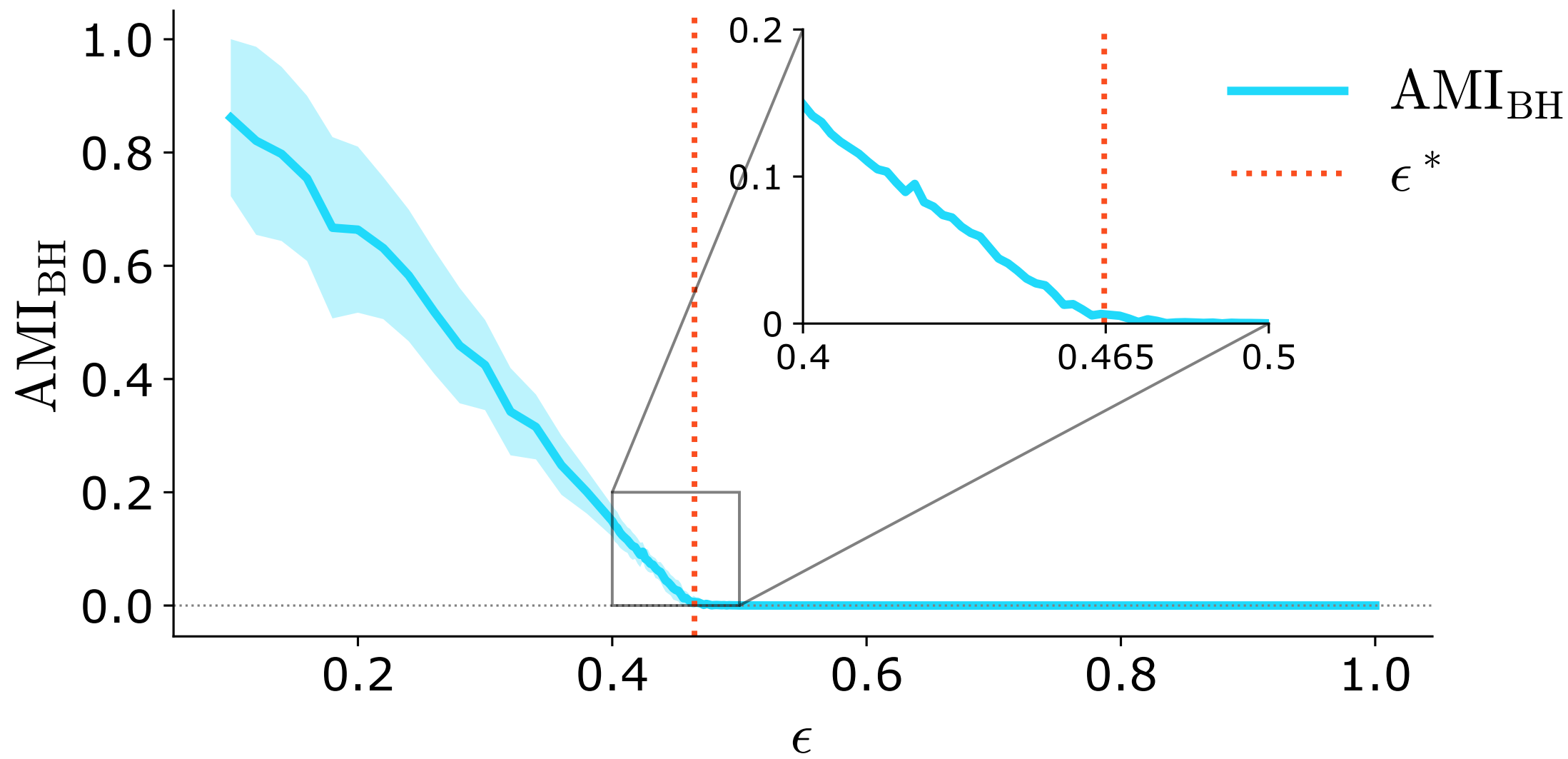

Figure S6: ***Detectability phase transition of the Bethe Hessian in 3-uniform hypergraphs.*** *The experiment run on* 3*-uniform hypergraph. The blue line is the* $\mathrm{AMI_{BH}}$ *at different* ϵ*. For each point, we run experiment for 50 times; solid lines show means and shaded bands show 95% confidence intervals. The orange vertical dash line is the critical* ϵ^* *where the detectability phase transition happen.*

means we select the smallest q eigenvectors of Laplacian matrix for clustering. The h-Louvain algorithm have its own model selection approach, so we can not restrict it to detect q communities.

Under the large-scale parameter configuration used in Figure 2 ($n = 30000, q = 3$), the h-Louvain algorithm is computationally prohibitive due to its excessive execution time. Consequently, for this setting, we restrict our comparison to Laplacian spectral clustering, as illustrated in Figure S8.

To facilitate a comprehensive comparison that includes h-Louvain, we conducted additional experiments on a reduced scale with $n = 3000$. These results are presented in Figure S9, where the performance of all four methods is evaluated. We also compare their running time in Figure S10.

The Figure S8, Figure S9 illustrate that our proposed Bethe Hessian method has a better performance than Hypergraph Laplacian and Hypergraph Louvain and approaches the detectability limit more closely. Figure S10 shows that BH is more efficient in general, particularly comparing with BP when it is close to the detectability limit.

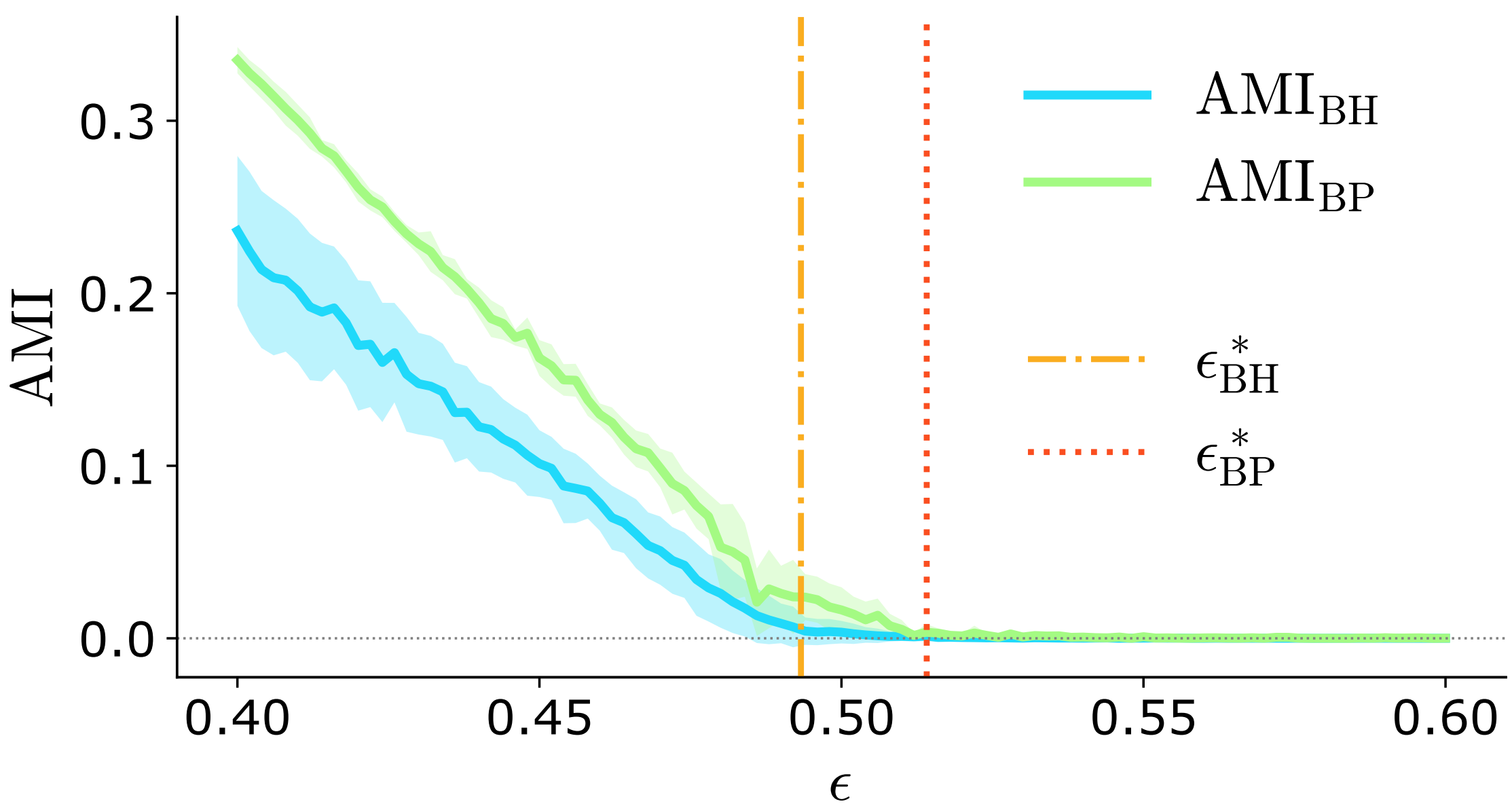

Figure S7: ***Detectability phase transition of the Bethe Hessian in nonuniform hypergraphs.*** *The experiment run on nonuniform hypergraph with 2 communities. For each ϵ, we run bethe hessian method 100 times and belief propagation for 10 times; solid lines show means and shaded bands show 95% confidence intervals.*

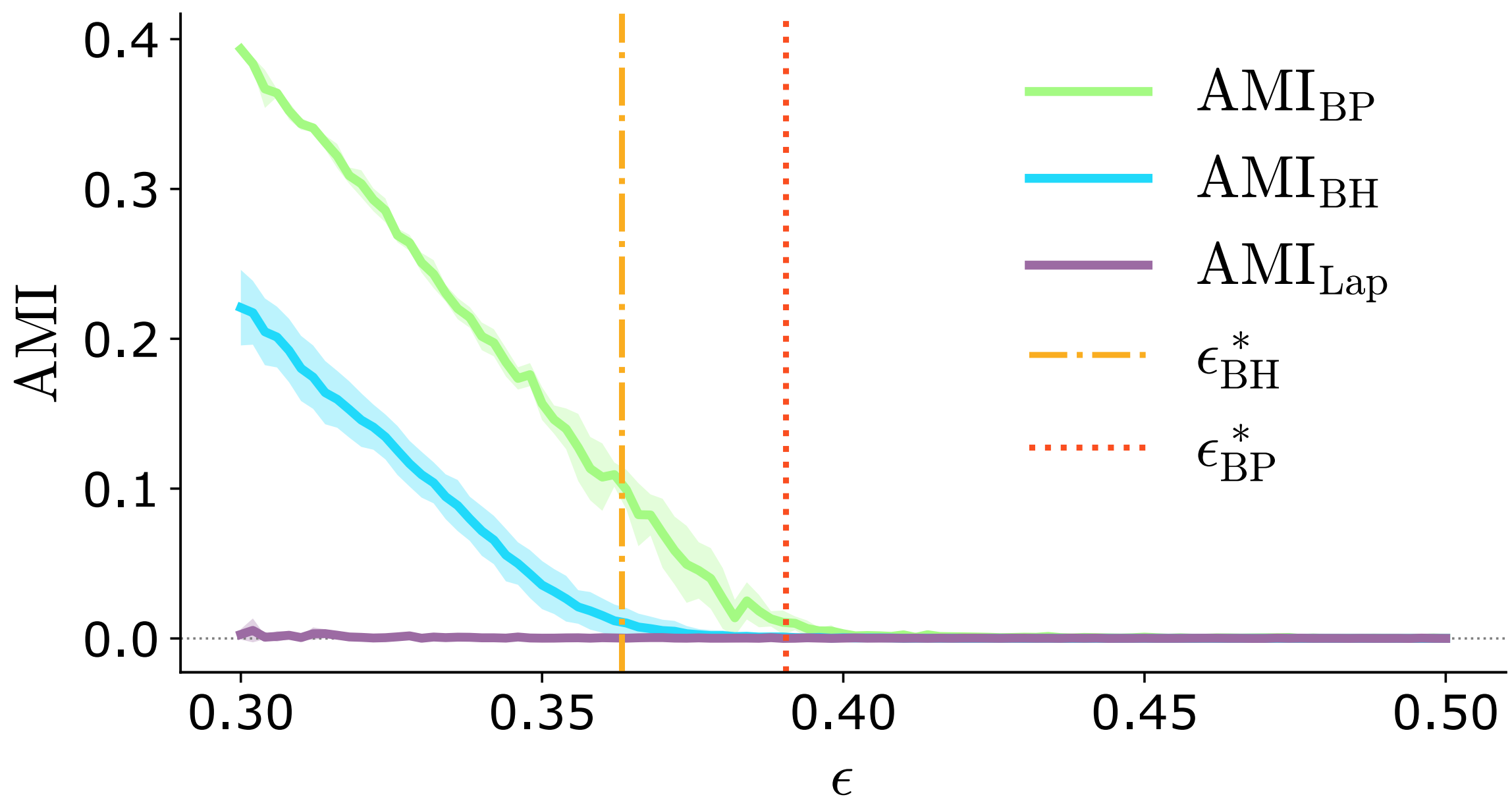

Figure S8: ***Algorithmic comparison of laplacian spectral clustering across detectability levels.*** *A comparison experiment with nonuniform hypergraphs with* $n = 30000$ *nodes,* $q = 3$ *communities,* $\mathcal{K} = \{2, 3\}$ *hyperedge orders and* $d = 10$ *mean node degree. For each* ϵ*, we run Laplacian spectral clustering 10 times; solid lines show means and shaded bands show 95% confidence intervals.*

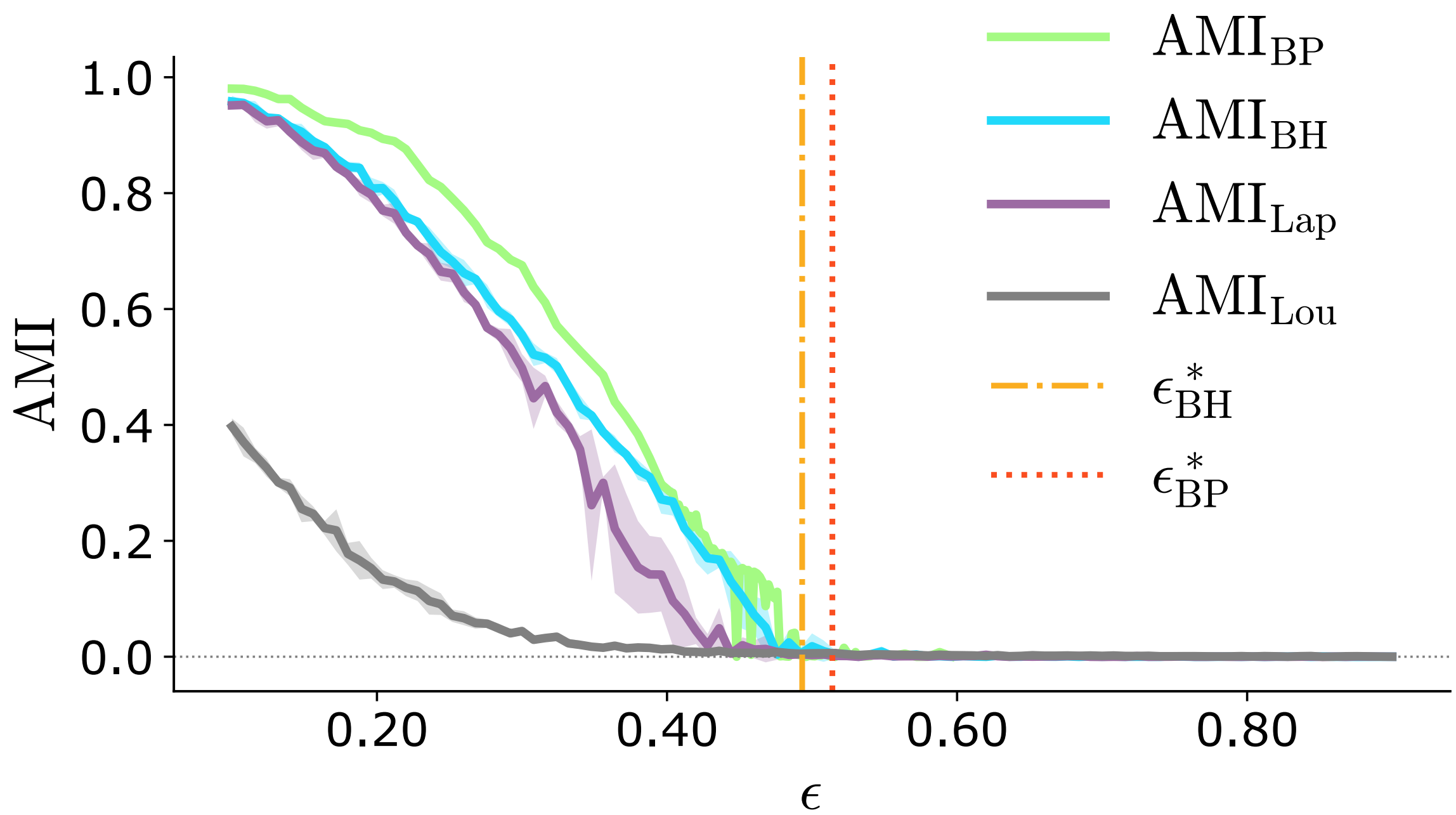

Figure S9: ***Algorithmic comparison across detectability levels in nonuniform HSBMs.*** *A comparison experiment with nonuniform hypergraphs with $n = 3000$ nodes, $q = 2$ communities, $\mathcal{K} = \{2, 3\}$ hyperedge orders and $d = 10$ mean node degree. For each ϵ, we run each method 10 times; solid lines show means and shaded bands show 95% confidence intervals.*

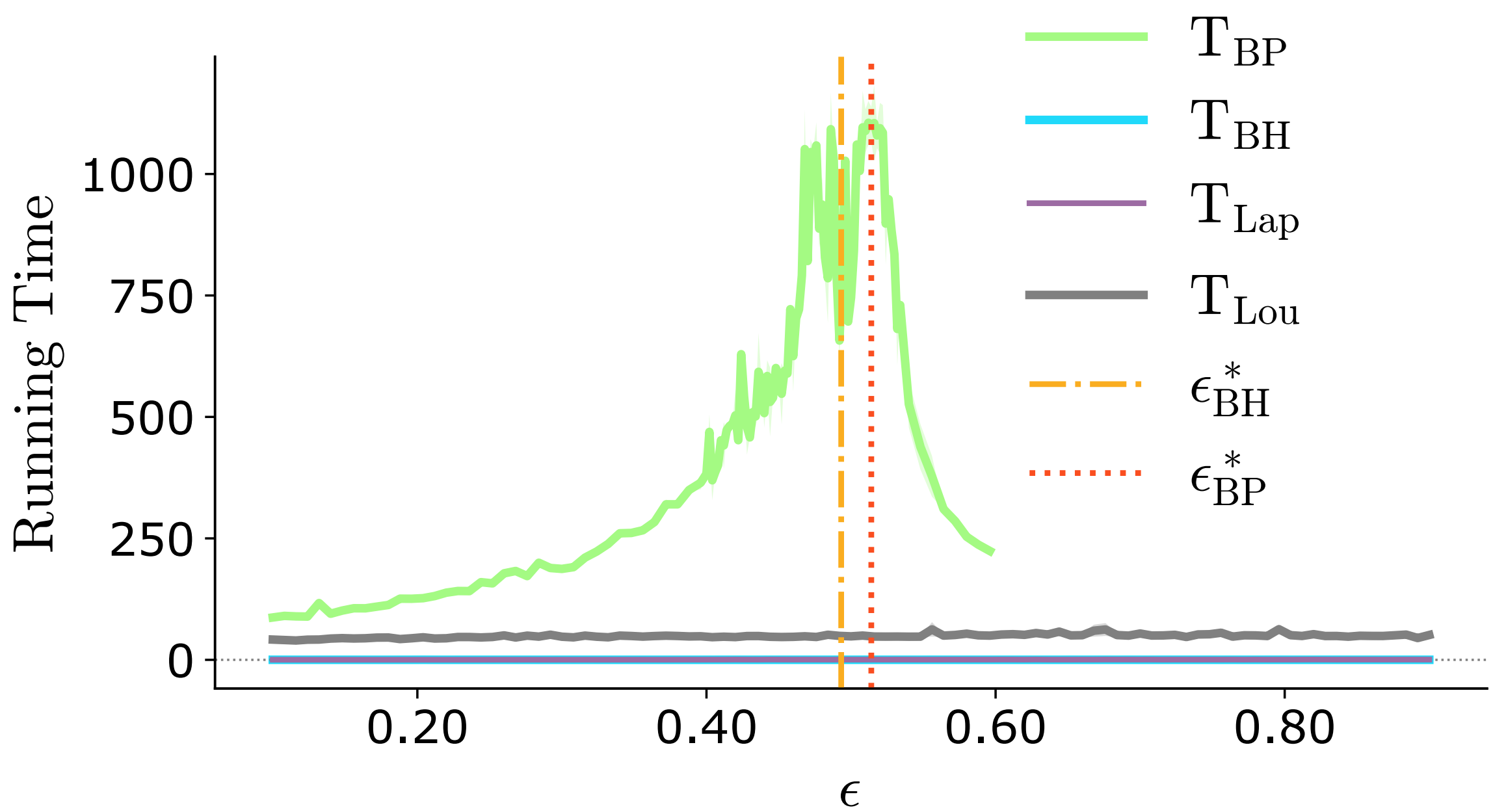

Figure S10: ***Running time comparison of community detection methods in nonuniform hypergraphs.*** *A running time comparison experiment with nonuniform hypergraphs with* $n = 3000$ *nodes,* $q = 2$ *communities,* $\mathcal{K} = \{2, 3\}$ *hyperedge orders and* $d = 10$ *mean node degree. For each* ϵ*, we run each method 10 times; solid lines show means and shaded bands show 95% confidence intervals.*

S7 Implementation details of Experiment

S7.1 Efficient approach to generate hypergraph with HSBM

The main idea for efficiently generating hypergraph with HSBM is to sample κ-order hyperedges for each possible combination of community assignment of the κ nodes and $\kappa \in \mathcal{K}$. We first need to find all possible ordered combination of q communities that each community can exist multiple times. For example, if $q = 2$, then the combination of community assignment for a 3-order hyperedge could be $\{0, 0, 0\}$, $\{0, 0, 1\}$, $\{0, 1, 1\}$, $\{1, 1, 1\}$.

Then, for each of community assignment $\overrightarrow{\psi}$, we calculate the expected number of generated hyperedge as

$$m_{\overrightarrow{\psi}} = \sum_{\psi \in [q]} \binom{n \times n_\psi}{\# \left\{ \psi \text{ in } \overrightarrow{\psi} \right\}} \mathbf{\Omega}_{\overrightarrow{\psi}} \quad , \tag{117}$$

where $\# \left\{ \psi \text{ in } \overrightarrow{\psi} \right\}$ means the occurrence count of ψ in the $\overrightarrow{\psi}$.

Finally, for each of community assignment $\overrightarrow{\psi}$, we sample hyperedges $m_{\overrightarrow{\psi}}$ times. Each sampling we random select $\# \left\{ \psi \text{ in } \overrightarrow{\psi} \right\}$ nodes in community ψ for each $\psi \in \overrightarrow{\psi}$. With this approach, we reduced the time wasted on hyperedges that will not be generated.

S7.2 Dynamic programming for updating hyperedge to node message

We adjust the belief propagation code of paper (18) for the HSBM in this paper. The update of hyperedge to node message can be represented as

$$\log(\hat{b}^{e\to i}_{\psi_i}) = -\log Z^{e\to i} + \underbrace{\log(\sum_{\overrightarrow{\psi_{e/i}} \in [q]^{|e|-1}} \boldsymbol{C}_{\overrightarrow{\psi_e}} \prod_{j \in e/i} b^{j\to e}_{\psi_j})}_{\eta(e,i,\psi_i)} \quad , \tag{118}$$

where $Z^{e\to i}$ is the normalization term. For simplicity, we assume $e = \{1, 2, ..., \kappa\}$, $i = 1$, then another term $\eta(e, i, \psi_i)$ can be expand as

$$\exp(\eta(e, 1, \psi_1)) = \sum_{\psi_2 \in [q]} \sum_{\psi_3 \in [q]} ... \sum_{\psi_\kappa \in [q]} \boldsymbol{C}_{\overrightarrow{\psi_e}} b^{2\to e}_{\psi_2} b^{3\to e}_{\psi_3} ... b^{\kappa\to e}_{\psi_\kappa} \quad . \tag{119}$$

There are $(\kappa - 1)q^{\kappa-1}$ times of multiplication if we directly calculate $\eta(e, 1, \psi_1)$, which is unacceptable for high order κ. However, based on the specific $\boldsymbol{C}$ (6) in the symmetric

HSBM, we can calculate $\eta(e,i,\psi_i)$ with dynamic programming

$$\begin{aligned}
\exp(\eta(e,1,\psi_1)) =& \sum_{\psi_\kappa\in[q]} b^{\kappa\to e}_{\psi_\kappa} \sum_{\psi_2\in[q]}\sum_{\psi_3\in[q]}\dots\sum_{\psi_{\kappa-1}\in[q]} \boldsymbol{C}_{\overrightarrow{\psi_e}} b^{2\to e}_{\psi_2} b^{3\to e}_{\psi_3}\dots b^{\kappa-1\to e}_{\psi_{\kappa-1}} \\
=& \sum_{\psi_\kappa\in[q]/\psi_1} b^{\kappa\to e}_{\psi_\kappa} \underbrace{\sum_{\psi_2\in[q]}\sum_{\psi_3\in[q]}\dots\sum_{\psi_{\kappa-1}\in[q]} c_{\text{out}} b^{2\to e}_{\psi_2} b^{3\to e}_{\psi_3}\dots b^{\kappa-1\to e}_{\psi_{\kappa-1}}}_{\exp(\eta(e/\kappa,1,\psi_1))+(c_{\text{out}}-c_{\text{in}})b^{2\to e}_{\psi_1}b^{3\to e}_{\psi_1}\dots b^{\kappa-1\to e}_{\psi_1}} \\
&+ b^{\kappa\to e}_{\psi_1} \underbrace{\sum_{\psi_2\in[q]}\sum_{\psi_3\in[q]}\dots\sum_{\psi_{\kappa-1}\in[q]} \boldsymbol{C}_{\overrightarrow{\psi_e}} b^{2\to e}_{\psi_2} b^{3\to e}_{\psi_3}\dots b^{\kappa-1\to e}_{\psi_{\kappa-1}}}_{\exp(\eta(e/\kappa,1,\psi_1))} \\
=& \sum_{\psi_\kappa\in[q]} b^{\kappa\to e}_{\psi_\kappa} \exp(\eta(e/\kappa,1,\psi_1)) + (c_{\text{out}}-c_{\text{in}}) \sum_{\psi_\kappa\in[q]/\psi_1} b^{\kappa\to e}_{\psi_\kappa} b^{2\to e}_{\psi_1} b^{3\to e}_{\psi_1}\dots b^{\kappa-1\to e}_{\psi_1} \\
=& \exp(\eta(e/\kappa,1,\psi_1)) + (c_{\text{out}}-c_{\text{in}}) \sum_{\psi_\kappa\in[q]/\psi_1} b^{\kappa\to e}_{\psi_\kappa} b^{2\to e}_{\psi_1} b^{3\to e}_{\psi_1}\dots b^{\kappa-1\to e}_{\psi_1}
\end{aligned} \quad . \tag{120}$$

By removing nodes of e one by one from κ, finally we can get

$$\begin{aligned}
\exp(\eta(\{1,2\},1,\psi_1)) =& c_{\text{in}} b^{2\to\{1,2\}}_{\psi_1} + c_{\text{out}} \sum_{\psi_2\in[q]/\psi_1} b^{2\to\{1,2\}}_{\psi_2} \\
=& c_{\text{in}} + (c_{\text{out}}-c_{\text{in}}) \sum_{\psi_2\in[q]/\psi_1} b^{2\to\{1,2\}}_{\psi_2}
\end{aligned} \quad . \tag{121}$$

The number of multiplication of (121) is $\kappa+\kappa-1+..+2 = O(\kappa^2)$ which is smaller than directly calculation of (119) for high order κ.

S7.3 The calculation of free energy

According to (18), the free energy of the belief propagation on hypergraph can be estimated as

$$\mathcal{F} \approx -\sum_{i\in[n]} f_i + \sum_{e\in\boldsymbol{E}} (|e|-1) f_e \quad , \tag{122}$$

where, based on our HSBM, the f_i and f_e is

$$\begin{aligned}
f_i &= \log\left(\sum_{\psi_i\in[q]} n_{\psi_i} \prod_{e\in\partial i\cap\boldsymbol{E}} \sum_{\overrightarrow{\psi_{e/i}}\in[q]^{|e|-1}} \left(\boldsymbol{\Omega}_{\overrightarrow{\psi_e}}\right)^{A_e} \left(1-\boldsymbol{\Omega}_{\overrightarrow{\psi_e}}\right)^{1-A_e} \prod_{j\in\partial e/i} b^{j\to e}_{\psi_j}\right) \\
f_e &= \log\left(\sum_{\overrightarrow{\psi_e}\in[q]^{|e|}} \left(\boldsymbol{\Omega}_{\overrightarrow{\psi_e}}\right)^{A_e} \left(1-\boldsymbol{\Omega}_{\overrightarrow{\psi_e}}\right)^{1-A_e} \prod_{j\in\partial e} b^{j\to e}_{\psi_j}\right)
\end{aligned} \tag{123}$$

Firstly, let's see f_i. Based on the discussion of hyperedge to node belief $\hat{b}_{\psi_i}^{e\to i}$ in Section S5.1, we have

$$\begin{aligned}
f_i =& \log\left(\sum_{\psi_i\in[q]} n_{\psi_i} \prod_{e\in\partial i\cap\mathcal{E}}\left(\sum_{\overrightarrow{\psi_{e/i}}\in[q]^{|e|-1}}\left(\boldsymbol{\Omega}_{\overrightarrow{\psi_e}}\right)\prod_{j\in\partial e/i} b_{\psi_j}^{j\to e}\right)\cdot\right.\\
&\left.\prod_{e\in\partial i\cap\boldsymbol{E}/\mathcal{E}}\left(\sum_{\overrightarrow{\psi_{e/i}}\in[q]^{|e|-1}}\left(1-\boldsymbol{\Omega}_{\overrightarrow{\psi_e}}\right)\prod_{j\in\partial e/i} b_{\psi_j}^{j\to e}\right)\right)\\
=& \log\left(\sum_{\psi_i\in[q]} n_{\psi_i} \prod_{e\in\partial i\cap\mathcal{E}}\left(\frac{1}{n^{|e|-1}}\sum_{\overrightarrow{\psi_{e/i}}\in[q]^{|e|-1}}\boldsymbol{C}_{\overrightarrow{\psi_e}}\prod_{j\in\partial e/i} b_{\psi_j}^{j\to e}\right)\cdot\right.\\
&\left.\prod_{e\in\partial i\cap\boldsymbol{E}/\mathcal{E}} e^{-\frac{1}{n^{|e|-1}}\sum_{\overrightarrow{\psi_{e/i}}\in[q]^{|e|-1}}\boldsymbol{C}_{\overrightarrow{\psi_e}}\prod_{j\in e/i} b_{\psi_j}^{j}}\right)\\
=& \log\left(\sum_{\psi_i\in[q]} n_{\psi_i} e^{-h_{\psi_i}^{i}} \prod_{e\in\partial i\cap\mathcal{E}}\frac{1}{n^{|e|-1}}\sum_{\overrightarrow{\psi_{e/i}}\in[q]^{|e|-1}}\boldsymbol{C}_{\overrightarrow{\psi_e}}\prod_{j\in\partial e/i} b_{\psi_j}^{j\to e}\right)\\
=& \log\left(\sum_{\psi_i\in[q]} n_{\psi_i} e^{-h_{\psi_i}} \prod_{e\in\partial i\cap\mathcal{E}}\frac{1}{n^{|e|-1}} e^{\eta(e,i,\psi_i)}\right)
\end{aligned}, \tag{124}$$

where external field h_{ψ_i} is defined in Section S5.2 and $\eta(e,i,\psi_i)$ is defined in Section S7.2.

About the second summation of $\mathcal{F}$ (122), we have:

$$\begin{aligned}
\sum_{e\in\boldsymbol{E}}(|e|-1)f_e =& \sum_{e\in\mathcal{E}}\log\left(\sum_{\overrightarrow{\psi_e}\in[q]^{|e|}}\boldsymbol{\Omega}_{\overrightarrow{\psi_e}}\prod_{j\in\partial e} b_{\psi_j}^{j\to e}\right)^{|e|-1} +\\
&\sum_{e\in\boldsymbol{E}/\mathcal{E}}\log\left(\sum_{\overrightarrow{\psi_e}\in[q]^{|e|}}\left(1-\boldsymbol{\Omega}_{\overrightarrow{\psi_e}}\right)\prod_{j\in\partial e} b_{\psi_j}^{j\to e}\right)^{|e|-1}
\end{aligned} \tag{125}$$

When $e\in\mathcal{E}$, the first summation of (125) is

$$\log\left(\sum_{\overrightarrow{\psi_e}\in[q]^{|e|}}\boldsymbol{\Omega}_{\overrightarrow{\psi_e}}\prod_{j\in\partial e} b_{\psi_j}^{j\to e}\right)^{|e|-1} = (|e|-1)\log\left(\frac{1}{n^{|e|-1}}\underbrace{\sum_{\overrightarrow{\psi_e}\in[q]^{|e|}}\boldsymbol{C}_{\overrightarrow{\psi_e}}\prod_{j\in\partial e} b_{\psi_j}^{j\to e}}_{\tilde{\eta}(e)}\right) \tag{126}$$

Similar with Section S7.2, we also use dynamic programming to calculate $\tilde{\eta}(e)$. Assume $e = \{1, 2, ..., m\}$, set $\tilde{\eta}(e) = \tilde{\eta}(e, m)$, expand $\tilde{\eta}(e, m)$ we have

$$\begin{aligned}
\tilde{\eta}(e,m) &= \sum_{\psi_m \in [q]} b_{\psi_m}^{m\to e} \sum_{\psi_1 \in [q]} \sum_{\psi_2 \in [q]} \cdots \sum_{\psi_{m-1} \in [q]} \boldsymbol{C}_{\overrightarrow{\psi_e}} \prod_{j \in \{1,2,\ldots,m-1\}} b_{\psi_j}^{j\to e} \\
&= \sum_{\psi_m \in [q]} b_{\psi_m}^{m\to e} \left(\tilde{\eta}(e, m-1) + (c_{\text{out}} - c_{\text{in}}) \sum_{\psi \in [q]/\psi_m} \prod_{j \in \{1,2,\ldots,m-1\}} b_{\psi}^{j\to e} \right) \\
&= \tilde{\eta}(e, m-1) + (c_{\text{out}} - c_{\text{in}}) \sum_{\psi_m \in [q]} b_{\psi_m}^{m\to e} \sum_{\psi \in [q]/\psi_m} \prod_{j \in \{1,2,\ldots,m-1\}} b_{\psi}^{j\to e}
\end{aligned} \tag{127}$$

Through dynamic programming shown in (127), we can reduce the number of multiplication from $q^m(m-1)$ to $\sum_{m=m}^{2} q(q(m-2)+1)+1 = O(q^2m^2)$. The terminate condition for above dynamic programming $\tilde{\eta}(e, 2)$ is

$$\begin{aligned}
\tilde{\eta}(e,2) &= \sum_{\psi_1 \in [q]} \left(c_{\text{in}} b_{\psi_1}^{1\to e} b_{\psi_1}^{2\to e} + c_{\text{out}} b_{\psi_1}^{1\to e} \sum_{\psi_2 \in [q]/\psi_1} b_{\psi_2}^{2\to e} \right) \\
&= \sum_{\psi_1 \in [q]} \left((c_{\text{in}} - c_{\text{out}}) b_{\psi_1}^{1\to e} b_{\psi_1}^{2\to e} + c_{\text{out}} b_{\psi_1}^{1\to e} \sum_{\psi_2 \in [q]} b_{\psi_2}^{2\to e} \right) \\
&= c_{\text{out}} + (c_{\text{in}} - c_{\text{out}}) \sum_{\psi_1 \in [q]} b_{\psi_1}^{1\to e} b_{\psi_1}^{2\to e}
\end{aligned} \tag{128}$$

When $e \notin \mathcal{E}$, the second summation is

$$
\begin{aligned}
&\sum_{e\in \boldsymbol{E}/\mathcal{E}} \log\left(\sum_{\overrightarrow{\psi_e}\in[q]^{|e|}} \left(1-\boldsymbol{\Omega}_{\overrightarrow{\psi_e}}\right)\prod_{j\in\partial e} b_{\psi_j}^{j\to e}\right)^{|e|-1} \\
=&\log\left(\prod_{e\in \boldsymbol{E}/\mathcal{E}}\left[\sum_{\overrightarrow{\psi_e}\in[q]^{|e|}} \left(1-\boldsymbol{\Omega}_{\overrightarrow{\psi_e}}\right)\prod_{j\in\partial e} b_{\psi_j}^{j\to e}\right]^{|e|-1}\right) \\
=&\log\left(\prod_{e\in \boldsymbol{E}/\mathcal{E}}\left[1-\frac{1}{n^{|e|-1}}\sum_{\overrightarrow{\psi_e}\in[q]^{|e|}} \boldsymbol{C}_{\overrightarrow{\psi_e}}\prod_{j\in\partial e} b_{\psi_j}^{j\to e}\right]^{|e|-1}\right) \\
\approx&\log\left(\prod_{e\in \boldsymbol{E}} e^{-\frac{|e|-1}{n^{|e|-1}}\sum_{\overrightarrow{\psi_e}\in[q]^{|e|}} \boldsymbol{C}_{\overrightarrow{\psi_e}}\prod_{j\in\partial e} b_{\psi_j}^{j\to e}}\right) \\
\approx&\sum_{e\in \boldsymbol{E}}\frac{1-|e|}{n^{|e|-1}}\sum_{\overrightarrow{\psi_e}\in[q]^{|e|}} \boldsymbol{C}_{\overrightarrow{\psi_e}}\prod_{j\in\partial e} b_{\psi_j}^{j} \\
=&\sum_{\kappa\in\mathcal{K}}\frac{1-\kappa}{n^{\kappa-1}}\sum_{f\in\boldsymbol{E}^\kappa}\left[(c_{\text{in}}-c_{\text{out}})\sum_{\psi\in[q]}\prod_{j\in\partial f} b_{\psi}^{j} + c_{\text{out}}\sum_{\overrightarrow{\psi_e}\in[q]^{|e|}}\prod_{j\in\partial f} b_{\psi_j}^{j}\right] \\
=&\sum_{\kappa\in\mathcal{K}}\frac{1-\kappa}{n^{\kappa-1}}\left[\binom{n}{\kappa}c_{\text{out}} + (c_{\text{in}}-c_{\text{out}})\sum_{\psi\in[q]}\frac{1}{\kappa!}\left(\sum_{j\in[n]} b_{\psi}^{j}\right)^{\kappa}\right] \\
\approx&\sum_{\kappa\in\mathcal{K}}\left[(1-\kappa)c_{\text{out}} + \frac{(1-\kappa)(c_{\text{in}}-c_{\text{out}})}{n^{\kappa-1}\kappa!}\sum_{\psi\in[q]}\left(\sum_{j\in[n]} b_{\psi}^{j}\right)^{\kappa}\right]
\end{aligned}
\quad . \tag{129}
$$

With above equations, we can calculate free energy (122) efficiently.

S8 Supplementary analysis: community structure dominated by hyperedge order and shape.

We designed a supplementary experiment to investigate how the dominance of different hyperedge orders influences the detected community structure using Bethe Hessian-based Spectral Clustering. We consider a hypergraph of n nodes equally partitioned into $q = 4$ communities, $\{0, 1, 2, 3\}$. The structure is designed to favor two competing community patterns: all the κ-hyperedges are placed between nodes in communities $\{0, 2\}$ or $\{1, 3\}$, all the κ^*-hyperedges are placed between communities $\{0, 1\}$ or $\{2, 3\}$, as illustrated in Figure S11.

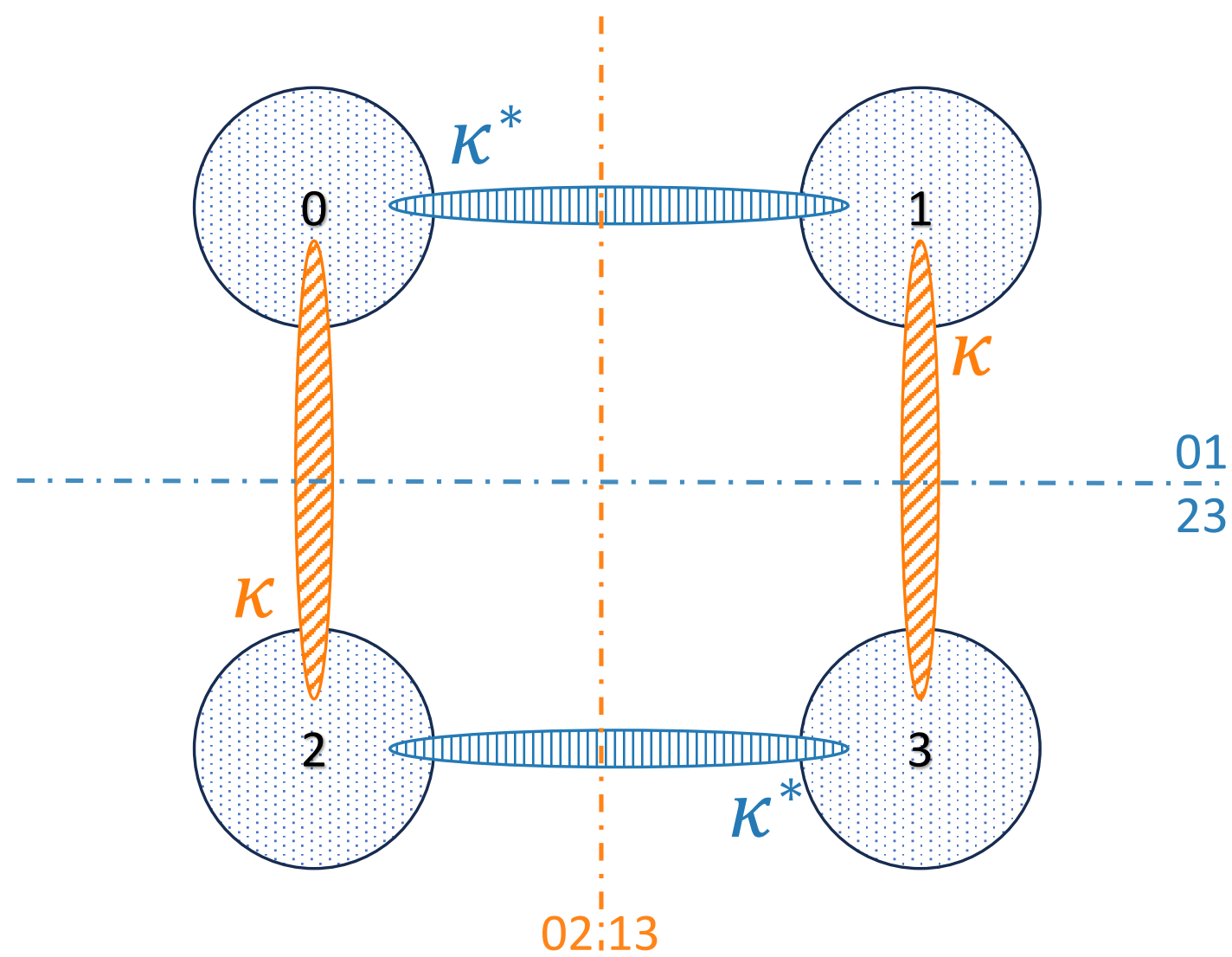

Figure S11: ***Experimental hypergraph topologies for order analysis on community detection.*** *An illustration of community structure of hypergraph used in experiment for exploring the effect of order on community detection.*

If we apply Bethe Hessian-based spectral clustering algorithm S1 to detect 2 communities in this hypergraph considering only the assortative case, two distinct, coarse-grained community structures could be detected:

- κ^*-dominated structure with communities $\{0, 1\}$ are merged into one community, $\{2, 3\}$ are merged into the second.
- κ-dominated structure with communities $\{0, 2\}$ are merged into one community, $\{1, 3\}$ are merged into the second.

Intuitively, if the number of κ-order hyperedges $|\mathcal{E}^{(\kappa)}|$ significantly outweighs $|\mathcal{E}^{(\kappa^*)}|$, the κ-dominated community structure should prevail. Our objective is to determine the critical switching ratio of $|\mathcal{E}^{(\kappa)}|$ and $|\mathcal{E}^{(\kappa^*)}|$, at which the detected community structure transitions from the κ^*-dominated structure to the κ-dominated one.

S8.1 Simple case with $\kappa = 2$, $\kappa^* = 3$

We first consider a simplified scenario with $\kappa = 2$, $\kappa^* = 3$. To generate the hypergraph shown in Figure S11 using the HSBM, we define the affinity tensors as follows:

$$
\begin{aligned}
\boldsymbol{C}^{(2)}_{\boldsymbol{z}} &= \begin{cases} a, & \boldsymbol{z} \in \{(0,2),(2,0),(1,3),(3,1)\} \\ 0, & \textit{otherwise} \end{cases} \\
\boldsymbol{C}^{(3)}_{\boldsymbol{z}} &= \begin{cases} a^*, & \boldsymbol{z} \in \begin{matrix} \{(0,0,1),(0,1,0),(1,0,1), \\ (0,1,1),(1,0,1),(1,1,0), \\ (2,2,3),(2,3,2),(3,2,2), \\ (2,3,3),(3,2,3),(3,3,2)\} \end{matrix} \\ 0, & \textit{otherwise} \end{cases}
\end{aligned}
, \tag{130}
$$

where a and a^* are adjusted parameters. By observing the definition of $\widehat{c}^{(\kappa)}_{ab}$ in Section S4, we can construct the matrix $\widehat{\boldsymbol{C}}^{(\kappa)}$ by fixing two dimensions and aggregating the remaining $\kappa - 2$ dimension of scaled affinity tensor $\frac{1}{q^{\kappa-2}(\kappa-2)!}\boldsymbol{C}^{(\kappa)}$. In this scenario, we calculate the corresponding 4×4 matrices $\widehat{\boldsymbol{C}}^{(2)}$ and $\widehat{\boldsymbol{C}}^{(3)}$:

$$
\begin{aligned}
\widehat{\boldsymbol{C}}^{(2)} &= \frac{1}{4^{2-2}(2-2)!} \begin{bmatrix} 0, & 0, & a, & 0 \\ 0, & 0, & 0, & a \\ a, & 0, & 0, & 0 \\ 0, & a, & 0, & 0 \end{bmatrix} \\
\widehat{\boldsymbol{C}}^{(3)} &= \frac{1}{4^{3-2}(3-2)!} \begin{bmatrix} a^*, & 2a^*, & 0, & 0 \\ 2a^*, & a^*, & 0, & 0 \\ 0, & 0, & a^*, & 2a^* \\ 0, & 0, & 2a^*, & a^* \end{bmatrix}
\end{aligned}
. \tag{131}
$$

We utilize the $\mathrm{SNR_{BH}}$ based on $\widehat{\boldsymbol{C}}^{(\kappa)}$ as shown in (71), as

$$
\mathrm{SNR_{BH}} := \frac{\left(\sum_{\kappa\in\mathcal{K}} \widehat{c}^{(\kappa)}_{\mathrm{in}} - \widehat{c}^{(\kappa)}_{\mathrm{out}}\right)^2}{q^2 \sum_{\kappa\in\mathcal{K}} (\kappa-1) d^{(\kappa)}} \ , \tag{132}
$$

where $d^{(\kappa)} = \frac{\widehat{c}^{(\kappa)}_{\mathrm{in}} + (q-1)\widehat{c}^{(\kappa)}_{\mathrm{out}}}{q(\kappa-1)}$. This formulation allows us to leverage techniques from the detectability analysis of hierarchical community structures on network (25). The finest 4 communities $\{0,1,2,3\}$ and two coarse-grained community structure, the κ-dominated $\{\{0,2\},\{1,3\}\}$, the κ^*-dominated $\{\{0,1\},\{2,3\}\}$, form two hierarchical community structure. We can aggregate the matrix $\widehat{\boldsymbol{C}}^{(2)}$ and $\widehat{\boldsymbol{C}}^{(3)}$ by different coarser communities to form 2×2 matrices, and then derive their corresponding $\mathrm{SNR_{BH\,02;13}}$ and $\mathrm{SNR_{BH\,01;23}}$ based on (132). Although $\mathrm{SNR_{BH\,02;13}} = 1$ or $\mathrm{SNR_{BH\,01;23}} = 1$ can not rigorously be the detectability limit for κ or κ^* dominated community structure, the switching point between

the these two competing community structure can be approximated by solving for the equality $\mathrm{SNR}_{\mathrm{BH}\ 02;13} = \mathrm{SNR}_{\mathrm{BH}\ 01;23}$.

For the κ^* dominated community structure, the aggregation process of $\widehat{\boldsymbol{C}}$ is by averaging each block defined by the community partition $\{\{0,1\},\{2,3\}\}$. Using the matrices in (131), the aggregated matrices $\widehat{\boldsymbol{C}}_{(\kappa^*)}$ are thus calculated as:

$$\begin{aligned} \widehat{\boldsymbol{C}}^{(2)}_{(\kappa^*)} &= \begin{bmatrix} 0, & a/2 \\ a/2, & 0 \end{bmatrix} \\ \widehat{\boldsymbol{C}}^{(3)}_{(\kappa^*)} &= \frac{1}{4}\begin{bmatrix} \frac{3}{2}a^*, & 0 \\ 0, & \frac{3}{2}a^* \end{bmatrix} \end{aligned} . \tag{133}$$

The average κ-degree $d^{(\kappa)}$ can be calculated by the average of one row of $\frac{1}{\kappa-1}\widehat{\boldsymbol{C}}^{(\kappa)}$, which remains constant regardless of the aggregation process above:

$$\begin{aligned} d^{(2)} &= \frac{a}{4} \\ d^{(3)} &= \frac{3a^*}{32} \end{aligned} . \tag{134}$$

Then based on (132), we have

$$\mathrm{SNR}_{\mathrm{BH}\ 01;23} = \frac{(-\frac{a}{2} + \frac{3}{8}a^*)^2}{2^2[(2-1)d^{(2)} + (3-1)d^{(3)}]} . \tag{135}$$

Similarly, for the κ-dominated community structure $\{\{0,2\},\{1,3\}\}$, the aggregated matrices are:

$$\begin{aligned} \widehat{\boldsymbol{C}}^{(2)}_{(\kappa)} &= \begin{bmatrix} a/2, & 0 \\ 0, & a/2 \end{bmatrix} \\ \widehat{\boldsymbol{C}}^{(3)}_{(\kappa)} &= \frac{1}{4}\begin{bmatrix} \frac{1}{2}a^*, & a^* \\ a^*, & \frac{1}{2}a^* \end{bmatrix} \end{aligned} , \tag{136}$$

leading to

$$\mathrm{SNR}_{\mathrm{BH}\ 02;13} = \frac{(\frac{a}{2} + \frac{1}{8}a^* - \frac{1}{4}a^*)^2}{2^2[(2-1)d^{(2)} + (3-1)d^{(3)}]} . \tag{137}$$

Solving for the switching point $\mathrm{SNR}_{\mathrm{BH}\ 01;23} = \mathrm{SNR}_{\mathrm{BH}\ 02;13}$:

$$\begin{aligned} (-\frac{a}{2} + \frac{3a^*}{8})^2 &= (\frac{a}{2} + \frac{a^*}{8} - \frac{a^*}{4})^2 \\ (\frac{3a^*}{8})^2 - \frac{3}{8}aa^* &= (\frac{a^*}{8})^2 - \frac{1}{8}aa^* \\ \frac{1}{8}(a^*)^2 - \frac{1}{4}aa^* &= 0 \\ \frac{a}{a^*} &= \frac{1}{2} \end{aligned} . \tag{138}$$

Thus, the theoretical switching condition for the affinity tensor parameters is $\frac{a}{a^*} = \frac{1}{2}$.

To determine the corresponding hyperedge ratio $\rho = |\mathcal{E}^{(2)}|/|\mathcal{E}^{(3)}|$, we estimate $|\mathcal{E}^{(\kappa)}|$ using the approximate formula:

$$\begin{aligned} |\mathcal{E}^{(\kappa)}| &= 2\left[\binom{2n/q}{\kappa} - 2\binom{n/q}{\kappa}\right]\frac{a}{n^{\kappa-1}} \\ &\approx \frac{2a}{n^{\kappa-1}\kappa!}\left[(2n/q)^{\kappa} - 2(n/q)^{\kappa}\right] \\ &= \frac{2an}{q^{\kappa}\kappa!}(2^{\kappa}-2) \end{aligned} \quad . \tag{139}$$

Substituting $\kappa = 2$, $\kappa^* = 3$ and $q = 4$, we have

$$\rho^*_{theo} = \frac{|\mathcal{E}^{(2)}|}{|\mathcal{E}^{(3)}|} = \frac{2a/(4^2 2!)}{6a^*/(4^3 3!)} = 4\frac{a}{a^*} = 2 \ . \tag{140}$$

The theoretical switching point in terms of the hyperedge ratio is $\rho^*_{theo} = 2$.

To experimentally observe the transition, we conducted a simulation using Bethe Hessian based-spectral clustering to detect 2 communities on hypergraphs generated by fixing the total average degree d and varying the ratio ρ. By definition:

$$\begin{aligned} d &= \frac{\kappa|\mathcal{E}^{(\kappa)}| + \kappa^*|\mathcal{E}^{(\kappa^*)}|}{n} \\ \rho &= \frac{|\mathcal{E}^{(\kappa)}|}{|\mathcal{E}^{(\kappa^*)}|} \end{aligned} \quad . \tag{141}$$

Solving these relations for the hyperedge counts, we get:

$$\begin{aligned} |\mathcal{E}^{(\kappa^*)}| &= \frac{nd}{\kappa\rho + \kappa^*} \\ |\mathcal{E}^{(\kappa)}| &= \frac{nd\rho}{\kappa\rho + \kappa^*} \end{aligned} \quad . \tag{142}$$

Using the estimation of $|\mathcal{E}^{(\kappa)}|$ in (139), we derive the affinity tensor parameters a and a^*:

$$\begin{aligned} a &= \frac{4^{\kappa}\kappa! d\rho}{2(2^{\kappa}-2)(\kappa\rho + \kappa^*)} \\ a^* &= \frac{4^{\kappa^*}\kappa^*! d}{2(2^{\kappa^*}-2)(\kappa\rho + \kappa^*)} \end{aligned} \quad . \tag{143}$$

Based on these derived values for a and a^*, we set up the affinity tensor and generated the hypergraph for community detection. The results are shown in Figure S12.

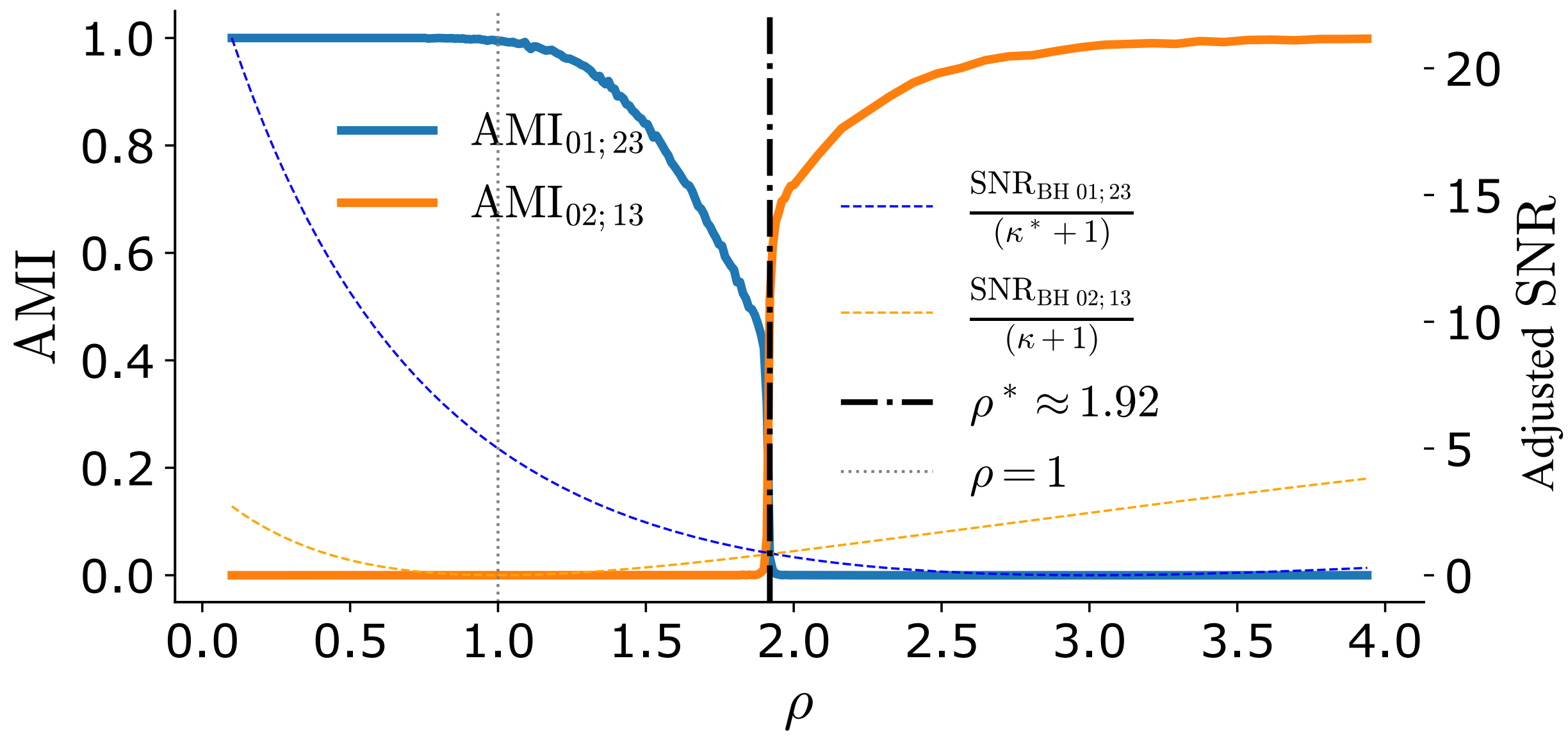

Figure S12: ***Hyperedge order preferences in community detection.*** *Experimental results with $n = 8000$, $d = 50$, $\kappa = 2$ and $\kappa^* = 3$. We show the $\mathrm{AMI}_{01;23}$ and $\mathrm{AMI}_{02;13}$ as the AMI of detected communities by Bethe Hessian based-spectral clustering to the κ^* and κ dominated community structure.*

We observed that the derived ρ^*_{theo} has a little bias to the sharp transition point. Instead, the transition point ρ^* can be better aligned by the cross point of two manually modified $\mathrm{SNR}_{\mathrm{BH}\ 01;23}$ and $\mathrm{SNR}_{\mathrm{BH}\ 02;13}$:

$$\frac{\mathrm{SNR}_{\mathrm{BH}\ 01;23}}{\kappa^*+1} = \frac{\mathrm{SNR}_{\mathrm{BH}\ 02;13}}{\kappa+1} \quad . \tag{144}$$

Next, we proceed to consider a more general case with arbitrary κ and κ^*.

S8.2 General case with arbitrary κ, κ^*

Similar as last section, we first define the affinity tensor under scenario of arbitrary κ, κ^*:

$$\begin{aligned} \boldsymbol{C}_{\boldsymbol{z}}^{(\kappa)} &= \begin{cases} a, & \boldsymbol{z} \in \left\{ \{(0,2)^\kappa/(0)^\kappa, (2)^\kappa\}, \{(1,3)^\kappa/(1)^\kappa, (3)^\kappa\} \right\} \\ 0, & otherwise \end{cases} \\ \boldsymbol{C}_{\boldsymbol{z}}^{(\kappa^*)} &= \begin{cases} a^*, & \boldsymbol{z} \in \left\{ \left\{(0,1)^{\kappa^*}/(0)^{\kappa^*}, (1)^{\kappa^*}\right\}, \left\{(2,3)^{\kappa^*}/(2)^{\kappa^*}, (3)^{\kappa^*}\right\} \right\} \\ 0, & otherwise \end{cases} \end{aligned} , \tag{145}$$

where $(...)^\kappa$ means all κ-length permutations of index, such as $(0,2)^\kappa$ is all κ-length index which exclusively include 0 or 2, $(0)^\kappa$ is $\left\{ \underbrace{(0,0,..,0)}_{\kappa} \right\}$. Then we aggregate all other

$\kappa - 2$ dimension of tensor $\frac{1}{q^{\kappa-2}(\kappa-2)!}\boldsymbol{C}^{(\kappa)}$ and $\frac{1}{q^{\kappa^*-2}(\kappa^*-2)!}\boldsymbol{C}^{(\kappa^*)}$, derive the matrices $\widehat{\boldsymbol{C}}^{(\kappa)}$ and $\widehat{\boldsymbol{C}}^{(\kappa^*)}$:

$$
\begin{aligned}
\widehat{\boldsymbol{C}}^{(\kappa)} &= \frac{1}{4^{\kappa-2}(\kappa-2)!}\begin{bmatrix} 2^{\kappa-2}-1, & 0, & 2^{\kappa-2}, & 0 \\ 0, & 2^{\kappa-2}-1, & 0, & 2^{\kappa-2} \\ 2^{\kappa-2}, & 0, & 2^{\kappa-2}-1, & 0 \\ 0, & 2^{\kappa-2}, & 0, & 2^{\kappa-2}-1 \end{bmatrix} a \\
\widehat{\boldsymbol{C}}^{(\kappa^*)} &= \frac{1}{4^{\kappa^*-2}(\kappa^*-2)!}\begin{bmatrix} 2^{\kappa^*-2}-1, & 2^{\kappa^*-2}, & 0, & 0 \\ 2^{\kappa^*-2}, & 2^{\kappa^*-2}-1, & 0, & 0 \\ 0, & 0, & 2^{\kappa^*-2}-1, & 2^{\kappa^*-2} \\ 0, & 0, & 2^{\kappa^*-2}, & 2^{\kappa^*-2}-1 \end{bmatrix} a^*
\end{aligned} \quad . \tag{146}
$$

Before aggregation with different coarse-grained community structure, we first derive $d^{(\kappa)}$ and d^{κ^*} since they remain constant after the aggregation:

$$
\begin{aligned}
d^{(\kappa)} &= \frac{1}{\kappa-1}\frac{a}{4^{\kappa-2}(\kappa-2)!}\frac{2 * 2^{\kappa-2}-1}{4} \\
&= \frac{a}{4^{\kappa-1}(\kappa-1)!}(2^{\kappa-1}-1) \\
d^{(\kappa^*)} &= \frac{a^*}{4^{\kappa^*-1}(\kappa^*-1)!}(2^{\kappa^*-1}-1)
\end{aligned} \quad . \tag{147}
$$

For κ^*-dominated community structure, $\{\{0,1\},\{2,3\}\}$, the aggregated matrices $\widehat{\boldsymbol{C}}_{(\kappa^*)}$ are:

$$
\begin{aligned}
\widehat{\boldsymbol{C}}^{(\kappa)}_{(\kappa^*)} &= \frac{a}{4^{\kappa-2}(\kappa-2)!}\begin{bmatrix} (2^{\kappa-2}-1)/2, & (2^{\kappa-2})/2 \\ (2^{\kappa-2})/2, & (2^{\kappa-2}-1)/2 \end{bmatrix} \\
\widehat{\boldsymbol{C}}^{(\kappa^*)}_{(\kappa^*)} &= \frac{a^*}{4^{\kappa^*-2}(\kappa^*-2)!}\begin{bmatrix} 2^{\kappa^*-2}-\frac{1}{2}, & 0 \\ 0, & 2^{\kappa^*-2}-\frac{1}{2} \end{bmatrix}
\end{aligned} \quad , \tag{148}
$$

leading to

$$
\mathrm{SNR}_{\mathrm{BH}\ 01;23} = \frac{\left[-\frac{a}{4^{\kappa-2}(\kappa-2)!}\frac{1}{2} + \frac{a^*}{4^{\kappa^*-2}(\kappa^*-2)!}(2^{\kappa^*-2}-\frac{1}{2})\right]^2}{2^2[(\kappa-1)d^{(\kappa)} + (\kappa^*-1)d^{(\kappa^*)}]} \quad . \tag{149}
$$

For κ-dominated community structure, $\{\{0,2\},\{1,3\}\}$, the aggregated matrices $\widehat{\boldsymbol{C}}_{(\kappa)}$ are:

$$
\begin{aligned}
\widehat{\boldsymbol{C}}^{(\kappa)}_{(\kappa)} &= \frac{a}{4^{\kappa-2}(\kappa-2)!}\begin{bmatrix} 2^{\kappa-2}-\frac{1}{2}, & 0 \\ 0, & 2^{\kappa-2}-\frac{1}{2} \end{bmatrix} \\
\widehat{\boldsymbol{C}}^{(\kappa^*)}_{(\kappa)} &= \frac{a^*}{4^{\kappa^*-2}(\kappa^*-2)!}\begin{bmatrix} (2^{\kappa^*-2}-1)/2, & (2^{\kappa^*-2})/2 \\ (2^{\kappa^*-2})/2, & (2^{\kappa^*-2}-1)/2 \end{bmatrix}
\end{aligned} \quad , \tag{150}
$$

leading to

$$\mathrm{SNR}_{\mathrm{BH}\ 02;13} = \frac{\left[\frac{a}{4^{\kappa-2}(\kappa-2)!}(2^{\kappa-2}-\frac{1}{2}) - \frac{a^*}{4^{\kappa^*-2}(\kappa^*-2)!}\frac{1}{2}\right]^2}{2^2[(\kappa-1)d^{(\kappa)}+(\kappa^*-1)d^{(\kappa^*)}]} \quad . \tag{151}$$

Next, we solve for the switching point $\mathrm{SNR}_{\mathrm{BH}\ 01;23} = \mathrm{SNR}_{\mathrm{BH}\ 02;13}$. For convenience, we define

$$\begin{aligned} b &= \frac{a}{4^{\kappa-2}(\kappa-2)!} \\ b^* &= \frac{a^*}{4^{\kappa^*-2}(\kappa^*-2)!} \end{aligned} \quad , \tag{152}$$

then

$$\begin{aligned} \mathrm{SNR}_{\mathrm{BH}\ 01;23} &= \mathrm{SNR}_{\mathrm{BH}\ 02;13} \\ (b^*2^{\kappa^*-2} - \frac{b}{2} - \frac{b^*}{2})^2 &= (b2^{\kappa-2} - \frac{b}{2} - \frac{b^*}{2})^2 \\ (b^*2^{\kappa^*-2})^2 - 2^{\kappa^*-2}bb^* - 2^{\kappa^*-2}(b^*)^2 &= (b2^{\kappa-2})^2 - 2^{\kappa-2}bb^* - 2^{\kappa-2}(b)^2 \\ 2^{\kappa^*-2}(2^{\kappa^*-2}-1)(b^*)^2 - (2^{\kappa^*-2} - 2^{\kappa-2})bb^* - 2^{\kappa-2}(2^{\kappa-2}-1)b^2 &= 0 \\ (2^{\kappa^*-2}b^* - 2^{\kappa-2}b)\underbrace{[(2^{\kappa^*-2}-1)b^* + (2^{\kappa-2}-1)b]}_{+} &= 0 \\ \frac{b}{b^*} &= \frac{2^{\kappa^*-2}}{2^{\kappa-2}} \\ \frac{a}{a^*}\frac{4^{\kappa^*-2}(\kappa^*-2)!}{4^{\kappa-2}(\kappa-2)!} &= \frac{2^{\kappa^*-2}}{2^{\kappa-2}} \\ \frac{a}{a^*} = \frac{2^{\kappa-2}(\kappa-2)!}{2^{\kappa^*-2}(\kappa^*-2)!} & \end{aligned} \quad . \tag{153}$$

Based on the formula for $|\mathcal{E}^{(\kappa)}|$ in (139), the hyperedge ratio at the switching point is

$$\begin{aligned} \rho^*_{theo} = \frac{|\mathcal{E}^{(\kappa)}|}{|\mathcal{E}^{(\kappa^*)}|} &= \frac{\frac{2an}{q^\kappa \kappa!}(2^\kappa-2)}{\frac{2a^*n}{q^{\kappa^*}\kappa^*!}(2^{\kappa^*}-2)} \\ &= \frac{(2^\kappa-2)/(4^\kappa\kappa!)}{(2^{\kappa^*}-2)/(4^{\kappa^*}\kappa^*!)}\frac{a}{a^*} \\ &= \frac{2^{\kappa-2}(\kappa-2)!(2^\kappa-2)/(4^\kappa\kappa!)}{2^{\kappa^*-2}(\kappa^*-2)!(2^{\kappa^*}-2)/(4^{\kappa^*}\kappa^*!)} \\ &= \frac{(2^\kappa-2)/(2^{\kappa+2}\kappa(\kappa-1))}{(2^{\kappa^*}-2)/(2^{\kappa^*+2}\kappa^*(\kappa^*-1))} \\ &= 2^{\kappa^*-\kappa}\frac{2^{\kappa-1}-1}{2^{\kappa^*-1}-1}\frac{\binom{\kappa^*}{2}}{\binom{\kappa}{2}} \end{aligned} \quad . \tag{154}$$

However, we still find this switching point has a little bias to the actual transition point, which can be better aligned by:

$$\frac{\mathrm{SNR}_{\mathrm{BH}\ 01;23}}{\kappa^*+1} = \frac{\mathrm{SNR}_{\mathrm{BH}\ 02;13}}{\kappa+1}\ , \tag{155}$$

as shown in Figure S13.

S8.3 Order trade-offs for Other Method

Although we primarily examine the order trade-offs of the Bethe Hessian, our intent is to show that such trade-offs are intrinsic to hypergraph community detection in general. We demonstrated this by the Hypergraph Laplacian (44) in Figure S14. Clearly, the Laplacian method also possesses a bias toward keeping higher-order hyperedges within communities, albeit with a different switching point compared to our proposed Bethe Hessian method.

S8.4 Community structure dominated by hyperedge shape

Beyond the order of hyperedges, their "shape"—the way nodes are distributed across communities—also significantly influences the detection of coarse-grained community structures. To investigate this, we designed an experiment using a hypergraph with a finest community structure of four communities $\{0, 1, 2, 3\}$, where all hyperedges are of order 4. We define two distinct hyperedge shapes based on their node distribution:

- Balanced hyperedges: These 4-order hyperedges span two communities by connecting two nodes from one community and two from another. In our model, these only exist between the community pairs $\{0, 1\}$ or $\{2, 3\}$.

- Imbalanced hyperedges: These 4-order hyperedges span two communities by connecting one node from one community and three from another. In our model, these only exist between the community pairs $\{0, 2\}$ or $\{1, 3\}$.

This setup, illustrated in Figure S15 (**A**) , creates two competing coarse-grained community structures: one based on balanced hyperedges $\{\{0, 1\}, \{2, 3\}\}$ and another based on imbalanced hyperedges $\{\{0, 2\}, \{1, 3\}\}$.

To generate this hypergraph using the HSBM, we define the affinity tensor $\boldsymbol{C}^{(4)}$ as follows. Let a^* be the affinity parameter for balanced hyperedges and a for imbalanced

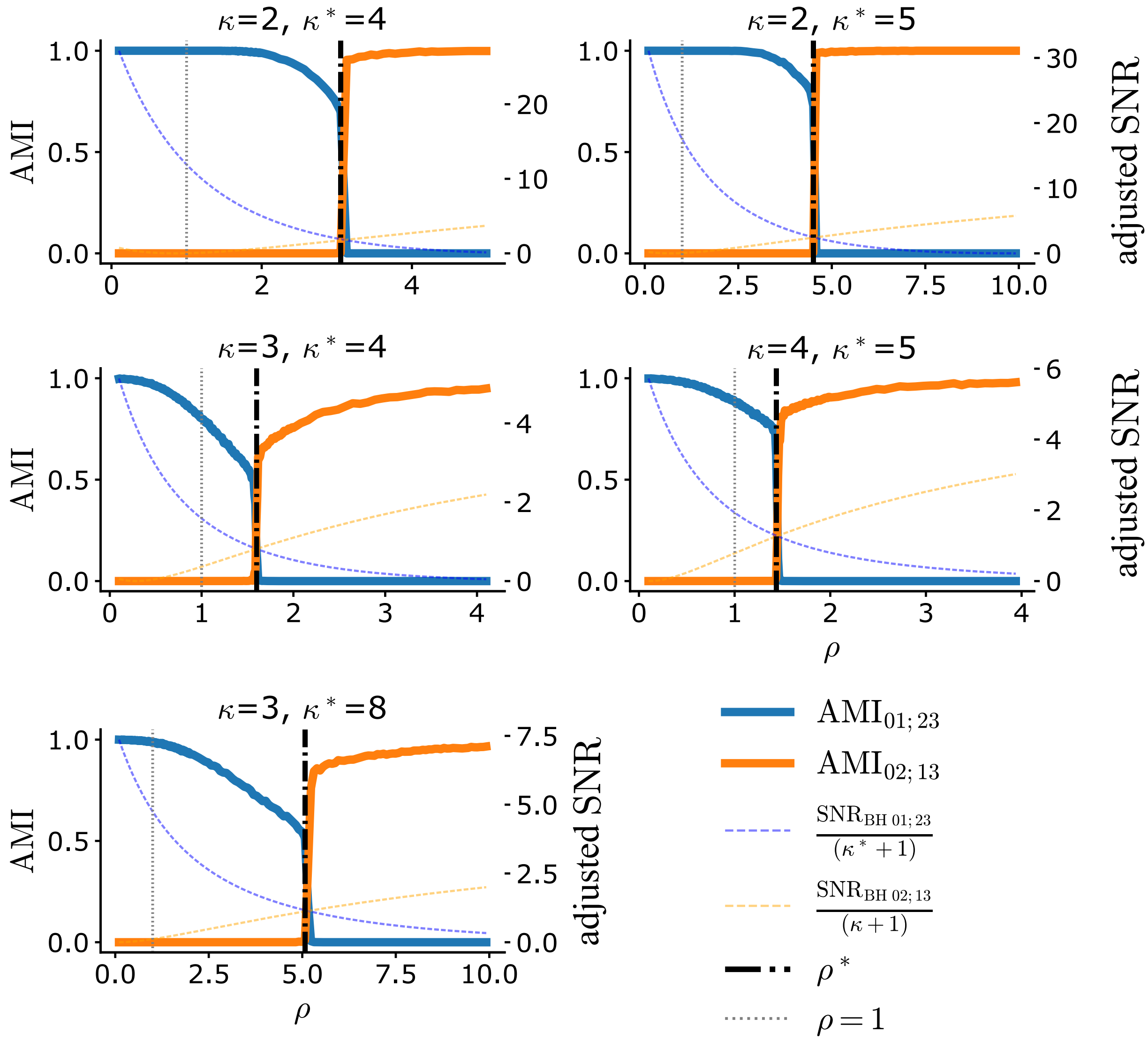

Figure S13: ***Hyperedge order preferences in community detection.*** *Experimental results with general κ and κ^*. All experiments are proceed on $n = 8000$ hypergraphs. For first row, the average degree $d = 50$ for better observing the sharp transition. For others $d = 10$ is enough. We show the $\mathrm{AMI}_{01;23}$ and $\mathrm{AMI}_{02;13}$ as the AMI of detected communities by Bethe Hessian based-spectral clustering to the κ^* and κ dominated community structure.*

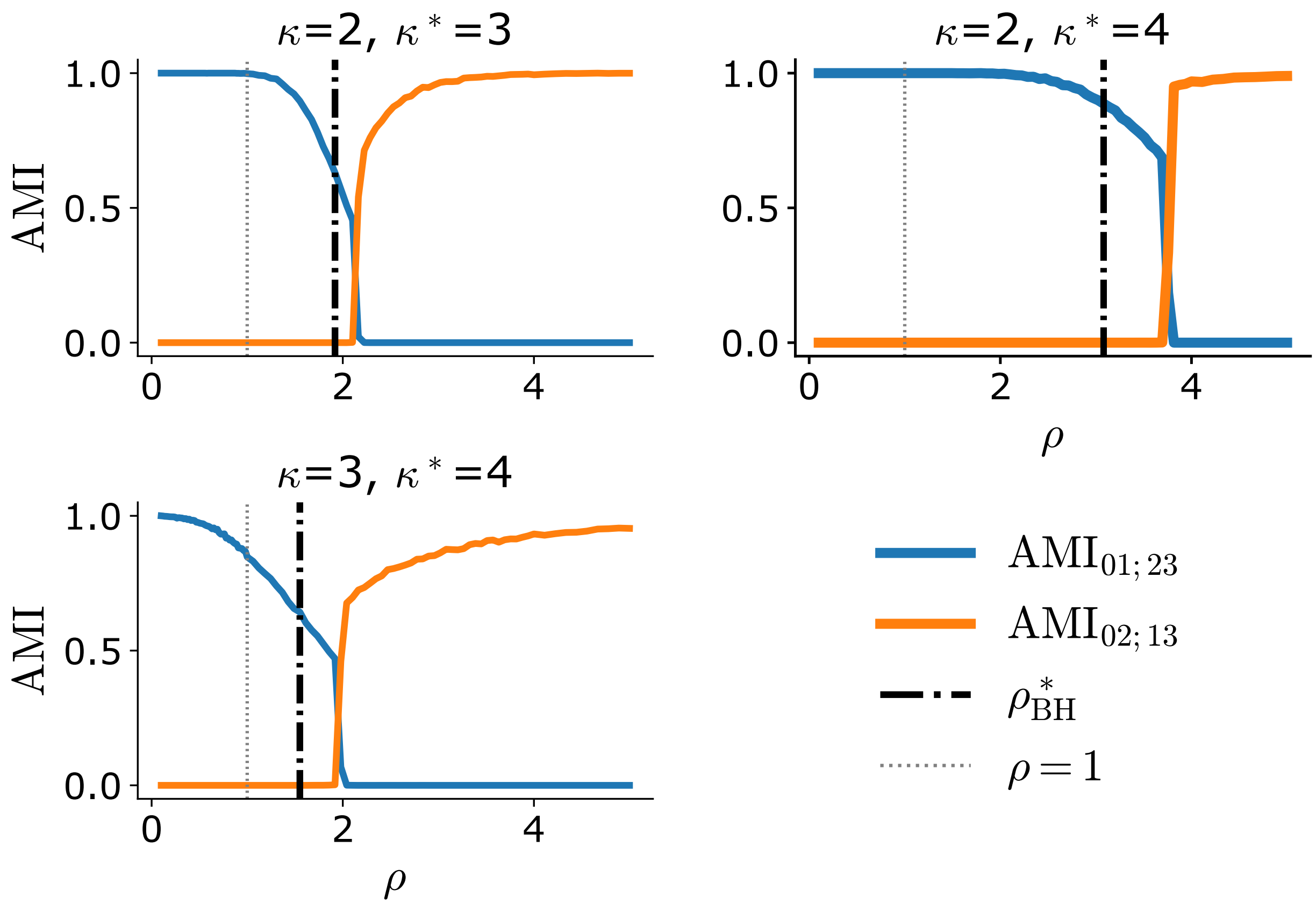

Figure S14: ***Laplacian spectral clustering transitions under competing hyperedge orders.*** *Experimental results with Laplacian based spectral clustering. All experiments are proceed on hypergraphs with $n = 8000$. For first row, the average degree $d = 50$ for better observing the sharp transition. For second row, $d = 10$ is enough. We show the $\mathrm{AMI}_{01;23}$ and $\mathrm{AMI}_{02;13}$ as the* AMI *of detected communities by Laplacian based-spectral clustering to the κ^* and κ dominated community structure. We also show the switching point of Bethe Hessian method ρ^*_{BH} for comparison.*

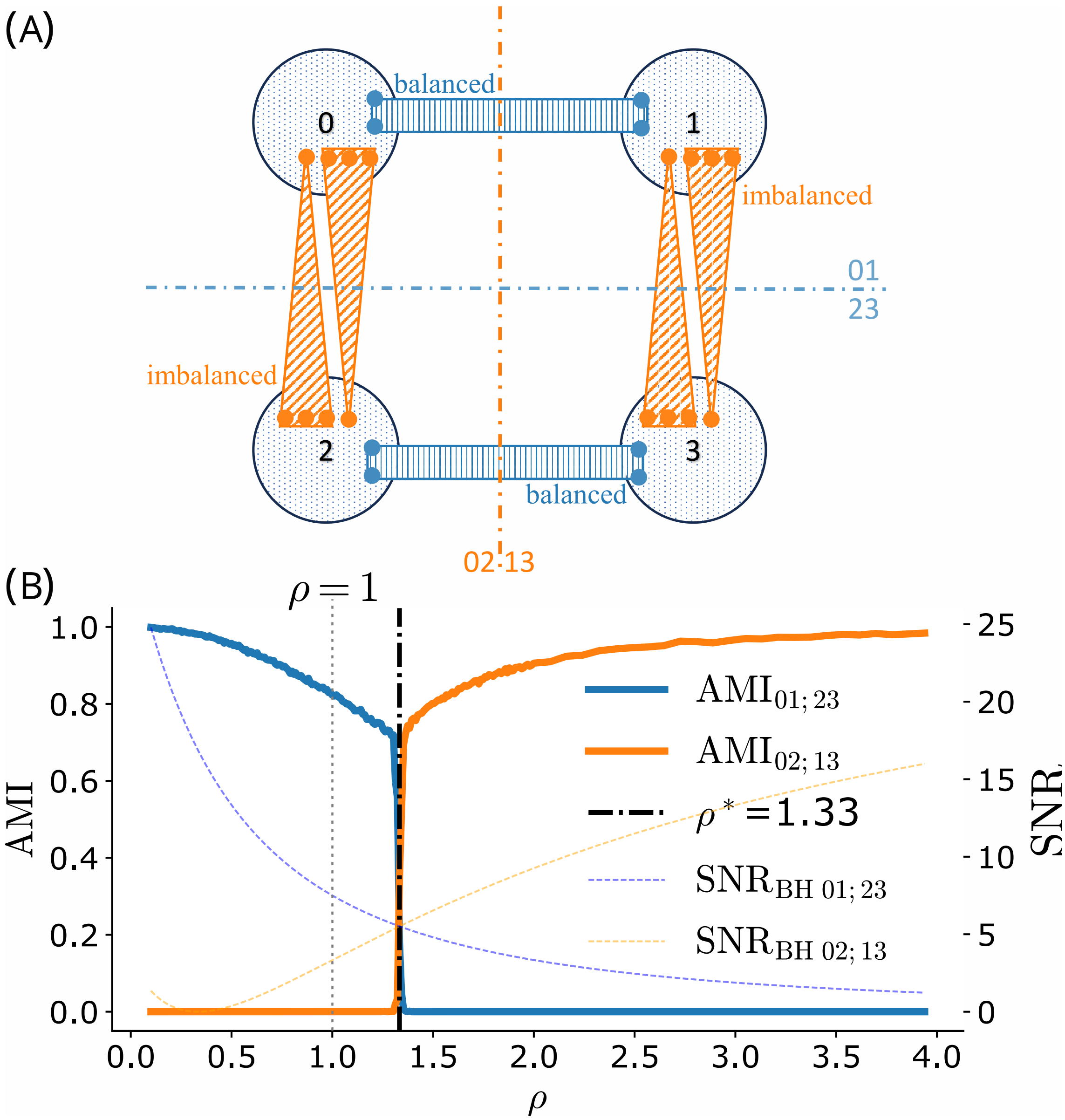

Figure S15: ***Hyperedge shape preferences in community detection.*** *(**A**) Illustration of community structure of hypergraph used in experiment for exploring the effect of shape to detectability. All the hyperedges are 4-order, only the different shape. The hyperedges shaped by rectangle between communities* $\{0,1\}$ *or* $\{2,3\}$ *are balanced, the hyperedges shaped by triangle between communities* $\{0,2\}$ *or* $\{1,3\}$ *are imbalanced. (**B**) Experimental results with* $n = 8000$, $d = 10$, $\kappa = 4$ *and* $\kappa^* = 4$. *We show the* AMI_{κ^*} *and* AMI_{κ} *as the* AMI *of detected communities by Bethe Hessian based-spectral clustering to community structure dominated by balanced and imbalanced 4-order hyperedges.*

ones:

$$\boldsymbol{C}_{\boldsymbol{z}}^{(4)} = \begin{cases} a, & \boldsymbol{z} \in \begin{matrix} \{(0,0,0,2),(0,0,2,0),(0,2,0,0),(2,0,0,0) \\ (0,2,2,2),(2,0,2,2),(2,2,0,2),(2,2,2,0) \\ (1,1,1,3),(1,1,3,1),(1,3,1,1),(3,1,1,1) \\ (3,3,3,1),(3,3,1,3),(3,1,3,3),(1,3,3,3)\} \end{matrix} \\ a^*, & \boldsymbol{z} \in \begin{matrix} \{(0,0,1,1),(0,1,1,0),(0,1,0,1), \\ (1,0,0,1),(1,1,0,0),(1,0,1,0), \\ (2,2,3,3),(2,3,2,3),(2,3,3,2), \\ (3,2,2,3),(3,2,3,2),(3,3,2,2)\} \end{matrix} \\ 0, & otherwise \end{cases} . \tag{156}$$

For consistency, we denote the expected number of balanced hyperedges as $|\mathcal{E}^{(\kappa^*)}|$ and imbalanced hyperedges as $|\mathcal{E}^{(\kappa)}|$. These can be calculated as:

$$\begin{aligned} |\mathcal{E}^{(\kappa^*)}| &= 2\binom{n/q}{2}\binom{n/q}{2}\frac{a^*}{n^3} = 2\frac{(n/q)^4}{4}\frac{a^*}{n^3} = 2\frac{na^*}{4*4^4} \\ |\mathcal{E}^{(\kappa)}| &= 4\binom{n/q}{3}\binom{n/q}{1}\frac{a}{n^3} = 4\frac{(n/q)^4}{6}\frac{a}{n^3} = 4\frac{na}{6*4^4} \end{aligned} . \tag{157}$$

Using the definitions (141) of average degree d and the hyperedge ratio $\rho = |\mathcal{E}^{(\kappa)}|/|\mathcal{E}^{(\kappa^*)}|$ from previous section, we can express the parameters a and a^* as:

$$\begin{aligned} a &= \frac{6*4^4 d\rho}{4(4\rho+4)} = \frac{6*4^2 d\rho}{\rho+1} \\ a^* &= \frac{4*4^4 d}{2(4\rho+4)} = \frac{2*4^3 d}{\rho+1} \end{aligned} . \tag{158}$$

As shown in the experimental results in Figure S15 (**B**) , the Bethe Hessian-based spectral clustering algorithm's preference switches from the balanced to the imbalanced community structure at a point $\rho^*_{theo} \neq 1$. This empirically confirms that the hyperedge shape directly affects community detectability. To theoretically predict this switching point, we analyze under similar routine to previous sections.

First, we derive the matrix $\widehat{\boldsymbol{C}}^{(4)}$:

$$\widehat{\boldsymbol{C}}^{(4)} = \frac{1}{4^{4-2}(4-2)!}\begin{bmatrix} a^*+2a, & 2a^*, & 2a, & 0 \\ 2a^*, & a^*+2a, & 0, & 2a \\ 2a, & 0, & a^*+2a, & 2a^* \\ 0, & 2a, & 2a^*, & a^*+2a \end{bmatrix} . \tag{159}$$

Aggregating this matrix according to the community structure dominated by balanced 4-order hyperedge $\{\{0,1\},\{2,3\}\}$ yields:

$$\widehat{\boldsymbol{C}}^{(4)}_{(\kappa^*)} = \frac{1}{32}\begin{bmatrix} \frac{3}{2}a^*+a, & a \\ a, & \frac{3}{2}a^*+a \end{bmatrix} \Rightarrow \mathrm{SNR}_{\mathrm{BH}\ 01;23} = \frac{1}{2^2(4-1)d^{(4)}}\left(\frac{1}{32}\frac{3}{2}a^*\right)^2 . \tag{160}$$

Similarly, aggregating for the community structure dominated by imbalanced 4-order hyperedge $\{\{0,2\},\{1,3\}\}$ gives:

$$\widehat{\boldsymbol{C}}^{(4)}_{(\kappa)} = \frac{1}{32}\begin{bmatrix} \frac{1}{2}a^* + 2a, & a^* \\ a^*, & \frac{1}{2}a^* + 2a \end{bmatrix} \Rightarrow \mathrm{SNR}_{\mathrm{BH}\ 02;13} = \frac{1}{2^2(4-1)d^{(4)}}\left(\frac{1}{32}(2a - \frac{1}{2}a^*)\right)^2 . \tag{161}$$

To find the switching point, we solve the $\mathrm{SNR}_{\mathrm{BH}\ 01;23} = \mathrm{SNR}_{\mathrm{BH}\ 02;13}$:

$$\begin{aligned} \mathrm{SNR}_{\mathrm{BH}\ 01;23} &= \mathrm{SNR}_{\mathrm{BH}\ 02;13} \\ (\frac{3}{2}a^*)^2 &= (2a - \frac{1}{2}a^*)^2 \\ 2(a^*)^2 - 4a^2 + 2aa^* &= 0 \\ (a^* - a)(a^* + 2a) &= 0 \\ a &= a^* \end{aligned} . \tag{162}$$

Last, to find the corresponding ratio of hyperedge counts ρ^*_{theo}, we substitute $a = a^*$ into the ratio formula:

$$\begin{aligned} \rho^*_{theo} = \frac{|\mathcal{E}^{(\kappa)}|}{|\mathcal{E}^{(\kappa^*)}|} &= \frac{4\binom{n/q}{3}\binom{n/q}{1}\frac{a}{n^3}}{2\binom{n/q}{2}\binom{n/q}{2}\frac{a}{n^3}} \\ &\approx \frac{\frac{(n/q)^4}{3}}{\frac{(n/q)^4}{4}} \\ &= \frac{4}{3} \end{aligned} . \tag{163}$$

This theoretical value of $\rho^*_{theo} = \frac{4}{3} \approx 1.33$ perfectly aligns with the switching point observed in our experiments (Figure S15 (**B**)). This means that balanced 4-order hyperedges provide a stronger community signal than imbalanced ones.

We also proceed similar experiment with 5-order hyperedges, as illustrated in Figure S16 (**A**) . We define the two distinct 5-order hyperedge shapes based on their node distribution:

- More Balanced hyperedges: These 5-order hyperedges span two communities by connecting two nodes from one community and three from another. In our model, these only exist between the community pairs $\{0,1\}$ or $\{2,3\}$.
- More Imbalanced hyperedges: These 5-order hyperedges span two communities by connecting one node from one community and four from another. In our model, these only exist between the community pairs $\{0,2\}$ or $\{1,3\}$.

Based on the above setup, we define the affinity tensor $\boldsymbol{C}^{(5)}$ as follows. Let a^* be the

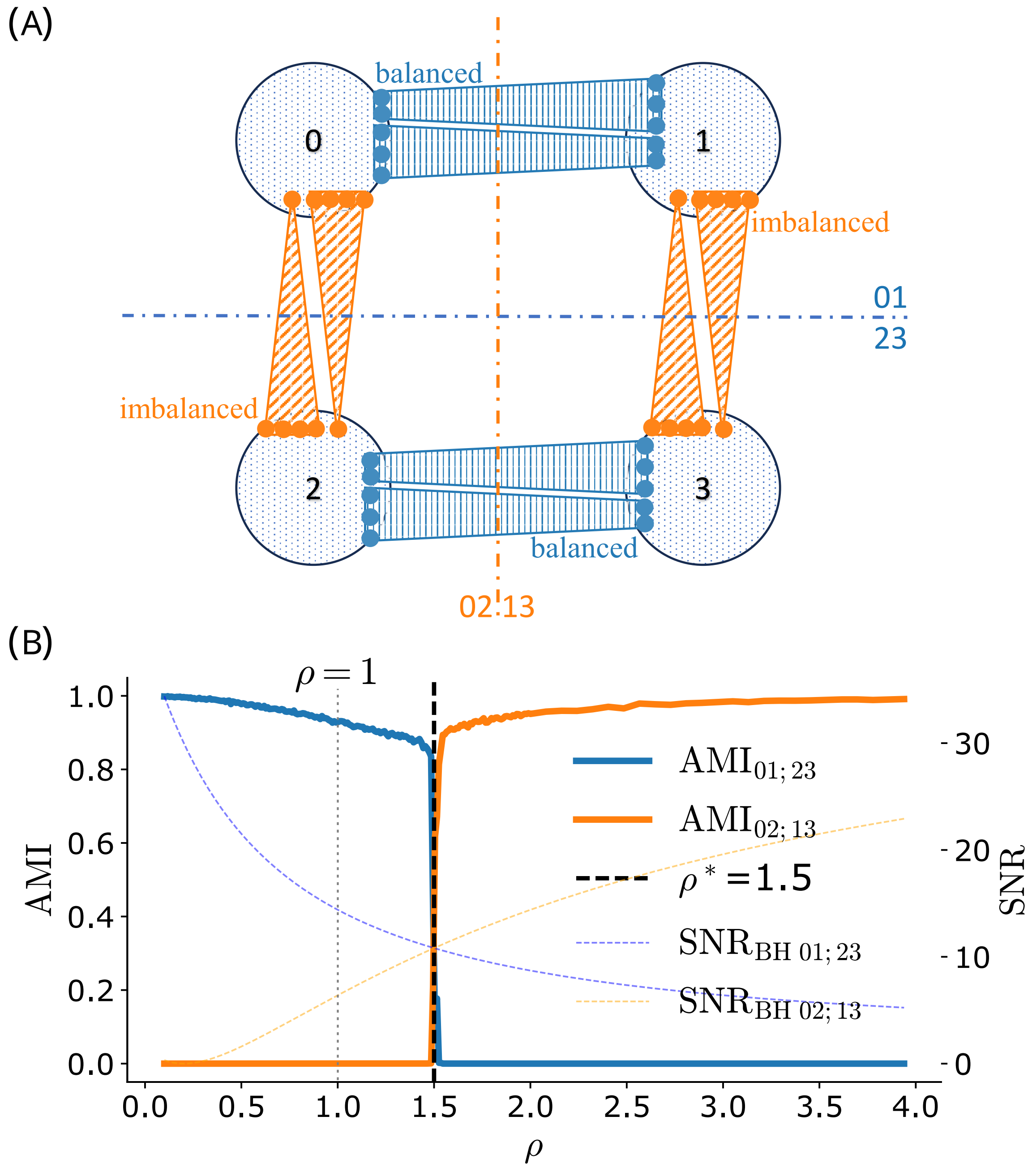

Figure S16: ***Hyperedge shape preferences in community detection.*** *(**A**) Illustration of community structure of hypergraph used in experiment for exploring the effect of shape to detectability. All the hyperedges are 5-order, only the different shape. The hyperedges shaped by trapezoid between communities* $\{0,1\}$ *or* $\{2,3\}$ *are more balanced, composed by 2 nodes in one community, 3 nodes in another. The hyperedges shaped by triangle between communities* $\{0,2\}$ *or* $\{1,3\}$ *are more imbalanced, composed by 1 node in one community, 4 nodes in another. (**B**) Experimental results with* $n = 8000$, $d = 10$, $\kappa = 5$ *and* $\kappa^* = 5$. *We show the* AMI_{κ^*} *and* AMI_{κ} *as the* AMI *of detected communities by Bethe Hessian based-spectral clustering to community structure dominated by more balanced and more imbalanced 5-order hyperedges.*

affinity parameter for more balanced hyperedges and a for more imbalanced ones:

$$\boldsymbol{C}_{\boldsymbol{z}}^{(5)} = \begin{cases} a, & \boldsymbol{z} \in \{\boldsymbol{z} | \#\{0 \text{ in } \boldsymbol{z}\} = 3, \#\{2 \text{ in } \boldsymbol{z}\} = 2\} \\ a, & \boldsymbol{z} \in \{\boldsymbol{z} | \#\{0 \text{ in } \boldsymbol{z}\} = 2, \#\{2 \text{ in } \boldsymbol{z}\} = 3\} \\ a, & \boldsymbol{z} \in \{\boldsymbol{z} | \#\{1 \text{ in } \boldsymbol{z}\} = 3, \#\{3 \text{ in } \boldsymbol{z}\} = 2\} \\ a, & \boldsymbol{z} \in \{\boldsymbol{z} | \#\{1 \text{ in } \boldsymbol{z}\} = 2, \#\{3 \text{ in } \boldsymbol{z}\} = 3\} \\ a^*, & \boldsymbol{z} \in \{\boldsymbol{z} | \#\{0 \text{ in } \boldsymbol{z}\} = 3, \#\{1 \text{ in } \boldsymbol{z}\} = 2\} \\ a^*, & \boldsymbol{z} \in \{\boldsymbol{z} | \#\{0 \text{ in } \boldsymbol{z}\} = 2, \#\{1 \text{ in } \boldsymbol{z}\} = 3\} \\ a^*, & \boldsymbol{z} \in \{\boldsymbol{z} | \#\{2 \text{ in } \boldsymbol{z}\} = 3, \#\{3 \text{ in } \boldsymbol{z}\} = 2\} \\ a^*, & \boldsymbol{z} \in \{\boldsymbol{z} | \#\{2 \text{ in } \boldsymbol{z}\} = 2, \#\{3 \text{ in } \boldsymbol{z}\} = 3\} \\ 0, & \textit{otherwise} \end{cases} . \tag{164}$$

For consistency, we denote the expected number of more balanced hyperedges as $|\mathcal{E}^{(\kappa^*)}|$ and more imbalanced hyperedges as $|\mathcal{E}^{(\kappa)}|$. These can be calculated as:

$$\begin{aligned} |\mathcal{E}^{(\kappa^*)}| &= 4\binom{n/q}{2}\binom{n/q}{3}\frac{a^*}{n^4} = 4\frac{(n/q)^5}{2!3!}\frac{a^*}{n^4} = 4\frac{na^*}{2!3!q^5} \\ |\mathcal{E}^{(\kappa)}| &= 4\binom{n/q}{4}\binom{n/q}{1}\frac{a}{n^4} = 4\frac{(n/q)^5}{4!}\frac{a}{n^4} = 4\frac{na}{4!q^5} \end{aligned} . \tag{165}$$

Using the definitions (141) of average degree d and the hyperedge ratio $\rho = |\mathcal{E}^{(\kappa)}|/|\mathcal{E}^{(\kappa^*)}|$, we can express the parameters a and a^* as:

$$\begin{aligned} a &= \frac{4^4 4! d\rho}{5(\rho+1)} \\ a^* &= \frac{4^4 2!3! d}{5(\rho+1)} \end{aligned} . \tag{166}$$

As shown in the experimental results in Figure S16 (**B**) , the Bethe Hessian-based spectral clustering algorithm's preference switches from the more balanced to the more imbalanced community structure at a point $\rho^*_{theo} \neq 1$. This further empirically confirms that the hyperedge shape directly affects community detectability. To theoretically predict this switching point, we analyze under similar routine to previous sections.

First, we derive the matrix $\widehat{\boldsymbol{C}}^{(4)}$:

$$\widehat{\boldsymbol{C}}^{(5)} = \frac{1}{4^{5-2}(5-2)!}\begin{bmatrix} 4a^*+3a, & 6a^*, & 2a, & 0 \\ 6a^*, & 4a^*+3a, & 0, & 2a \\ 2a, & 0, & 4a^*+3a, & 6a^* \\ 0, & 2a, & 6a^*, & 4a^*+3a \end{bmatrix} . \tag{167}$$

Aggregating this matrix according to the community structure dominated by more bal-

anced 5-order hyperedges $\{\{0,1\},\{2,3\}\}$ yields:

$$\begin{aligned}\widehat{\boldsymbol{C}}^{(5)}_{(\kappa^*)} =& \frac{1}{64*6}\begin{bmatrix}5a^*+\frac{3}{2}a, & a\\ a, & 5a^*+\frac{3}{2}a\end{bmatrix}\\ \Rightarrow \mathrm{SNR}_{\mathrm{BH}\ 01;23} =& \frac{1}{2^2(5-1)d^{(5)}}\left(\frac{1}{64*6}(5a^*+\frac{3}{2}a-a)\right)^2 \quad .\end{aligned} \tag{168}$$

Similarly, aggregating for the community structure dominated by more imbalanced 5-order hyperedges $\{\{0,2\},\{1,3\}\}$ gives:

$$\begin{aligned}\widehat{\boldsymbol{C}}^{(5)}_{(\kappa)} =& \frac{1}{64*6}\begin{bmatrix}2a^*+\frac{5}{2}a, & 3a^*\\ 3a^*, & 2a^*+\frac{5}{2}a\end{bmatrix}\\ \Rightarrow \mathrm{SNR}_{\mathrm{BH}\ 02;13} =& \frac{1}{2^2(5-1)d^{(5)}}\left(\frac{1}{64*6}(2a^*+\frac{5}{2}a-3a^*)\right)^2 \quad .\end{aligned} \tag{169}$$

To find the switching point, we solve the $\mathrm{SNR}_{\mathrm{BH}\ 01;23} = \mathrm{SNR}_{\mathrm{BH}\ 02;13}$:

$$\begin{aligned}\mathrm{SNR}_{\mathrm{BH}\ 01;23} &= \mathrm{SNR}_{\mathrm{BH}\ 02;13}\\ (5a^*+\frac{1}{2}a)^2 &= (\frac{5}{2}a-a^*)^2\\ (4a^*+3a)(6a^*-2a) &= 0\\ \frac{a}{a^*} &= 3\end{aligned} \quad . \tag{170}$$

Last, to find the corresponding ratio of hyperedge counts ρ^*_{theo}, we substitute $a = a^*$ into the ratio formula:

$$\rho^*_{theo} = \frac{|\mathcal{E}^{(\kappa)}|}{|\mathcal{E}^{(\kappa^*)}|} = \frac{\frac{a}{4!}}{\frac{a^*}{2!3!}} = \frac{3}{2} \quad . \tag{171}$$

This theoretical value of $\rho^*_{theo} = \frac{3}{2}$ perfectly aligns with the switching point observed in our experiments (Figure S16 (**B**)). This means that more balanced 5-order hyperedges provide a stronger community signal than more imbalanced ones.

S9 Notation

node set	$\mathcal{V}$
node	i, j, k, l
number of nodes	n
edge(hyper) set	$\mathcal{E}$
edge(hyper)	e, f, g
number of edges(hyper)	m
order of edge(hyper) e	$\lvert e \rvert$
order set	$\mathcal{K}$
order κ hyperedge set	$\mathcal{E}^{(\kappa)}$
number of order κ hyperedges	$m^{(\kappa)}$
average degree	d
diagonal degree matrix	$\boldsymbol{D}$
order κ degree of node i	$d_i^{(\kappa)}$
order κ average degree	$d^{(\kappa)}$
order κ degree matrix	$\boldsymbol{D}^{(\kappa)}$
number of communities	q
community labels	$\overrightarrow{\psi}$
adjacent matrix	$\boldsymbol{A}$
edge probability matrix(tensor)	$\boldsymbol{\Omega}$
rescaled edge probability matrix(tensor)	$\boldsymbol{C}$
incidence matrix	$\boldsymbol{H}$
order κ-incidence matrix	$\boldsymbol{H}^{(\kappa)}$
one-mode projection of $\boldsymbol{H}^{(\kappa)}$	$\boldsymbol{A}^{(\kappa)}$
non-backtracking matrix	$\boldsymbol{NB}$
non-backtracking matrix of κ-order uniform hypergraph	$\boldsymbol{NB}^{(\kappa)}$
non-backtracking matrix of nonuniform hypergraph with order set $\mathcal{K}$	$\boldsymbol{NB}^{(\mathcal{K})}$
bethe hessian matrix	$\boldsymbol{B}$
bethe hessian matrix of κ-order uniform hypergraph	$\boldsymbol{B}^{(\kappa)}$
bethe hessian matrix of nonuniform hypergraph with order set $\mathcal{K}$	$\boldsymbol{B}^{(\mathcal{K})}$

Table S1: Notations

REFERENCES

1. S. Fortunato, Community detection in graphs. *Phys. Rep.* **486**, 75–174 (2010).

2. M. A. Porter, J.-P. Onnela, P. J. Mucha, Communities in networks. *Not. Am. Math. Soc.* **56**, 1082–1097 (2009).

3. V. D. Blondel, J.-L. Guillaume, R. Lambiotte, E. Lefebvre, Fast unfolding of communities in large networks. *J. Stat. Mech.* **2008**, P10008 (2008).

4. U. Von Luxburg, A tutorial on spectral clustering. *Stat. Comput.* **17**, 395–416 (2007).

5. P. W. Holland, K. B. Laskey, S. Leinhardt, Stochastic blockmodels: First steps. *Soc. Netw.* **5**, 109–137 (1983).

6. A. Decelle, F. Krzakala, C. Moore, L. Zdeborová, Asymptotic analysis of the stochastic block model for modular networks and its algorithmic applications. *Phys. Rev. E* **84**, 066106 (2011).

7. F. Krzakala, C. Moore, E. Mossel, J. Neeman, A. Sly, L. Zdeborová, P. Zhang, Spectral redemption in clustering sparse networks. *Proc. Natl. Acad. Sci. U.S.A.* **110**, 20935–20940 (2013).

8. A. Saade, F. Krzakala, L. Zdeborová, Spectral clustering of graphs with the Bethe Hessian. *Adv. Neural Inf. Process. Syst.* **27**, 406–414 (2014).

9. C. Bick, E. Gross, H. A. Harrington, M. T. Schaub, What are higher-order networks? *SIAM Rev.* **65**, 686–731 (2023).

10. M. Newman, *Networks* (Oxford Univ. Press, ed. 2, 2018).

11. A. Sanchez-Gorostiaga, D. Bajić, M. L. Osborne, J. F. Poyatos, A. Sanchez, High-order interactions distort the functional landscape of microbial consortia. *PLoS Biol.* **17**, e3000550 (2019).

12. F. Battiston, G. Cencetti, I. Iacopini, V. Latora, M. Lucas, A. Patania, J.-G. Young, G. Petri, Networks beyond pairwise interactions: Structure and dynamics. *Phys. Rep.* **874**, 1–92 (2020).

13. F. Battiston, E. Amico, A. Barrat, G. Bianconi, G. Ferraz de Arruda, B. Franceschiello, I. Iacopini, S. Kéfi, V. Latora, Y. Moreno, M. M. Murray, T. P. Peixoto, F. Vaccarino, G. Petri, The physics of higher-order interactions in complex systems. *Nat. Phys.* **17**, 1093–1098 (2021).

14. P. S. Chodrow, N. Veldt, A. R. Benson, Generative hypergraph clustering: From blockmodels to modularity. *Sci. Adv.* **7**, eabh1303 (2021).

15. D. Ghoshdastidar, A. Dukkipati, Consistency of spectral hypergraph parti-tioning under planted partition model. *Ann. Stat.* **45**, 289–315 (2017).

16. P. Chodrow, N. Eikmeier, J. Haddock, Nonbacktracking spectral clustering of nonuniform hypergraphs. *SIAM J. Math. Data Sci.* **5**, 251–279 (2023).

17. M. C. Angelini, F. Caltagirone, F. Krzakala, L. Zdeborová, "Spectral detection on sparse hypergraphs," in *2015 53rd Annual Allerton Conference on Communication, Control, and Computing (Allerton)* (IEEE, 2015), pp. 66–73.

18. N. Ruggeri, A. Lonardi, C. De Bacco, Message-passing on hypergraphs: De-tectability, phase transitions and higher-order information. *J. Stat. Mech.* **2024**, 043403 (2024).

19. L. Stephan, Y. Zhu, Sparse random hypergraphs: Non-backtracking spectra and community detection. *Inf. Inference* **13**, iaae004 (2024).

20. D. Ghoshdastidar, A. Dukkipati, "Consistency of spectral partitioning of uniform hypergraphs under planted partition model," in *Advances in Neural Information Processing Systems 27* (Curran Associates, Inc., 2014).

21. J. Li, HyperGraphBetheHessian (GitHub, 2026). https://github.com/eggplantisme/HyperGraphBetheHessian.

22. E. Mossel, J. Neeman, A. Sly, Reconstruction and estimation in the planted partition model. *Probab. Theory Relat. Fields* **162**, 431–461 (2015).

23. L. Massoulié, “Community detection thresholds and the weak Ramanujan property,” in *46th Annual Symposium on the Theory of Computing* [Association for Computing Machinery (ACM), 2014], pp. 694–703.

24. E. Abbe, Community detection and stochastic block models: Recent developments. *J. Mach. Learn. Res.* **18**, 1–86 (2018).

25. L. Peel, M. T. Schaub, Detectability of hierarchical communities in networks. *Phys. Rev. E* **110**, 034306 (2024).

26. J. Li, L. Peel, Detectability of minority communities in networks. arXiv:2604.18565 [cs.SI] (2026).

27. N. X. Vinh, J. Epps, J. Bailey, “Information theoretic measures for clusterings comparison: Is a correction for chance necessary?” in *Proceedings of the 26th Annual International Conference on Machine Learning* (Association for Computing Machinery, 2009), pp. 1073–1080.

28. J. Stehlé, N. Voirin, A. Barrat, C. Cattuto, L. Isella, J.-F. Pinton, M. Quaggiotto, W. Van den Broeck, C. Régis, B. Lina, P. Vanhems, High-resolution mea-surements of face-to-face contact patterns in a primary school. *PLOS ONE* **6**, e23176 (2011).

29. R. Mastrandrea, J. Fournet, A. Barrat, Contact patterns in a high school: A comparison between data collected using wearable sensors, contact diaries and friendship surveys. *PLOS ONE* **10**, e0136497 (2015).

30. Yelp Open Dataset, https://business.yelp.com/data/resources/open-dataset/.

31. contact-primary-school dataset (labeled), https://cs.cornell.edu/∼arb/data/contact-primary-school-labeled/.

32. N. Tremblay, P. Borgnat, Graph wavelets for multiscale community mining. *IEEE Trans. Signal Process.* **62**, 5227–5239 (2014).

33. contact-high-school dataset (labeled), https://cs.cornell.edu/~arb/data/contact-high-school-labeled/.

34. M. T. Schaub, J. Li, L. Peel, Hierarchical community structure in networks. *Phys. Rev. E* **107**, 054305 (2023).

35. L. Peel, D. B. Larremore, A. Clauset, The ground truth about metadata and community detection in networks. *Sci. Adv.* **3**, e1602548 (2017).

36. F. Radicchi, Detectability of communities in heterogeneous networks. *Phys. Rev. E* **88**, 010801 (2013).

37. P. Zhang, C. Moore, M. Newman, Community detection in networks with unequal groups. *Phys. Rev. E* **93**, 012303 (2016).

38. L. Dall'Amico, R. Couillet, N. Tremblay, "Revisiting the Bethe-Hessian: Improved community detection in sparse heterogeneous graphs," in *Advances in Neural Information Processing Systems 32* (Curran Associates, Inc., 2019).

39. P. Erdős, A. Rényi, On random graphs I. *Publ. Math. Debrecen* **6**, 18 (1959).

40. C. Moore. The computer science and physics of community detection: Landscapes, phase transitions, and hardness. arXiv:1702.00467 [cs.CC] (2017).

41. A. Saade, Spectral inference methods on sparse graphs: Theory and applications. arXiv:1610.04337 [cond-mat.dis-nn] (2016).

42. G. H. Golub, C. F. Van Loan, *Matrix Computations* (JHU Press, 2012).

43. M. Mezard, A. Montanari, *Information, Physics, and Computation* (Oxford Univ. Press, 2009).

44. D. Zhou, J. Huang, B. Schölkopf, "Learning with hypergraphs: Clustering, classification, and embedding," *Advances in Neural Information Processing systems 19* (Curran Associates, Inc., 2006).

45. B. Kamiński, P. Misiorek, P. Pra-lat, F. Théberge, Modularity based community detection in hypergraphs. *J. Complex Netw.* **12**, cnae041 (2024).
